# Common Inherited Variation in Mitochondrial Genes Is Not Enriched for Associations with Type 2 Diabetes or Related Glycemic Traits

**DOI:** 10.1371/journal.pgen.1001058

**Published:** 2010-08-12

**Authors:** Ayellet V. Segrè, DIAGRAM Consortium, MAGIC investigators, Leif Groop, Vamsi K. Mootha, Mark J. Daly, David Altshuler

**Affiliations:** 1 Broad Institute of Harvard and Massachusetts Institute of Technology, Cambridge, Massachusetts, United States of America; 2 Center for Human Genetic Research, Massachusetts General Hospital, Boston, Massachusetts, United States of America; 3 Department of Molecular Biology, Massachusetts General Hospital, Boston, Massachusetts, United States of America; 4 Department of Clinical Sciences, Diabetes and Endocrinology Research Unit, University Hospital Malmö, Lund University, Malmö, Sweden; 5 Department of Systems Biology, Harvard Medical School, Boston, Massachusetts, United States of America; 6 Department of Medicine, Harvard Medical School, Boston, Massachusetts, United States of America; 7 Diabetes Unit, Massachusetts General Hospital, Boston, Massachusetts, United States of America; 8 Department of Genetics, Harvard Medical School, Boston, Massachusetts, United States of America; Queensland Institute of Medical Research, Australia

## Abstract

Mitochondrial dysfunction has been observed in skeletal muscle of people with diabetes and insulin-resistant individuals. Furthermore, inherited mutations in mitochondrial DNA can cause a rare form of diabetes. However, it is unclear whether mitochondrial dysfunction is a primary cause of the common form of diabetes. To date, common genetic variants robustly associated with type 2 diabetes (T2D) are not known to affect mitochondrial function. One possibility is that multiple mitochondrial genes contain modest genetic effects that collectively influence T2D risk. To test this hypothesis we developed a method named Meta-Analysis Gene-set Enrichment of variaNT Associations (MAGENTA; http://www.broadinstitute.org/mpg/magenta). MAGENTA, in analogy to Gene Set Enrichment Analysis, tests whether sets of functionally related genes are enriched for associations with a polygenic disease or trait. MAGENTA was specifically designed to exploit the statistical power of large genome-wide association (GWA) study meta-analyses whose individual genotypes are not available. This is achieved by combining variant association *p*-values into gene scores and then correcting for confounders, such as gene size, variant number, and linkage disequilibrium properties. Using simulations, we determined the range of parameters for which MAGENTA can detect associations likely missed by single-marker analysis. We verified MAGENTA's performance on empirical data by identifying known relevant pathways in lipid and lipoprotein GWA meta-analyses. We then tested our mitochondrial hypothesis by applying MAGENTA to three gene sets: nuclear regulators of mitochondrial genes, oxidative phosphorylation genes, and ∼1,000 nuclear-encoded mitochondrial genes. The analysis was performed using the most recent T2D GWA meta-analysis of 47,117 people and meta-analyses of seven diabetes-related glycemic traits (up to 46,186 non-diabetic individuals). This well-powered analysis found no significant enrichment of associations to T2D or any of the glycemic traits in any of the gene sets tested. These results suggest that common variants affecting nuclear-encoded mitochondrial genes have at most a small genetic contribution to T2D susceptibility.

## Author Summary

Mitochondria play a crucial role in metabolic homeostasis, and alteration of mitochondrial function is a hallmark of diabetes. While mitochondrial activity is reduced in people with diabetes, it is unclear whether mitochondrial dysfunction is a cause or effect of type 2 diabetes. Genome-wide association studies for type 2 diabetes have explained ≈10% of the heritability of the disease, but none of the loci are known to affect mitochondrial activity. It is possible though that a mitochondrial contribution is hidden in the remaining 90%. Hence, we tested the hypothesis that multiple mitochondria-related genes encoded in the nucleus, each having a weak effect (hard to detect individually), can collectively influence type 2 diabetes. To address this, we developed a computational method (MAGENTA) that allowed us to adequately analyze large collective datasets of human genetic variation obtained from collaborative studies of type 2 diabetes and related glycemic traits. Despite the increased sensitivity of MAGENTA compared to single-DNA variant analysis, we found no support for a causal relationship between mitochondrial dysfunction and type 2 diabetes. These results may help steer future efforts in understanding the pathogenesis of the disease. MAGENTA is broadly applicable to testing associations between other biological pathways and common diseases or traits.

## Introduction

Mitochondrial dysfunction has been implicated in both rare and common forms of type 2 diabetes (T2D) [1]–[4]. Individuals with T2D contain less mitochondria in their skeletal muscle [5], [6], and impaired mitochondrial function has been associated with T2D and insulin resistance, an intermediate phenotype and risk factor of diabetes [7]. In particular, oxidative phosphorylation (OXPHOS) activity in mitochondria, central for energy production in the cell, is reduced in certain populations of diabetic and insulin-resistant individuals [5], [8]. Furthermore, we found that the expression of OXPHOS genes is coordinately downregulated in diabetic versus healthy muscle [9], [10]. It has been proposed that decreased OXPHOS activity may contribute to T2D development by causing fatty acid accumulation in muscle cells, which in turn may inhibit insulin-stimulated glucose uptake [1], [2], [7], [8], [11], or by indirectly reducing glucose-stimulated insulin secretion from pancreatic ß-cells due to a decrease in ATP production [1]. However, it is still not clear whether the molecular and physiologic associations of mitochondria with diabetes are a cause or effect of the common form of T2D [1], [2], [12].

One way to test whether mitochondrial genes play a causal role in the pathogenesis of T2D is to search for inherited DNA variants in mitochondrial genes that influence T2D risk. Proof of concept comes from rare mutations in mitochondrial DNA (mtDNA) that cause Maternally Inherited Diabetes with Deafness (OMIM #520000). This raises the question of whether inherited variants affecting mitochondrial biology play a more general causal role in the common form of T2D. Candidate gene studies of mitochondria-related genes have yet to conclusively demonstrate (at genome-wide significance) that common variants in nuclear-encoded mitochondrial genes or transcriptional regulators of mitochondrial genes associate with T2D risk [13]–[15]. Also, we published a systematic scan for associations of common single-nucleotide polymorphisms (SNPs) in mtDNA (that encodes 13 genes) that failed to identify significant associations with T2D [16].

Recently, several genome-wide association (GWA) studies of ∼2,000 to 5,000 individuals [17]–[19], and a meta-analysis of 10,128 individuals [20], all of European descent, have identified ∼18 common nuclear DNA variants robustly associated with T2D that collectively explain ∼6% of the genetic contribution to T2D risk. While these associations suggest genes involved in various biological processes, such as WNT signaling, NOTCH signaling and the cell cycle, none have implicated mitochondrial processes. The only gene with a mitochondrial isoform near a validated T2D SNP is the insulin-degrading enzyme, *IDE* (Entrez ID 3416), but it exerts its insulin degrading activity primarily in the cytoplasm [21].

Given the large number of nuclear-encoded mitochondrial genes (∼1,000 known based on the mitochondrial protein compendium MitoCarta [22]) and the largely unexplained genetic basis of T2D, it is possible that many (tens or hundreds of) common variants in or near mitochondrial genes are associated with T2D. While each gene might have a modest effect too small to be detected on its own, together they could have a more substantial collective impact. It is also possible that several nuclear regulators of mitochondrial genes could harbor common variants of modest effects on T2D risk.

These hypotheses could be tested using a Gene Set Enrichment Analysis (GSEA) approach applied to genetic variant association data [23]. We originally described GSEA to test whether predefined biological processes or gene sets are enriched for genes with coordinate modest expression differences between two samples, differences that are hard to detect when inspecting each gene separately [9], [24]. In fact, GSEA was first used to show that OXPHOS genes are collectively downregulated in human muscle in diabetic compared to non-diabetic individuals [9].

In the context of genetic association data, GSEA has been suggested to be a promising approach to identify sets of functionally related genes, such as biological pathways, enriched for associations of modest effects (hard to detect with single-marker analysis) on a polygenic disease or trait [23]. Several groups have begun to apply different variations of GSEA to GWA studies to study disorders such as Parkinson's disease [23], dyslipidemia [25], T2D [26]–[28], Crohn's disease [25], [29], [30], and multiple sclerosis [31]. While the principal concept is similar in these studies, alternative implementations differ substantially, for example in how genes are scored or enrichment is evaluated. In addition, researchers have only begun to evaluate the ranges of parameters (e.g. effect size or fraction of causal genes) under which gene-set approaches have power to identify associations not found by single-variant analysis [32], [33].

To maximize power, it is critical to make use of meta-analyses of multiple independent GWA studies whose increasing sample size (from thousands of people in single studies to tens of thousands in meta-analyses) boosts the statistical power for detecting clustering of modest associations. Yet, as opposed to traditional GWA studies, information about individual genotypes is not available for most meta-analyses, making it impossible to evaluate statistical significance through standard phenotype permutation analysis. While several GSEA variations have been recently applied to meta-analyses, the extent to which they account for confounding effects on gene association scores has not been tested.

Here we introduce a GSEA approach applied to genome-wide variant association data, which we named “Meta-Analysis Gene-set Enrichment of variaNT Associations” (MAGENTA). MAGENTA does not require genotype data, making it especially relevant to GWA study meta-analyses. We tested and validated MAGENTA using the Diabetes Genetics Initiative (DGI) GWA study [17], and three GWA meta-analyses of cholesterol and lipid blood levels [34]. Using simulations, we identified the conditions under which our method has increased power to detect associations for which there is low detection power with single SNP analysis. Finally, to test whether mitochondrial dysfunction may be causal to T2D, we applied MAGENTA to a set of known nuclear regulators of mitochondrial genes [35], the OXPHOS genes [9], and all known (∼1,000) autosomal human mitochondrial genes [22], using the latest T2D meta-analysis of a total of 47,117 individuals (DIAGRAM+) [36], as well as meta-analyses (up to 46,186 individuals) of seven glucose and insulin-related traits relevant to T2D pathogenesis (MAGIC; [37], [38], Soranzo N. *et al.*, unpublished data).

## Results

**Figure 1:**
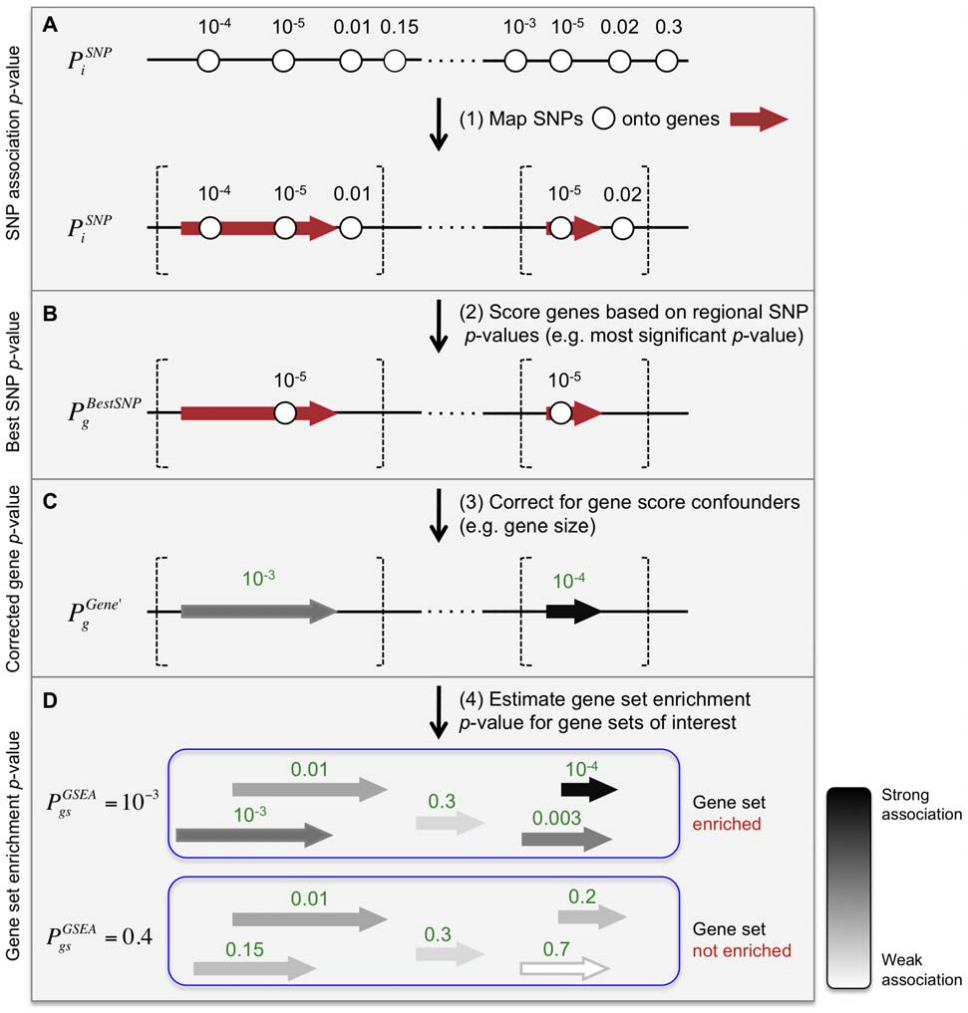
Description of Meta-Analysis Gene-set Enrichment of variaNT Associations (MAGENTA) method. (A) Step 1: Map genetic variants and their association scores onto genes. MAGENTA uses as input the association z-scores or *p*-values of DNA sequence variants across the entire genome. In this work, we used association *p*-values of single-nucleotide polymorphisms, SNPs (circles) from a genome-wide association study or meta-analysis, denoted as 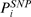 for SNP *i*. Gene boundaries (vertical dashed lines) are defined here as predetermined physical distances added upstream and downstream to the most extreme transcript start and end sites of the gene (red arrow), respectively. Linkage-based distances can also be used. Each gene is assigned a set of SNPs that fall in its gene region boundaries. Two genes are shown for simplicity. (B) Step 2: Score genes based on their local SNP 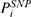. Here the most significant 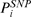 of all SNPs *i* that lie within the extended gene boundaries is assigned to each gene *g* in the genome (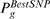). (C) Step 3: Correct for confounding effects on the gene score, 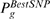 in the absence of genotype data. In this study we used step-wise multivariate linear regression analysis to regress out of 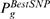 the confounding effects of several physical and genetic properties of genes (listed in Table 1); 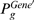 refers to the corrected gene *p*-value for gene *g*. In cases where two genes are assigned the same best SNP *p*-value, 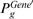 tends to be more significant for small genes than for large genes. (D) Step 4: Calculate a gene set enrichment *p*-value for each biological pathway or gene set of interest. We used a non-parametric statistical test to test whether 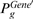 for all genes in gene set *gs* are enriched for highly ranked gene scores more than would be expected by chance, compared to randomly sampled gene sets of identical size from the genome. 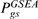 refers to the nominal gene set enrichment *p*-value for gene set *gs*.

Meta-Analysis Gene-set Enrichment of variaNT Associations (MAGENTA) evaluates pre-specified gene sets for enrichment of modest associations with a complex disease or trait. MAGENTA consists of four main steps: First, DNA variants, e.g. single-nucleotide polymorphisms (SNPs), are mapped onto genes (Figure 1A). Second, each gene is assigned a gene association score that is a function of its regional SNP association *p*-values (Figure 1B). Third, confounding effects on gene association scores are identified and corrected for, without requiring genotype data (enabling use of meta-analyses or other types of GWA studies where only variant association statistics are available) (Figure 1C). Fourth, a Gene Set Enrichment Analysis (GSEA)-like statistical test is applied to predefined biologically relevant gene sets to determine whether any of the gene sets are enriched for highly ranked gene association scores compared to randomly sampled gene sets of identical size from the genome (Figure 1D). These four steps are described below, and further detailed in the Materials and Methods section.

### From SNPs to genes: scoring genes based on SNP association scores

To analyze genetic association data at the level of genes and gene sets, we first needed to compute a gene score based on local SNPs. We assigned to each gene *g* in the genome a set of SNPs that lie within 110 kilobase (kb) upstream and 40 kb downstream of the gene's most extreme transcript boundaries, in attempt to capture signals from potential causal variants affecting regulatory elements, in addition to coding sequence (Figure 1A; see Materials and Methods for boundary choice). Each gene *g* is then assigned a score 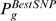, defined in this instantiation as the most significant *p*-value among the association *p*-values 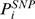 of all individual SNPs *i* within the extended gene boundaries (Figure 1B). We used the best SNP rather than an average value, as we expect only one or a few associated variants per gene.

When 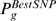 was calculated for all 966 nuclear-encoded mitochondrial genes using the T2D DIAGRAM+ GWA meta-analysis, we found that their scores were on average less significant than random (Figure S1). Observing that the mitochondrial genes are smaller on average than all other genes in the genome (Table S1), we next examined the effect of confounders on the most significant SNP *p*-value per gene, 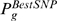. Towards this goal, we generated 1,000 null distributions of gene scores, through phenotype permutations of the Diabetes Genetics Initiative (DGI) GWA study, for which we have access to genotype data (see Materials and Methods). In these randomized data sets no genome-wide significant associations are expected. We observed significant correlations of the scores for each gene across permutations (mean Pearson's correlation coefficient across all genes for pairs of permutations: r = 0.36). This suggested that inherent properties of genes (not specific to the phenotype studied) confound gene scores.

When we explicitly tested the potential confounding role of gene size, previously suggested [23], [25], we observed that large genes tended to receive more significant scores than small genes in the randomized data set (Figure S2).

### Correcting for confounding effects on gene association scores in the absence of genotype data

When genotype data are available, such as in individual GWA studies, confounding effects on 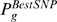, e.g. gene size, can be corrected for using phenotype permutation analysis that does not require *a priori* knowledge of the confounders (described in Materials and Methods). However, to exploit the power of large GWA study meta-analyses, where permutation analysis cannot be performed due to unavailability of genotype data, we needed an alternative correction method. We chose a linear regression-based approach that adjusts for the effects of multiple confounders on the gene score. This required identifying a substantial amount of the confounding effects on 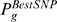.

To find confounders on 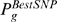 we systematically tested for correlations between the unadjusted gene score, 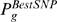 calculated from permuted DGI GWA study (see Materials and Methods) and six potential gene score confounders (listed in Table 1; correlations reported for z-scores). We examined both physical properties of genes - physical gene size and number of SNPs per kilobase for each gene, and genetic properties that consider the dependency between subsets of SNPs due to genetic linkage between proximal markers. The genetic properties tested included estimated number of independent SNPs per gene (SNPs in linkage equilibrium), number of recombination hotspots spanning each gene, genetic distance of the gene, and linkage disequilibrium (LD) unit distance per gene, normalized to the size of the gene and its extended boundaries (see Materials and Methods). We found significant correlations for all six properties tested (average values across 1,000 permuted data sets: 0.17<r<0.38; *p*<2e-70) (Table 1), suggesting that all variables may have a confounding effect on 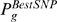. A similar trend was observed using 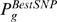 from the actual DGI GWA study (Table 1, column 2; 0.14<r<0.39, *p*<1e-74), and the T2D GWA meta-analysis, used below to test the mitochondria-diabetes hypothesis (Table S2).

**Table 1:** Correlation between type 2 diabetes gene association scores and potential gene score confounders.

	Mean across 1,000 permuted DGI GWA datasets	DGI GWA study		
Gene property	Correlation with 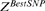 (No correction)	Correlation with 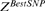 (No correction)	Correlation with 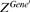 (Regression correction)	Correlation with 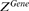 (Permutation correction)
Gene size, kilobase (kb)†	0.26	0.25	−0.03	0.01
# SNPs per kb†	0.38	0.39	−0.05	−0.02
# independent SNPs per kb†	0.32	0.31	−0.07	−0.001
# recombination hotspots per kb†	0.17	0.14	−0.04	0.01
Linkage disequilibrium units per kb*	0.22	0.19	−0.06	0.02
Genetic distance, centi-Morgan per kb	0.19	0.16	−0.05	0.03

Pearson's correlation coefficients were calculated between 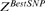, 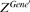 or 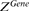 and six different physical and genetic properties of genes. 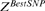 is a vector of the unadjusted best SNP per gene z-scores for all genes in the genome, 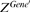 is a vector of corrected gene z-scores using regression analysis for all genes, and 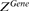 is a vector of corrected gene z-scores using phenotype permutation analysis for all genes. This was computed for 1,000 phenotype permutation data sets of the Diabetes Genetics Initiative (DGI) GWA study and the actual DGI GWA study. Aside for gene size, all gene properties were converted to per kilobase (kb) units for each gene by dividing by gene region size using the extended physical boundaries. All correlations between 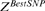 and the six variables were statistically significant (mean *p*<2e-70 across 1,000 DGI permutations and *p*<1e-74 for the actual DGI study). Similar correlations were obtained for the five latter variables in Table 1 before normalizing to gene region size (data not shown).

**†:** These gene properties were significant in almost all 1,000 DGI GWA permutations tested under a step-wise multivariate linear regression model of 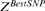 regressed against the six gene properties (see Table S3).

*The linkage disequilibrium units per kb variable was significant under the regression model for about half of the permutations tested (Table S3).

Having identified six potential gene score confounders, we used step-wise multivariate linear regression to remove these confounding effects from 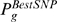, to generate a corrected gene score, 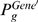 (see Materials and Methods and Figure 1C). In this analysis the confounders are removed sequentially, accounting for the correlations between the various gene properties. Aside from the genetic distance, all five remaining properties listed in Table 1 were significant under the step-wise linear regression model (*p*<0.05) applied to 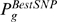 in either about half or all of the 1,000 permuted DGI data sets (Table S3). As a result the first five properties listed in Table 1 were used for subsequent analyses (see Table S4 for model coefficients and *p*-values for the DGI study and the DIAGRAM+ T2D meta-analysis).

**Figure 2:**
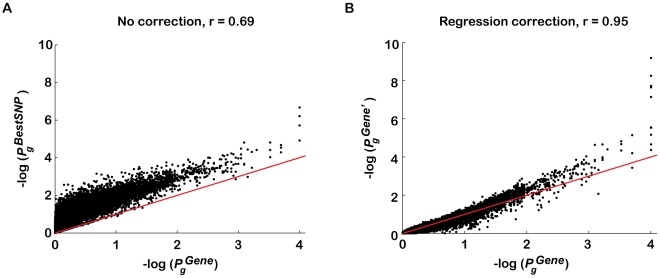
Regression analysis corrects for majority of confounding effects on gene association scores in a genotype-independent manner. The performance of a step-wise regression analysis approach in correcting for confounders on 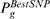 was evaluated against permutation analysis correction, since the latter corrects for all confounders without requiring *a priori* knowledge of them. T2D gene association *p*-values were plotted for all genes *g* in the genome (A) before gene score adjustment (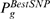) and (B) after correction for confounders using regression analysis (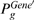), as a function of corrected gene *p*-values using phenotype permutation analysis (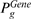). The Diabetes Genetics Initiative (DGI) GWA study was used for the analysis, since we had access to all individuals' genotypes. 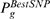 is the association *p*-value of the best regional SNP for gene *g* before correction (y-axis in A). To compute 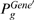 (y-axis in B), step-wise multivariate linear regression analysis was applied to 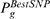 against the first four confounders listed in Table 1 (this approach does not require genotype data). The Pearson's correlation coefficient (calculated between *p*-value vectors before log transformation) increased significantly following the regression-based correction (from r = 0.69 to r = 0.95). The spread around the diagonal (red line) also decreased following the regression correction (from a coefficient of variation (mean/std) of 1.13 to 0.56). The minimum 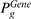 is 10^−4^ as the *p*-values were calculated based on 1,000 permutations for genes with 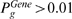, and 10,000 permutations for genes with 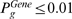. Some of the variation in the low *p*-value tail is due to having done only 10,000 permutations (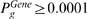), and some to limitations of the linear regression method. Note that the four dots in (A) with 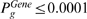 contain ten overlapping dots that refer to four sets of 2–3 genes, each set assigned the same 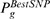. Gene association *p*-values are plotted on a −log_10_(*p*-value) scale.

The effectiveness of this approach was confirmed by comparing the DGI gene scores corrected with step-wise regression analysis to the corresponding gene scores corrected with traditional permutation analysis, as the latter corrects for all confounding effects (Figure 2; see Materials and Methods). The high correlation between the regression-corrected gene scores, 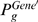 and the permutation-corrected gene scores, 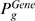 for all genes (Pearson's correlation coefficients, r = 0.95; *p*<1e-30, Figure 2B) compared to before correction (r = 0.69, Figure 2A) indicates that only a small fraction of the confounding effects on 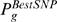 is not explained by our regression method. Similar results were obtained when gene score ranks were compared (r = 0.95 versus r = 0.82; *p*<1e-30). A comparison of the distributions of 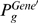 for different sized genes using the permuted DGI data sets, demonstrates that the regression-based correction has indeed removed the confounding effect of gene size on 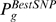 (Figure S2).

We next compared the performance of the regression-based correction to an analytical method previously proposed to correct for the difference in number of (genotyped or imputed) SNPs per gene (Sidak's correction, [26], [39]). The Sidak correction did not perform as well as the regression-based correction (correlation with permutation-corrected gene *p*-values: r = 0.94, *p*<1e-30, but most gene *p*-values lie below diagonal; see Figure S3 for details). This is probably due to the method's assumption of independence between all SNPs in a gene region (eq. 4 in Materials and Methods). We then tested a modification of Sidak's correction proposed by Saccone *et al.* [40], which assumes that about 50% of all SNPs in a given chromosomal region are in high linkage disequilibrium (eq. 5 in Materials and Methods). This correction was comparable to, or slightly better than the regression method in the DGI test case (correlation with permutation-corrected gene *p*-values: r = 0.97, *p*<1e-30; Figure S3). These results are in concordance with our findings that number or density of SNPs is a dominant confounder on the best SNP per gene score, 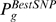 (Table 1), and that correcting for linkage disequilibrium between SNPs is necessary.

For the current study we used the regression-based correction, as it seems to behave equally well for different GWA studies (e.g. DGI study and DIAGRAM+ meta-analysis; see Figure S4A, S4B), while the modified Sidak's correction (in particular its correction for dependency between SNPs) may need to be adjusted for specific studies, e.g. due to different SNP densities (see Figure S4C, S4D). In any case, we later show that all GSEA results presented in this work are robust relative to the correction method used.

### From genes to gene sets: estimating power of MAGENTA using simulations

After correcting for the majority of confounding effects on gene association scores, we next combined gene scores at the level of gene sets. We developed an approach similar to GSEA that tests whether predefined sets of functionally related genes are enriched for genes associated with a given complex disease or phenotype, more than would be expected by chance (Figure 1D). Specifically, the GSEA algorithm in MAGENTA tests for over-representation of genes in a given gene set above a predetermined gene score rank cutoff. The enrichment is evaluated against a null distribution of gene sets of identical set size that are randomly sampled from the genome multiple times (see Materials and Methods for details). The 95^th^ percentile of all gene scores for a given GWA study or meta-analysis was used here as the enrichment cutoff (see Figure S5 for cutoff choice). Since subsets of genes in biological pathways are often physically proximal in the genome [25], for each gene set, we removed all but one gene from each subset of genes assigned the same best SNP, to prevent inflation of an enrichment signal due to positional clustering of genes (assuming one gene per associated variant).

We first evaluated the power (sensitivity) of the method to identify enrichment of modest associations. We considered models in which there is low detection power with single SNP analysis. We varied as parameters gene set size, fraction of genes in the set assigned a causal SNP (referred to as causal genes), effect size of the causal SNPs, and total number of causal genes. We performed multiple computer simulations where small effect sizes were randomly assigned to SNPs near different fractions of genes in a given gene set, against a background of randomized DGI 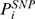 for all SNPs *i*. Power was estimated for a given set of parameters as the fraction of simulation runs in which gene set enrichment was detected (described in Materials and Methods).

**Figure 3:**
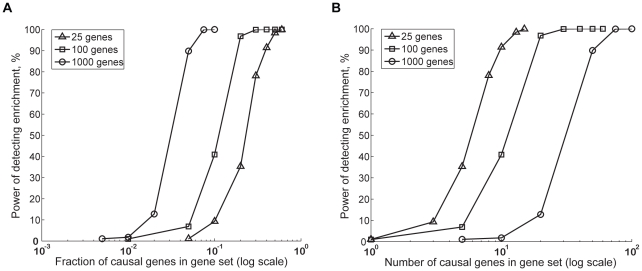
Estimating power of the GSEA algorithm in MAGENTA using computer simulations. We used simulations to assess the power (sensitivity) of the gene set enrichment analysis (GSEA) algorithm in MAGENTA to detect enrichment of genes with modest effect sizes that are hard to detect with single SNP analysis. Power is plotted as a function of fraction (A) or number (B) of causal genes of modest effect in gene sets of 25 (triangles), 100 (squares), or 1,000 (circles) genes. The modest effect size spiked into genes is equivalent to 1% power of detecting an association at genome-wide significance using single SNP analysis. A total of 100 causal genes in the genome were assumed here. Randomized 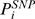 vectors from case/control permutations of the DGI study were used as the background association values. Simulations were repeated 1,000 times for each unique set of parameters. Power was calculated as the fraction of times the simulated gene set received a 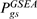
<0.01. For specificity estimations we used SNPs with no effect size, sampled from a null distribution that assumes no association. The false positive rate of the method (1-specificity) was comparable to the *p*-value cutoff used (0.3–1.7%). Note the *x*-axis in both panels is on a log_10_ scale.

Figure 3 shows how the power of MAGENTA increases proportionately with the fraction (Figure 3A) or number (Figure 3B) of causal genes in three different gene set sizes, for a set of parameters chosen to be consistent with the lower bound effect size found to date in T2D and glycemic traits studied herein. For a given number of causal genes, small gene sets are more powerful; for a given fraction of causal genes, big gene sets have more power. Furthermore, as may be expected, power increases with the associated SNP effect size, and decreases with the total number of causal genes in the genome (Figure S6).

Our simulations identified scenarios under which analysis of GWA SNP data at the level of gene sets adds power in detecting associations of small effect (e.g. odds ratio of 1.07 for an allele frequency of 0.2–0.3 and sample size of 10,000 individuals [41]) compared to single SNP analysis. For example, consider a total of 100 causal genes in the genome each with an effect size, sufficient to provide 1% power of detecting an association at the individual SNP level at genome-wide significance. In this setting, MAGENTA has 50% power of detecting enrichment if a given set of 1,000 genes (e.g. nuclear-encoded mitochondrial genes) contains ∼3% or 30 genes with a modest effect, when 100 genes (e.g. OXPHOS genes) contain ∼10% or 10 genes with a modest effect, or when 25 genes (e.g. on the order of the number of nuclear regulators of mitochondrial genes) contain ∼25% or 6 genes with a modest effect.

### Validation of MAGENTA on lipoprotein and lipid GWA study meta-analyses

We next tested empirically the performance of MAGENTA on LDL cholesterol, HDL cholesterol and triglyceride blood levels. The molecular pathways involved in lipid and lipoprotein metabolism are relatively well known, and many of the genes that lie near the 30 SNPs so far reported to be associated with one or more of these traits function in known processes related to lipid or lipoprotein metabolism [34]. MAGENTA was applied to three GWA meta-analyses [34], whose sample size (19,840 individuals) is on the same order of magnitude as that of the largest available T2D meta-analysis (DIAGRAM+) used below to test for mitochondria-related associations with T2D. A total of 51 (partially overlapping) gene sets related to lipid, lipoprotein and fatty acid metabolism were tested (defined by PANTHER [42] and Gene Ontology [43] databases; see Materials and Methods). Of these gene sets, we found biological processes related to lipid, lipoprotein and fatty acid metabolism, binding and transport activities, and triglyceride metabolism to be significantly enriched for LDL cholesterol, HDL cholesterol and/or triglyceride associations after Bonferroni correction (top gene sets are listed in Table 2; full list in Tables S5, S6, S7). These results are robust relative to the method used to correct for confounders on gene association scores (Tables S8, S9, S10). Enrichment of LDL and HDL associations in the lipid transport process has been previously reported [25]. While most of the enriched processes contain at least one gene near a validated lipid SNP, this analysis also found enrichment in a pathway with unknown associations (the fatty acid metabolic process). The fact that the enrichment signals for many of the significant processes were still detectable after removing the known lipid genes from the GSEA analysis, suggests that some of the nominally significant associations in these pathways may represent true associations of more modest effects yet to be identified.

**Table 2:** Top GSEA results for lipid-related pathways using LDL cholesterol, HDL cholesterol, and triglyceride GWA meta-analyses.

Database	Gene set	# genes analyzed by GSEA	Nominal 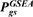	Nominal 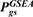 without known lipid genes	Genes near validated lipid SNPs
**Top lipid-related gene sets enriched for LDL cholesterol associations**					
GO, BP	LIPID TRANSPORT	27	0.0001*	0.0352	APOE, LDLR
GO, BP	LIPID HOMEOSTASIS	14	0.0005*	0.0204	APOE, PCSK9
GO, BP	LIPOPROTEIN METABOLIC PROCESS	31	0.0010*	0.0038	LDLR
GO, BP	LIPID METABOLIC PROCESS	291	0.0013*	0.0046	APOC1, APOC2, APOC4, LDLR
GO, BP	FATTY ACID METABOLIC PROCESS	58	0.0019*	0.0024	-
GO, BP	LIPID CATABOLIC PROCESS	36	0.0079	0.0078	-
GO, MF	LIPID TRANSPORTER ACTIVITY	27	0.0090	0.0352	APOC4
GO, MF	LIPOPROTEIN BINDING	18	0.0106	0.0466	LDLR
PANTHER	FATTY ACID METABOLISM	88	0.0120	0.0112	-
GO, BP	REGULATION OF LIPID METABOLIC PROCESS	11	0.0140	0.0143	-
**Top lipid-related gene sets enriched for HDL cholesterol associations**					
GO, BP	TRIACYLGLYCEROL METABOLIC PROCESS	9	1e-6*	8.3e-5*	APOC3, CETP, LPL, APOA5
GO, BP	LIPID TRANSPORT	27	1e-6*	0.0023	ABCA1, APOA1, APOA4, APOC3, CETP, LCAT
GO, MF	LIPID BINDING	79	1.8e-5*	0.0036*	APOA1, APOA4, CETP, APOA5
GO, BP	LIPID HOMEOSTASIS	14	1e-5*	0.0012*	ABCA1, APOA1, APOA4, CETP, LCAT
GO, MF	PHOSPHOLIPID BINDING	43	2.8e-5*	0.012	APOA1, APOA4, CETP, APOA5
PANTHER	LIPID AND FATTY ACID TRANSPORT	99	4e-5*	0.0162	ABCA1, APOA1, APOA4, APOC3, CETP, PLTP, APOA5
GO, BP	LIPID METABOLIC PROCESS	287	6e-5*	0.0179	APOA1, APOA4, APOA5, APOC3, CETP, HNF4A, LCAT, FADS1, FADS2, LPL, MVK, PLTP
GO, BP	CELLULAR LIPID METABOLIC PROCESS	229	0.0003*	0.0548	APOA1, APOC3, CETP, LCAT, FADS1, LPL
GO, MF	STEROL BINDING	9	0.0004*	0.0435	APOA1, CETP
GO, BP	LIPID CATABOLIC PROCESS	36	0.0006*	0.0068	APOA4, APOA5
GO, BP	CELLULAR LIPID CATABOLIC PROCESS	33	0.005	0.0206	APOA5
GO, BP	LIPID BIOSYNTHETIC PROCESS	87	0.0110	0.2327	APOA1, LCAT, FADS1, FADS2, MVK
**Top lipid-related gene sets enriched for triglyceride associations**					
GO, BP	LIPID HOMEOSTASIS	14	0.0001*	0.0974	APOA1, APOA4, ANGPTL3
GO, BP	TRIACYLGLYCEROL METABOLIC PROCESS	9	0.0008*	0.307	APOC3, LPL, APOA5
GO, MF	LIPID TRANSPORTER ACTIVITY	25	0.0012*	0.3238	APOA1, APOA4
GO, BP	LIPID TRANSPORT	26	0.0023	0.3154	APOA1, APOC3, ANGPTL3, APOA4
GO, BP	LIPOPROTEIN METABOLIC PROCESS	31	0.0044	0.4123	APOA1, APOA4, ANGPTL3
GO, BP	PHOSPHOLIPID METABOLIC PROCESS	69	0.0081	0.0061	APOA1, FADS1, LPL
GO, BP	LIPID CATABOLIC PROCESS	36	0.0083	0.0811	APOA4, APOA5, ANGPTL3
GO, BP	GLYCEROPHOSPHOLIPID METABOLIC PROCESS	42	0.0149	0.0036	APOA1

The most significant lipid-related biological gene sets with a gene set enrichment *p*-value of 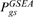
<0.015 are presented using GWA meta-analyses of LDL cholesterol, HDL cholesterol and triglyceride blood levels across a total of 19,840 individuals. Complete results for all 51 lipoprotein and lipid related pathways are presented in Tables S5, S6, S7. GSEA *p*-values marked with an asterisk are significant under a conservative Bonferroni correction (each database was corrected separately due to considerable overlap between gene sets across the different databases). The number of genes per gene set analyzed with MAGENTA in column three is after removing genes without SNPs in their extended gene boundaries and after adjusting for chromosomal proximity between subsets of genes in a gene set (see Materials and Methods). The fifth column contains GSEA *p*-values following exclusion of genes near validated SNPs for the relevant lipid trait (19 genes for LDL cholesterol, 20 genes for HDL cholesterol and 19 genes for triglyceride levels; taken from Table 2 in [34]). The sixth column lists all genes near validated lipid SNPs (as of [34]) that fall in a given gene set, including the genes removed due to adjustment for physical proximity in the genome. GO stands for Gene Ontology, BP for Biological Process, and MF for Molecular Function.

### Gene set enrichment analysis of T2D associations in mitochondria-related gene sets

Having validated the utility of MAGENTA, we next used the method to test whether mitochondria-related gene sets are enriched for multiple genes that lie near common variants with modest effects on T2D susceptibility. We tested three molecular hypotheses based on the observations of reduced OXPHOS activity and expression levels and fewer and smaller mitochondria in diabetic muscle (described in the Introduction). The three hypotheses were: DNA variants that alter the function of different nuclear regulators of the OXPHOS pathway and/or other mitochondrial processes are associated with T2D, variants that cause core defects in OXPHOS activity that may result in compensatory alterations of OXPHOS levels are associated with T2D, and variants that affect other mitochondrial functions in addition to the OXPHOS process are associated with T2D. To test these hypotheses, we tested for enrichment of T2D associations in the following three gene sets: a set of known nuclear regulators of mitochondrial genes, the OXPHOS genes, and all known nuclear-encoded mitochondrial genes. In parallel to testing the relevance of these three sets to T2D, we explored their possible associations (in non-diabetic individuals) with seven specific glycemic traits that are risk factors for T2D (listed below).

We first analyzed a set of 16 nuclear regulators of mitochondrial genes assembled based on the literature (listed in Table S11) [35], [44]–[48], using the latest DIAGRAM+ T2D GWA meta-analysis of 8,130 cases and 38,987 controls from eight GWA studies [36]. Since no individual mitochondria regulator was found to date to be significantly associated with T2D at genome-wide significance, we tested the hypothesis that common variants in more than one regulator may affect T2D risk (possibly through OXPHOS downregulation) in the diabetic populations analyzed here. Upon applying MAGENTA to the set of nuclear regulators we did not observe significant enrichment of T2D associations compared to the genomic background of gene scores (Table 3; 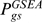 = 0.19; Quantile-quantile plot of gene *p*-values in Figure S7A). The peroxisome proliferator-activated receptor delta, *PPARD* (Entrez ID 5467) [44], received the best T2D gene *p*-value, although it was not gene-wide significant (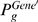 = 0.0089). The gene scores of the 16 known nuclear regulators of mitochondrial functions are listed in Table S11.

**Table 3:** Mitochondria-related gene sets are not enriched for associations with type 2 diabetes.

Gene set	Total # genes	# genes without SNPs in vicinity	# genes removed due to physical clustering in genome*	Effective # genes‡	Nominal 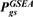
Nuclear regulators of mitochondrial genes	16	0	0	16	0.1889
Oxidative phosphorylation genes	91	0	0	91	0.4722
Nuclear-encoded mitochondrial genes	966	11	70	885	0.9125

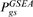
 is the nominal gene set enrichment p-value for a given gene set gs, calculated here using the DIAGRAM+ T2D GWA study meta-analysis and an enrichment cutoff that equals the 95th percentile of all gene p-values, 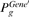.

**‡:** The effective number of genes is the number of genes analyzed after removing genes with no SNPs in their extended gene boundaries, and after correcting for chromosomal clustering of subsets of genes in a gene set, i.e. removing all but one gene of each subset of genes assigned the same best local SNP *p*-value (*).

Next, we tested for enrichment of T2D associations in a set of 91 autosomal OXPHOS genes (highlighted in the full list of mitochondrial gene scores in Table S12). Using MAGENTA, no significant enrichment of T2D associations was found among the 91 OXPHOS genes analyzed (Table 3; 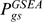 = 0.47). A plot of the OXPHOS T2D gene scores against an expected distribution of gene scores is shown in Figure S7B.

Finally, we applied MAGENTA to 966 nuclear-encoded human mitochondrial genes taken from the MitoCarta compendium (∼85% of all mitochondrial genes; see Materials and Methods) [22]. We did not observe significant enrichment of T2D associations for the whole set of mitochondrial genes either (Table 3; nominal 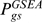 = 0.91). A more detailed view of the mitochondrial gene score distribution is shown in Figure S7C (see Table S12 for a list of all mitochondrial gene association *p*-values).

While the above findings show no evidence of association between relevant mitochondrial gene sets and T2D, these genes could still display causal associations with specific intermediate phenotypes linked to the disease. Support for this comes from reported mitochondrial dysfunction in insulin-resistant individuals [8]. Therefore, we tested the same three gene sets described above for enrichment of associations with seven different glucose and insulin-related traits characteristic of T2D, using GWA meta-analyses of up to 46,186 non-diabetic individuals [37], [38] (Soranzo N. *et al.*, unpublished data). The quantitative traits analyzed include fasting levels of glucose and insulin, glucose and insulin levels 2 hours following a 75-gram oral glucose tolerance test, indices of β-cell function (HOMA-B) and insulin resistance (HOMA-IR) [49], and glycated hemoglobin levels (HbA_1C_), which reflect long-term plasma glucose concentrations (see Materials and Methods).

No significant enrichment of genes associated with either of the seven glycemic traits tested was observed for the set of nuclear regulators of mitochondrial genes, the OXPHOS genes or the full set of nuclear-encoded mitochondrial genes, after correcting for multiple hypothesis testing (Table 4). Similar results were obtained between all three gene sets and T2D or the seven glycemic traits tested, using an alternative GSEA statistical test based on a rank-sum test (see Materials and Methods and Table S13) or using an alternative gene score correction method (modified Sidak's correction; Table S14), confirming the robustness of these results.

**Table 4:** Mitochondria-related gene sets are not enriched for associations with type 2 diabetes-related glycemic traits.

Glycemic trait	Nuclear-encoded mitochondrial genes 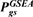	OXPHOS genes 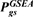	Nuclear regulators of mitochondrial genes 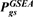
Fasting glucose	0.1255	0.8354	0.5568
Fasting insulin	0.2489	0.9490	0.1878
2 hour glucose	0.3026	0.6696	1.0000
2 hour insulin	0.2900	0.9462	1.0000
HOMA-IR	0.6567	0.9429	0.1855
HOMA-B	0.7678	0.8375	0.5661
HbA_1c_	0.0179‡	0.9901	1.0000

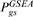
 is the nominal gene set enrichment p-value for gene set gs computed for each glycemic trait separately. The enrichment cutoff calculated for each phenotype is the 95th percentile of all gene p-values computed from the corresponding GWA study meta-analysis. HOMA-IR is an index for insulin resistance, HOMA-B is an index for ß-cell function, and HbA1c represents glycated hemoglobin concentrations, which is a measure of long-term plasma glucose concentrations.

**‡:** Not significant after Bonferroni correction (most stringent cutoff p<0.002 given 3 gene sets and 8 traits; a less stringent cutoff, *p*<0.0083 correcting for 3 gene sets and 2 traits due to correlation between the glucose and insulin-related traits).

In summary, our gene set analysis of T2D and glycemic traits did not provide support for many weak mitochondria-related associations.

## Discussion

We tested the open question of whether mitochondrial dysfunction is a primary cause of type 2 diabetes (T2D) as opposed to a secondary cause or an outcome of the disease. Using a genetic approach, we comprehensively analyzed common variant associations at the level of genes and gene sets, in search for multiple modest genetic effects on T2D pathogenesis in a set of nuclear regulators of mitochondrial activity, the oxidative phosphorylation (OXPHOS) genes, or the full known set of ∼1,000 nuclear-encoded mitochondrial genes (an estimated 85% of all mitochondrial genes). For this analysis, we developed a modified GSEA approach applied to genetic association data (*p*-values or z-scores), which we named MAGENTA. MAGENTA was especially designed to exploit the increased power of meta-analyses of multiple GWA studies. In the process we identified and adjusted for confounders on gene scores and gene set enrichment scores in the absence of genotype information. This method was rigorously tested and evaluated using real and simulated GWA data, and we demonstrate realistic scenarios in which this approach could identify significant set-wide association signal that is likely to be overlooked in individual SNP analysis.

### Identifying and correcting for confounders on SNP to gene association *p*-values

In testing for possible confounding effects, we observed that the unadjusted most significant SNP per gene *p*-value is affected by several gene properties, most notably physical gene size and number or density of SNPs per gene, and the genetic properties: number or density of SNPs across a gene that are in linkage equilibrium to each other and number or density of recombination hotspots that span a gene. While gene size and number of SNPs per gene have been recently reported to be correlated with the unadjusted best SNP *p*-value [23], [25], [33], we have quantitatively demonstrated the magnitude of these and linkage-based effects using randomized GWA study data, confirming their potential confounding effects. We show that large genes tend to receive a more significant score than small genes by chance (Figure S2).

By using regression analysis to adjust the gene scores for the confounding effects we identified, we provide a viable approach to determine gene association *p*-values in the absence of genotype data, which should prove useful for mining large GWA study meta-analyses or other types of GWA studies where only variant association statistics are available. Using the Diabetes Genetics Initiative (DGI) study, we showed that our correction accounts for most of the confounding effects on the most significant SNP score and yields gene scores that are much more accurate than those obtained without correction [31]. Notably, this regression approach and the DGI permutation system can be used to identify and adjust for confounders on other types of SNP to gene scores (e.g. considering best SNP per LD block [25], [33] or the set–based test in PLINK http://pngu.mgh.harvard.edu/~purcell/plink/anal.shtml#set). While in the current work we focus largely on developing a gene set approach following gene score correction, we envisage that the corrected gene *p*-values might be valuable in future gene-centric studies, allowing one to properly weigh specific genes (e.g. small genes) that may otherwise be missed.

### Power of MAGENTA evaluated using simulated and true association data

Using computer simulations, we show that MAGENTA has considerable power (i.e. sensitivity) in detecting multiple modest effects relative to traditional single SNP analysis for a range of parameters. For example, for a gene set size of 100 genes, our method has 50% power of detecting enrichment when ∼10 genes have weak effects (that are equivalent to 1% detection power at single SNP level) versus 10% power of detecting only one of the 10 genes in single SNP analysis. By applying MAGENTA to GWA scan meta-analyses for LDL cholesterol, HDL cholesterol and triglyceride levels, we confirmed the method's ability to pick out relevant biological processes. We note that the nominal MAGENTA *p*-values for these positive controls were not exceedingly low (on the order of 10^−2^ to 10^−6^), emphasizing the limited power of the gene set approach. Our simulations allowed us to provide quantitative estimates of these limitations, and indications of possible limiting factors. For example, we found that power levels increase considerably with gene set size, fraction of causal genes in a gene set, and effect size of associated SNPs, and decrease with total number of causal genes in the genome. Similar trends, as a function of effect size and fraction of causal SNPs, have been shown with other types of GSEA methods that test for enrichment in SNP sets across pathways [32], [33].

### No evidence for a causal role of mitochondrial dysfunction in T2D

Despite a large sample size, comprehensive gene lists, and a calibrated statistical method, we did not find evidence that common variants in proximity to ∼1,000 known nuclear-encoded mitochondrial genes contribute to T2D susceptibility. Similarly, we found no indication of significant associations between variants near these genes and intermediate physiological phenotypes related to T2D. Simulations of MAGENTA performance suggest that if there is a genetic contribution it is small - probably no more than 2–4% of nuclear-encoded mitochondrial genes (∼20–40 genes) harbor common variants of modest effect (e.g. an odds ratio of ∼1.07 for allele frequency of 0.2–0.3 and sample size of 10,000 individuals) on T2D risk. This number may vary to some extent depending on the actual effect sizes and total number of causal genes for the disease (see Figure S6). As of the latest T2D meta-analysis used here (DIAGRAM+), three mitochondrial genes (*IDE*, *C8orf38* (Entrez ID 137682), and *ACADS* (Entrez ID 35)) lie near validated T2D SNPs amongst other genes in the interval [20], but a causal connection for these genes with T2D has not yet been shown.

Although the expression of multiple OXPHOS genes is downregulated in skeletal muscle of patients with diabetes [9], and OXPHOS activity is reduced in diabetic and insulin-resistant individuals, we did not find evidence that OXPHOS genes lie near genetic variants that affect T2D risk or related glycemic traits. This is consistent with a previously reported pathway analysis of one of the T2D GWA studies included in the DIAGRAM+ meta-analysis [27]. Lack of enrichment in the OXPHOS genes suggests that either the changes in expression are an effect and not a cause of diabetes, or that one or few regulators of OXPHOS [35] contain yet undetected rare or common variants, or inherited epigenetic changes associated with T2D or a related phenotype. Since, to date, there is no conclusive evidence for a strong association of any of the 16 known nuclear regulators of mitochondrial genes to T2D, we tested whether several regulators might harbor common variants with modest effect on T2D risk in the population. In our analysis we could not find strong support for this possibility. Our simulations suggest that we would have considerable power to detect enrichment if at least ∼9 OXPHOS genes or at least ∼3 nuclear regulators were modestly associated with T2D or a related trait. While specific genes ranked high among the 16 regulators (but not at gene-wide significance), such as *GABPA* (the GA binding protein transcription factor, alpha subunit) [50] with respect to T2D associations or *SIRT1* (sirtuin, silent mating type information regulation 2 homolog 1; Entrez ID 23411) [51], [52] with respect to fasting insulin levels and measures of insulin resistance and ß-cell function, our statistical tests do not constitute a proof of their involvement in T2D. Future gene-centric approaches using our corrected gene scoring system or others may be used to examine more closely these and similar instances.

We note that while lack of enrichment of associations with T2D and related-traits does not provide support for a causal connection, it does not eliminate the possibility that individual genes could still be found to have a genetic effect and thus be instrumental to T2D predisposition. For example, the absence of enrichment in the OXPHOS genes does not disprove the association to T2D of one of its genes, *C8orf38* (an assembly factor in Complex I, the first complex in the mitochondrial electron transfer chain; Entrez ID 137682) [22], which lies near a validated T2D SNP found in the recent DIAGRAM+ T2D meta-analysis [36], but it does not provide further support for *C8orf38* being causal.

### Limitations of MAGENTA and other GSEA approaches applied to variant association data

Our finding that specific mitochondria-related gene sets functionally implicated in T2D are not enriched for associations could be due to several reasons, of potential relevance also to the study of other diseases: (i) The fraction of causal genes in the given gene set, while considerable, may not be significantly higher than the total fraction of causal genes in the genome (especially relevant to gene permutation analysis); (ii) The causal variants may be spread across a large number of biological processes or there may be allelic heterogeneity in the population, making it hard to detect clustering of associations into pathways; (iii) Causal genes for certain phenotypes may cluster in small pathways, which are more sensitive to individual gene score fluctuations than large pathways; (iv) The relevant pathways or sets of functionally related genes may have not yet been tested; (v) By considering only variants within a given distance around each gene, potential signals from more distant transcriptional regulatory elements, such as enhancers or epigenetic marks, might be missed; future genome-wide maps of regulatory elements may be used to generate a discontinuous and precise map of potential causal regions per gene; and finally (vi) Rare variants were not tested, but when the data are available the MAGENTA framework can be applied to this class of variants.

### General applications of MAGENTA and other GSEA approaches to GWA studies

Certain common diseases and traits may be more amenable to GSEA approaches than others, depending on their genetic architecture. In addition to identifying new biological pathways or processes associated with disease risk or trait variation, GSEA methods, such as MAGENTA, may provide predictions for new disease or trait genes of modest effects (top ranked gene scores in enriched gene sets). Such joint analysis of SNPs (or other types of variants) at the gene and gene set levels should be most useful for detecting associations in a narrow range of nominal significance levels (between noise levels, e.g. *p*<0.1, and SNP replication cutoff, e.g. *p*>∼0.0001), a range that has been shown to contain associations of small effect in polygenic disorders [53]. The GSEA approach may also help prioritize potential causal genes in validated association regions that contain multiple genes.

Our method which explicitly accounts for important confounders on the association scores of genes (e.g. gene size) and gene sets (e.g. positional effects of genes in a gene set) in the absence of genotype data, and that provides upper-bound estimates of number of associations per gene set, should provide accurate tests of gene sets of interest, especially for analyzing large GWA scan meta-analyses. MAGENTA can also be applied to sets of genetic loci other than genes, such as linkage disequilibrium blocks. More generally, such GSEA approaches may be valuable for gene and pathway analysis of other types of genetic studies that deal with multiple measurements per gene, such as exon resequencing in case-control studies.

## Materials and Methods

### Ethics statement

The study constitutes a secondary analysis of genetic data derived from de-identified samples, and thus has an IRB exemption.

### GWA studies and meta-analyses analyzed

Two type 2 diabetes (T2D) GWA studies were analyzed in this work. The first is the Diabetes Genetics Initiative (DGI) GWA study, used for method development purposes. 381,099 genotyped SNPs were analyzed using only the population-based individuals, that consist of 1,022 diabetic patients and 1,075 matched control individuals (a total of 2,097 individuals) [17]. The second study is the most recent T2D GWA meta-analysis (DIAGRAM+) [36], used to test the mitochondrial-diabetes hypothesis with MAGENTA. The meta-analysis was performed across eight GWA studies, with a total of 8,130 diabetic patients and 38,987 non-diabetic controls (47,117 individuals total, effective sample size n = 22,044), and 2,255,856 genotyped and imputed autosomal SNPs.

The GWA study meta-analyses of seven diabetes-related glycemic traits analyzed in this work were part of the Meta-Analyses of Glucose and Insulin-related traits Consortium (MAGIC) [37], [38] (Soranzo N. *et al.*, unpublished data). These seven traits include fasting glucose concentrations, fasting insulin concentrations, 2-hour glucose and 2-hour insulin concentrations after an oral glucose tolerance test, indices of β-cell function (HOMA-B) and of insulin resistance (HOMA-IR), calculated from fasting glucose and insulin measures using homeostasis model assessment [49], and HbA_1C_ (glycated hemoglobin) levels. The meta-analyses for fasting glucose, fasting insulin, HOMA-B and HOMA-IR were performed on 20 or 21 GWA studies with a total of 36,466 to 46,186 non-diabetic individuals [37], [38], the meta-analyses for 2-hour glucose and 2-hour insulin were performed across 9 studies and a total of 15,234 individuals [37], [38], and the meta-analysis for HbA_1C_ was performed across 23 cohorts with a total of 35,920 non-diabetic individuals (Soranzo N. *et al.*, unpublished data). The total number of genotyped and imputed autosomal SNPs analyzed in these seven meta-analyses varied between 2,323,569 and 2,748,910 SNPs.

To test the performance of MAGENTA on traits whose underlying biology has been well studied, we analyzed three GWA study meta-analyses of low-density lipoprotein (LDL) cholesterol, high-density lipoprotein (HDL) cholesterol and triglyceride blood levels [34]. All three meta-analyses were performed with 19,840 individuals from seven GWA studies, on 2,552,754, 2,552,580 and 2,552,773 genotyped and imputed SNPs for the LDL cholesterol, HDL cholesterol and triglyceride meta-analyses, respectively.

The association tests of all the aforementioned GWA studies were performed at the single SNP level, assuming an additive allelic model. The individuals in all GWA studies are of European descent.

### Meta-Analysis Gene-set Enrichment of variaNT Associations (MAGENTA)

#### Step 1: Mapping SNPs onto genes

A list of 26,914 human gene transcripts was downloaded from the UCSC Genome Browser (http://genome.ucsc.edu/) in RefFlat format based on the human March 2006 (hg18) assembly. In the current study, 18,434 unique genes were used (17,680 on autosomes, and 754 on sex chromosomes), after filtering out genes with two or more transcripts that lie more than 1Mb apart on the same chromosome or that lie on separate chromosomes. All the genotyped or imputed SNPs that lie within an added physical distance upstream or downstream to a gene's most extreme transcript start and end sites of all its known splicing isoforms (intron and other non-coding sequences included) were assigned to each of the 18,434 genes. For gene set enrichment analysis of T2D, glycemic traits, and lipid and lipoprotein traits we used 110 kb upstream to the gene's most extreme transcript start site and 40 kb downstream to the gene's most extreme transcript end site. These boundaries were chosen as they represent the 99^th^ percentile of the distances of *cis*-eQTLs from their adjacent gene's transcript start and end sites. This is according to a comprehensive genome-wide analysis of putative functional regulatory elements (*cis*-eQTLs) using expression data from human lymphoblastoid cell lines [54]. These boundaries were chosen in attempt to capture association signals from proximal regulatory regions, in addition to the coding region. For the analysis of the DGI GWA study and the DGI permutations (used for method development purposes), ±50 kb was used, as these analyses were done before the Veyrieras *et al.* publication [54]. In the future, when transcriptional elements are comprehensively characterized for all genes in the genome, a discontinuous and more precise map of regulatory regions for each gene could be used for assigning SNPs to genes.

#### Steps 2 and 3: Scoring genes based on SNP association scores and correcting for confounders

For each gene *g* in the genome we calculated a score, 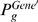 that is the probability that the gene is associated with a given disease or trait. In computing this score we corrected for the confounding effects of physical and genetic properties of genes on the gene *p*-value.

#### Step 2

The scoring metric used here is as follows: For each gene *g*, the minimum GWA *p*-value of all SNPs with index *i* that fall within the extended gene boundaries (see Step 1) is chosen, 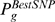:
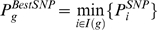
(1)where *I(g)* is the set of indeces of SNPs whose chromosome positions fall between the extended gene boundaries. 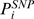 is the association *p*-value for SNP *i* calculated in a GWA study or meta-analysis (see GWA studies and meta-analyses section). A z-score 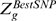 is then computed based on 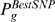 for each gene *g*, using a mean of 0 and standard deviation of 1, assuming a normal distribution. 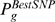 should be most powerful for genes that contain one major target region or haplotype with potential causal mutations in or around their coding sequence. Other SNP to gene scoring metrics can be used here.

#### Step 3

To correct for confounding effects on 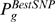 we regressed out the effect of several potential confounders from 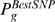, using step-wise multiple linear regression analysis [55]. The method begins by regressing out the effect of a variable with high correlation with the gene score; it then adds the next significant variable, and evaluates whether the added variable should be kept and whether any existing variables should be eliminated from the regression model. The latter step is repeated until all variables are considered. A variable was added at *p*<0.05 and removed at *p*>0.1. The step-wise nature of this method should account for correlations between the variables. We initially tested this model using 1,000 DGI GWA permutations and six gene properties as potential confounders (predictor variables). In this case, step-wise multivariate linear regression was applied to 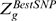 using the full list of genes, and the coefficients *α*, *β*, *δ*, *γ*, *η*, and 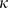 were estimated such that for every gene *g* one can calculate:
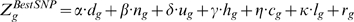
(2)where 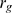 is the residual of the association score for gene *g* that cannot be explained by the effects of the predictor variables considered. After the regression a corrected gene z-score, 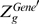 can be written as follows:
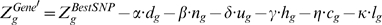
(3)A corrected gene *p*-value 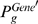 is calculated from 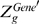 assuming a normal distribution and a mean of 0 and standard deviation of 1 (reasonable approximation but not perfect, in particular for the less significant values of 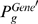; see Figure S8). Of all six gene properties tested, only the genetic distance, 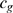, was not significant (p>0.05) in most of the DGI permutations subjected to the regression analysis (Table S3), and hence 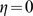 was used for all analyses in this paper. Similar GSEA results were obtained for all gene sets and traits analyzed in this paper when only the first four variables listed in Table 1 (significant in almost all 1,000 DGI permutations tested; see Table S3) were used for the regression-based correction of gene scores (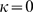 and 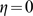) (data not shown).

This step-wise linear regression approach can be used to adjust for confounders on other types of variant to gene scoring metrics, and an appropriate set of potential confounders can be identified using the DGI permutation system described below.

#### Comparison to analytical gene score correction methods

The regression-based method was compared to Sidak's combination test, also known as Sidak's correction [26], and to a modified version of Sidak's correction [40]. The corrected gene *p*-value, 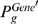 based on Sidak's correction is defined as follows for gene *g*:
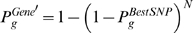
(4)where 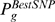, defined in eq. 1, is the most significant SNP *p*-value for gene *g*, and N is the total number of SNPs with available association statistics for gene *g*. A modification of eq. 4 proposed in [40] uses (*N*+1)/2 as the exponent to adjust for linkage disequilibrium between regional SNPs, assuming ∼50% of SNPs in a given genomic region are in tight linkage disequilibrium:
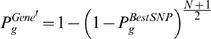
(5)


#### Step 4: Gene set enrichment analysis of genome-wide association data

To test for over-representation of genes with modest genetic effects on a complex disease or trait in predefined sets of genes, we developed a gene set enrichment analysis (GSEA) algorithm that is applied to gene association *p*-values adjusted for confounding effects. This algorithm does not require the genotypes of individuals in the association scans in order to estimate gene set enrichment significance. Our GSEA test was inspired by the original GSEA algorithm applied to expression data [9], [24], more recently modified for SNP association data [23], [25], [28], [32], but uses a different statistical test. The null hypothesis is that the gene association score ranks of all genes with index *g* that belong to a given gene set *gs* are randomly distributed. The alternative hypothesis is that there is an over-representation in gene set *gs* of gene score ranks above a given rank cutoff compared to multiple random gene sets of identical size that were randomly sampled from all genes in the genome.

The specific steps of the GSEA statistical test employed here are as follows: (i) Corrected gene association *p*-values were calculated for all genes in the genome, based on a given GWA study or meta-analysis. In this study, we used the corrected gene *p*-value, 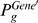 as it can be computed for studies where individuals' genotypes are not available. If genotype data are available, the gene score 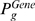 can also be computed (see above for 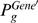 definition and section below for 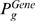 definition). (ii) Several types of genes were removed from gene sets. Genes with no SNPs in their extended gene boundaries were not included in the analysis. In addition, for each subset of genes in a given gene set that were assigned the same most significant SNP, all genes but one were removed from the analysis; the gene with the most significant gene score was retained. This was done to eliminate potential inflation of gene set enrichment significance due to two or more genes in a gene set that are physically proximal along the chromosome and hence may capture the same association signal (assuming one causal gene per associated locus). This yielded an effective number and set of genes that was used for the next steps of the GSEA test. (iii) For each gene set *gs* the fraction of genes with 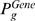
<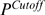 was recorded (denoted here as the ‘leading edge fraction’), where 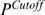 is a predetermined gene *p*-value cutoff, defined as a given percentile of all gene *p*-values in the genome. 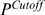 is specific for a given GWA study or meta-analysis. In this study, we used 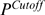 = 95^th^ percentile of 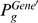 for all genes *g* in the genome, as it gave the optimal power of five cutoffs tested (99^th^, 95^th^, 90^th^, 75^th^, and 50^th^ percentile of all gene *p*-values) with power simulations (see Figure S5 and Simulations section below). (iv) Finally, a nominal GSEA *p*-value, 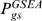 was calculated for each gene set *gs*, defined as the fraction of randomly sampled gene sets of identical set size, whose leading edge fraction is equivalent to or larger than the observed leading edge fraction of gene set *gs*. The null distribution of leading edge fractions was generated for each gene set *gs* by randomly sampling 10,000 gene sets from the genome (or more when 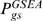
<10^−4^) that are of identical set size to the effective size of gene set *gs* (after adjusting for physical clustering in the genome of subsets of genes in each randomly sampled gene set separately, as described above). Genes in gene set *gs* were not excluded from the random sampling procedure. (v) To correct for multiple hypothesis testing, Bonferroni correction was used (i.e. significance cutoff *p* = 0.05 divided by the number of hypotheses tested). This may be too stringent when a large number of gene sets is tested due to overlap of genes between the different gene sets.

To test the robustness of our GSEA results for the mitochondria-related gene sets, we applied an alternative GSEA statistical test, based on a one-tailed Mann-Whitney rank-sum test (Table S13). First, for each gene set *gs* we calculated a one-tailed rank-sum *p*-value that tests the alternative hypothesis that 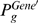 ranks for all genes in gene set *gs* are skewed towards high ranks compared to the gene score ranks of the rest of the genes in the genome. Second, a similar one-tailed rank sum *p*-value was calculated for 10,000 random gene sets of identical size that were randomly sampled from the genome and adjusted for chromosome clustering of subsets of genes in the gene set. Finally, a rank-sum based GSEA *p*-value, 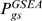 was computed for gene set *gs* as the fraction of randomly sampled gene sets whose rank-sum *p*-value was equivalent to or more significant than the rank-sum *p*-value of the tested gene set *gs*.

### Identifying confounders on gene association scores

The potential confounding effects of six gene properties on the most significant SNP *p*-value, 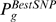 for all genes *g* were examined using 1,000 DGI study permutations, described below. The gene features tested include: (1) Physical gene size for gene *g*, *d_g_*, defined as the distance in kilobase (kb) units between the most extreme transcript start and end sites of all isoforms of a given gene (including introns), plus an added distance. For the extended boundaries of −110kb/+40kb used for the mitochondrial and lipid analyses, 150 kb were added, and for the ±50 kb boundaries used for method development purposes, 100 kb were added; (2) Number of genotyped and imputed (if available) SNPs per kb for each gene *g*, *n_g_*; (3) Estimated number of independent SNPs (that are in approximate linkage equilibrium with each other) per kb for each gene *g*, *u_g_*. This was calculated using the *–indep* option in PLINK that prunes SNPs based on the variance inflation factor, VIF (http://pngu.mgh.harvard.edu/~purcell/plink/summary.shtml#prune; default parameters were used). The genotypes of the CEU population from HapMap version 19 were used, since the GWA samples analyzed in this work are of European descendent. This yielded 310,399 independent autosomal SNPs; (4) Number of recombination hotspots spanning gene *g* per kb, *h_g_*. Recombination hotspot positions were taken from [56]; (5) Genetic distance of each gene *g*, *c_g_* in centi-Morgan (cM) per kb units calculated based on a fine-scale map of recombination rates [56]; and (6) Linkage disequilibrium units (LDU) per kb for each gene *g*, *l_g_*, calculated based on an LDU map downloaded from http://cedar.genetics.soton.ac.uk/pub/PROGRAMS/LDMAP [57]. All variables were calculated based on the extended gene boundaries. All variables but gene size, *d_g_* were transformed to ‘per kilobase’ units: variables *n_g_*, *u_g_*, and *h_g_*, were divided by *d_g_*, and variables *c_g_* and *l_g_* were divided by the physical distance between the most extreme genetic markers within the gene boundaries for which genetic distance or LDU data were available. All six variables showed a significant correlation with 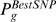 for all genes *g*, using 1,000 DGI study permutations, both before and after normalization to gene region size.

### Permutation analysis of Diabetes Genetics Initiative GWA study

We used the Diabetes Genetics Initiative (DGI) GWA study [17] as a test case for developing MAGENTA, as we had access to genotypes of all individuals in this study (as opposed to the GWA meta-analyses analyzed in this paper where we do not have access to genotype data). The analysis was done only on the population-based samples of the DGI study - 1,022 cases and 1,075 controls that were matched for age, gender, body mass index and region of origin. Specifically, the T2D case/control labels were randomly permuted 1,000 times between individuals from the same collection center and the same gender. A genome-wide association test (logistic regression) that assumes an additive allelic model (1 degree of freedom) followed by a genomic control (adjustment for lambda larger than 1) was then applied to each of the 381,099 genotyped SNPs across the 1,000 permutations, resulting in an association *p*-value, 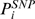 for each SNP *i* and each permutation. 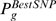 was calculated for all genes in the genome, across the 1,000 DGI permutations. A gene *p*-value adjusted for confounding effects with permutation analysis, 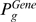 was then calculated for each gene *g* in the genome. 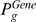 is defined as the fraction of permutations whose 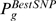 is equal to or lower (more significant) than the observed DGI 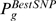. We performed an additional 10,000 case/control permutations for SNPs within ±50 kb around genes with 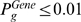 to increase resolution. Genes with 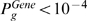 were assigned 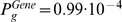.

The gene score vectors before correction (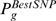) calculated for the 1,000 DGI permuted data sets were used to quantify the correlation between six gene properties of potential confounding effects on 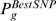 and 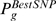 (Table 1). The permutations were also used to evaluate which of the correlated gene properties had a significant confounding effect on 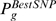 based on a step-wise multivariate linear regression model (Table S3). The resulting significant confounders were used in all gene set analyses presented in this study. To assess the performance of our regression-based correction of confounders on 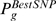, Sidak's correction and a modified Sidak's correction, we compared the corrected gene *p*-values, 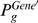 to the corresponding gene *p*-values corrected with permutation analysis, 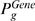 for all genes *g*, using the actual DGI study. Permutation analysis was used as the gold standard for adjusting for confounders on SNP to gene scores as it generates gene-specific null distributions while maintaining the physical and genetic structure of SNPs across gene regions. This enables correcting for all possible confounding effects on gene association scores without requiring *a priori* knowledge of the confounders. The performance of our regression-based correction, Sidak's correction and a modified Sidak's correction were evaluated by comparing the Pearson's correlation coefficient between 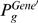 and 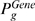 to the correlation coefficient between the unadjusted gene score, 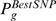 and 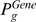 for all genes *g* in the genome.

The permuted 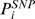 for all SNPs *i* were also used for power simulations described in the next section.

### Simulations used to estimate sensitivity and specificity of MAGENTA

We developed a simulation framework to evaluate the power of MAGENTA to identify enrichment of multiple associations for which we have low detection power with single SNP analysis. SNPs with a small effect size were randomly spiked into varying numbers of genes (referred to as causal genes) in pre-specified gene sets (one SNP per gene), and into genes outside the gene set, maintaining the total number of causal genes in the genome. The simulations were performed on a background of randomized SNP association *p*-values, 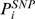 for all SNPs *i* in the genome, generated with phenotype permutations of the DGI study (see section above). For each set of parameters tested, 1,000 simulation runs were performed. In each simulation run, the genes representing a simulated gene set of a given size were randomly chosen from the genome, and the various fractions of genes assigned a SNP of small effect size were also randomly chosen from all genes in the gene set. The remaining number of causal SNPs was randomly assigned to genes outside the gene set. The small effects were randomly assigned to SNPs within the ±50kb extended gene boundaries (see above for boundary definition). To eliminate artifacts that could arise from using one specific vector of permuted 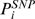, each simulation run was done on a different GWA study permutation background that was randomly chosen from 1,000 different DGI phenotype permutations. For each of the 1,000 simulation runs, gene *p*-values corrected with multivariate regression analysis (see above), 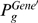 were calculated for all genes *g* in the genome. The GSEA algorithm in MAGENTA was then applied to the simulated gene set with a given fraction of causal genes of weak effect. Finally, GSEA power (i.e. sensitivity) was estimated as the fraction of 1,000 spike-in simulations whose gene set enrichment *p*-value, 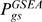 exceeded a given significance level (in this study 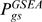
≤0.01, a suitable cutoff for the few hypotheses tested in the mitochondrial gene set analysis). The power does not decrease significantly when a more stringent cutoff is used: 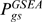
≤0.001 (Figure S9).

The parameters used in the simulations are: (i) Gene set size of 25, 100 or 1000 genes; (ii) Fraction of genes in a gene set that got assigned a SNP with a modest effect size: 0 (negative control), 1%, 5%, and 10%, 20%, 30%, 40%, 50% and 60%; (iii) The small effect size of each spiked-in SNP was estimated by randomly sampling from a noncentral chi-square distribution with one degree of freedom (assuming an additive allelic test). The non-centrality parameters (NCP) used were: NCP = 0 for estimating specificity or false positive rate of our GSEA method, NCP = 2.5 for a very weak effect size (equivalent to 1% power of detection at p≤1e-4 using single SNP analysis; e.g. odds ratio of 1.03–1.04 for an allele frequency of 0.2–0.3 and sample size of 10,000 individuals [41]; Figure S6A), and NCP = 10 for a modest effect size (equivalent to 1% power of detection at genome-wide significance (p≤5e-8) using single SNP analysis; e.g. odds ratio of 1.07 for an allele frequency of 0.2–0.3 and sample size of 10,000 individuals [41]; Figure 3); and (iv) A total of 100 (Figure 3) or 500 (Figure S6B) causal genes in the genome. The chi-square test statistic was then converted to a z-score by taking the square root of the chi-square test statistic. Parameters were chosen in attempt to reflect what we know about the genetic architecture of complex diseases and traits.

This simulation framework was also used to choose an optimal gene score enrichment cutoff, 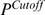 for our GSEA algorithm. Five cutoffs were tested: 99^th^, 95^th^, 90^th^, 75^th^, and 50^th^ percentile of all gene *p*-values for two effect sizes: NCP = 2.5 and NCP = 10, assuming a total of 100 causal genes in the genome. A 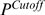 equivalent to the 95^th^ percentile of 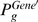 for all genes *g* in the genome yielded the optimal power, when considering power plots for both effect sizes (Figure S5). The 75^th^ percentile cutoff performed a bit better than the 95^th^ percentile cutoff for very weak effects (NCP = 2.5; Figure S5B), especially when assuming a total of 500 causal genes (data not shown). Hence, the 75^th^ percentile cutoff could be used for diseases or traits that are highly polygenic with many associations of weak effects.

### Gene sets analyzed with MAGENTA

#### Mitochondria-related gene sets

Of the 1,012 unique human mitochondrial genes described in MitoCarta [22], we analyzed 966 autosomal mitochondrial genes. This number was obtained after removing 13 genes encoded by the mitochondrial DNA and 31 mitochondrial genes that lie on the X and Y chromosomes, as they were not analyzed in the GWA studies and meta-analyses used in this work. Two autosomal genes were removed, as they were absent from the human gene list used for our analyses. For the DIAGRAM+ T2D meta-analysis, the effective gene set size of all mitochondrial genes was 885 genes, as 11 genes did not have any genotyped or imputed SNPs within their extended gene boundaries (110 kb upstream and 40 kb downstream to the most extreme transcript boundaries) and 70 genes were removed following physical proximity adjustment described in the GSEA section. There are 110,060 unique SNPs that fall within the gene regions of the 966 nuclear-encoded mitochondrial genes, based on the DIAGRAM+ meta-analysis (4.9% of all SNPs).

A list of 91 oxidative phosphorylation (OXPHOS) genes out of the 966 autosomal, mitochondrial genes was manually curated (marked in Table S12). This list does not include 12 OXPHOS genes encoded by the mitochondrial DNA and 3 genes on chromosome X. There are 9,693 SNPs that fall within the gene regions of the 91 OXPHOS genes based on the DIAGRAM+ meta-analysis (0.4% of all SNPs).

A set of 16 known nuclear transcriptional regulators of mitochondrial functions was assembled based on the literature [35], [44]–[48] (Table S11). All mitochondria regulators had SNPs in their extended gene boundaries using the DIAGRAM+ meta-analysis.

#### Lipid- and lipoprotein-related gene sets

We tested 15 biological processes related to lipid, fatty acid and steroid metabolism defined by the PANTHER classification method (http://www.pantherdb.org/) [42], and 36 gene sets related to lipid, lipoprotein and fatty acid metabolism defined by Gene Ontology [43], which include 7 molecular functions and 29 biological processes. The Gene Ontology gene sets were taken from the Molecular Signatures Database (MsigDB, http://www.broad.mit.edu/gsea/msigdb/collections.jsp).

In this paper we analyzed gene sets with an initial gene set size of 10 genes or more.

### Software

MAGENTA is freely available for use at http://broadinstitute.org/mpg/magenta.

## Supporting Information

Figure S1 Cumulative distribution of mitochondrial and non-mitochondrial gene scores before and after adjustment for confounders. The cumulative *p*-value distributions are plotted for the most significant SNP T2D association *p*-value within each gene's extended boundaries (A) before and (B) after adjustment for gene score confounders. The distributions are plotted for 966 autosomal mitochondrial genes (red line), the oxidative phosphorylation (OXPHOS) subset (green line), and the rest of the genes in the genome that have at least one SNP in their region (non-mitochondrial genes; blue line) (see Materials and Methods for details). The correction presented in panel B is following a step-wise multivariate linear regression analysis of the most significant SNP *p*-value against the first five gene properties listed in Table 1. The x-axis is on a log_10_ scale in both panels.
(0.25 MB PDF)

Figure S2Distribution of T2D gene *p*-values for small, large and all genes before and after correction for confounders. (A) The distribution of the mean 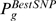 (best SNP association *p*-value per gene *g*) calculated across 1,000 phenotype permutations of the Diabetes Genetics Initiative (DGI) GWA study is shown for all genes in genome (blue line), only large genes (≥100 kilobase (kb); red line), and only small genes (≤10 kb; green line). Large genes tended to receive on average a more significant gene score (lower *p*-values) than all genes in the permuted datasets, and small genes tended to receive on average a less significant gene score (higher *p*-values) than all genes. (B–D) The distribution of gene association *p*-values is shown for the actual DGI study for all gene sizes (blue line), large genes (red line) and small genes (green line) (B) before correcting for confounders (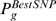), and after correcting for confounders on 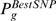, such as gene size, using either (C) phenotype permutation analysis (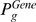) or (D) step-wise multivariate linear regression analysis (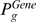). The regression-based correction transforms the gene *p*-values to a distribution that is close to uniform and removes the confounding effect of gene size, similar to the permutation-based correction, which corrects for all confounding effects without *a priori* knowledge of them. The regression correction seems to slightly over-correct the gene *p*-values of large genes (red line in D) in the high *p*-value end of the distribution (p>0.8). A bin of 0.01 was used for all four plots.(0.66 MB PDF)

Figure S3A comparison of the performance of several gene association score correction methods. T2D gene association *p*-values were plotted (A) before gene score adjustment (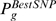) and after correction for potential SNP-to-gene score confounders (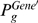), as a function of gene *p*-values corrected with phenotype permutation analysis (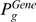). The correction methods tested: (B) step-wise multivariate linear regression analysis, (C) Sidak's correction (eq. 4 in Materials and Methods) and (D) a modified version of Sidak's correction (eq. 5 in Materials and Methods; Saccone SF *et al.*, Human Molecular Genetics 16(1): 36–49, 2007). The Diabetes Genetics Initiative (DGI) study was used for the analysis, as we had access to genotype data in this study. The unadjusted gene *p*-value, 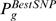 is the association *p*-value of the best regional SNP for gene *g* (y-axis in A). Phenotype permutation analysis was used as the gold standard to test goodness of gene score correction as it corrects for all confounders without requiring *a priori* knowledge of the confounders (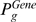). The Pearson's correlation coefficient (calculated between *p*-value vectors before log transformation) increased significantly following each of the three correction methods (from r = 0.69 to r = 0.94–0.97), but the Sidak's correction (C) did not perform as well, as it tends to overcorrect (most of the dots fall below the diagonal, the red line). The spread around the diagonal also decreased for all three correction methods. While the modified Sidak's correction (D) performs a bit better than the regression-based correction (B) in the DGI study, Figure S4 shows that its performance varies between GWA studies of different SNP densities. The correction for linkage between SNPs in the modified Sidak's correction equation may need to be adjusted for different GWA studies or meta-analyses with different SNP densities (see Figure S4 for details). The minimum 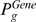 is 10^−4^ as the *p*-values were calculated based on 1,000 permutations for genes with 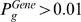 and 10,000 permutations for genes with 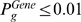. Gene scores are plotted on a −log_10_(*p*-value) scale.(0.42 MB PDF)

Figure S4Distribution of gene association *p*-values for different T2D GWA studies and gene score correction methods. Presented here are the distributions of the best SNP per gene *p*-values for all genes after adjustment for confounders (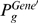), using two different correction methods: (A–B) a step-wise multivariate linear regression analysis that regresses out physical and linkage-related confounders from the most significant SNP association z-score, and (C–D) a modification of the Sidak's correction equation that uses an exponent of about half the number of SNPs per gene to adjust for linkage disequilibrium between SNPs in a given chromosomal region (eq. 5 in Materials and Methods). A bin of 0.01 was used in all four panels. The distribution of 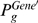 following regression analysis is similar for the DGI study (A) that contains ∼3.8e5 genotyped SNPs (on average 1 SNP/8kb) and the DIAGRAM+ T2D meta-analysis (B) that contains ∼2.3e6 genotyped or imputed SNPs (on average 1 SNP/1.3kb). The regression-corrected 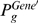 distributions in both studies are close to uniform, aside for an excess in the low *p*-value tail and a slight deviation from uniformity in the high p-value tail. Panels A and B show that the regression correction, which explicitly takes into account linkage disequilibrium properties between SNPs in a gene-specific manner, is adjustable to studies with different SNP densities and linkage properties. The distribution of 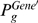 following the modified Sidak's method is also close to uniform in the DGI study (C). However, in the DIAGRAM+ meta-analysis, which contains about 6-fold more SNPs than the DGI study, the modified Sidak's correction distribution is largely skewed towards high values of 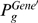 (D) (∼11.4% of genes with 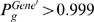 where only 0.1% is expected, and ∼19.4% of genes with 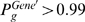 where only 1% is expected). This difference in performance of the modified Sidak correction between the DGI and DIAGRAM+ studies may be due to differences in SNP density, which may affect the effective fraction of SNPs that are in tight linkage disequilibrium in different regions along the genome. Hence, the exponent in Sidak's equation (eq. 5 in Materials and Methods) might need to be adjusted for different studies.(0.42 MB PDF)

Figure S5 Using simulations to find an optimal gene set enrichment cutoff. The power of detecting gene set enrichment of multiple modest (A) or weak (B) effects was estimated with simulations as a function of fractions of causal genes in a gene set of 100 genes, for five different enrichment cutoffs: 99^th^ percentile (black line), 95^th^ percentile (dark blue line), 90^th^ percentile (green line), 75^th^ percentile (red line), or 50^th^ percentile (cyan line) of all corrected gene *p*-values (with regression analysis). The modest effect size in (A) represents 1% power of detecting an association at genome-wide significance (*p*-value<5e-8) using single SNP analysis, and the weak effect size in (B) represents 1% power of detecting an association at *p*-value<1e-4 using single SNP analysis. A total of 100 causal genes in the genome was assumed here. These plots show that power of MAGENTA to detect enrichment of multiple modest effects is fairly robust to the enrichment cutoff used. Overall, the 95^th^ percentile cutoff performed the best. While the 99^th^ and 95^th^ percentile cutoffs performed similarly in detecting enrichment of multiple modest effects (A), the 95^th^ percentile cutoff performed significantly better in detecting enrichment of many weak effects (B). Note the log_10_ scale of the x-axis in both panels.
(0.41 MB PDF)

Figure S6Power of MAGENTA as a function of effect size and total number of causal genes in the genome. (A) Power of detecting gene set enrichment of multiple modest associations increases with effect size. Using computer simulations we assessed the power of MAGENTA to detect enrichment of multiple SNPs of modest effect spiked into various fractions of genes (causal genes) in a gene set size of 100 genes (one SNP per gene). Two different effect sizes were tested: (i) the modest effect (solid line) represents 1% power of detecting a SNP association at genome-wide significance (*p*-value<5e-8) using single SNP analysis, and the weak effect (dashed line) represents 1% power of detecting an association at *p*-value<1e-4 using single SNP analysis (details in Materials and Methods). A similar trend was obtained for a gene set size of 25 and 1,000 genes (data not shown). The false positive rate for the parameters used here was between 0.4–1.7%. (B) Power of detecting gene set enrichment of modest associations decreases as the total number of causal genes in the genome increases. Power was estimated assuming a total of 100 (solid line) or 500 (dashed line) causal genes in the genome. For both panels a gene set was considered significant at a GSEA *p*-value cutoff of 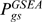
<0.01. Note the logarithmic scale of the *x*-axis for both plots.(0.28 MB PDF)

Figure S7Quantile-quantile plots of T2D gene association *p*-values for mitochondria-related gene sets. The T2D gene association *p*-values adjusted for confounding effects using step-wise multivariate linear regression analysis, 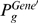 (see Materials and Methods) were plotted for (A) 16 nuclear regulators of mitochondrial genes, (B) 91 oxidative phosphorylation genes, and (C) all known nuclear-encoded autosomal mitochondrial genes with at least one SNP in their region (955 genes), as a function of their corresponding null distributions of 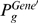 assuming a uniform distribution. Three mitochondrial genes that lie near validated T2D SNPs, as of the most recent DIAGRAM+ T2D meta-analysis are labeled in red (*IDE*, *C8orf38*, and *ACADS*). The red line marks the diagonal, and the dashed lines represent 5% and 95% confidence intervals estimated based on 1,000 randomly sampled gene sets from the genome of identical set size to the given gene set. All gene *p*-values lie within the non-parametric 95% confidence intervals. Similar results were obtained when the observed gene *p*-values were plotted against an expected distribution that was adjusted according to a non-parametric null distribution, generated based on 1,000 randomly sampled gene sets from the genome of identical size to that of the tested gene set (data not shown). 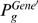 is plotted on a −log_10_(*p*-value) scale. Note the x and y-axes of the three plots are not on the same scale.(0.31 MB PDF)

Figure S8Distribution of T2D gene association *p*-values following correction for confounders. (A) The distribution of the unadjusted best SNP association *p*-value, 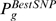 for all genes *g* in the genome is shown using the Diabetes Genetics Initiative (DGI) GWA study. Since the most significant SNP in a gene region was chosen for each gene the distribution is skewed towards low *p*-values. (B) The distribution of all DGI gene *p*-values following correction for confounders using phenotype permutation analysis (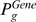) demonstrates how the correction transforms 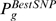 into a uniform distribution. An excess of significant genes is seen at 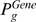
<0.001. (C) The distribution of all DGI gene *p*-values following correction using step-wise multivariate linear regression analysis (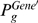) on the first four confounders listed in Table 1 is close to uniform, similar to 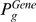 (in panel B). A slight deviation from uniformity is seen for 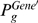 at the less significant end of the *p*-values. An excess of significant genes is also observed at 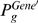
<0.001. (D) The distribution of all gene *p*-values computed for the DIAGRAM+ T2D GWA meta-analysis, following step-wise linear regression of 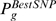 against the first five confounders listed in Table 1 (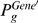) transforms the skewed 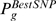 distribution to a reasonably uniform one, similar to the DGI study. An excess of significant genes is also observed at 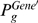
<0.001. A bin of 0.001 was used for all four plots.(0.53 MB PDF)

Figure S9Power of MAGENTA as a function of gene set enrichment significance threshold. We compared the effect of two *p*-value thresholds used to call a gene set significantly enriched in a given simulation run, on the power of MAGENTA to detect gene set enrichment. The two cutoffs tested were: 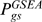
<0.01 (solid line) and 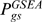
<0.001 (dashed line). 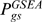 is the nominal enrichment *p*-value for gene set *gs*. Power is plotted as a function of the fraction of causal genes that were randomly assigned a SNP with a modest effect size equivalent to 1% power of detecting an association at genome-wide significance (*p*-value<5e-8) using single SNP analysis. Two gene set sizes were examined: 100 genes (squares) and 1,000 genes (circles). Power appears to decrease only slightly with a more stringent GSEA *p*-value threshold. Note the x-axis is on a log_10_ scale.(0.24 MB PDF)

Table S1 Average gene size of nuclear-encoded mitochondrial genes compared to non-mitochondrial genes.
Mitochondrial genes refer to nuclear-encoded mitochondrial genes on autosomal chromosomes taken from the MitoCarta compendium (Pagliarini DJ, et al. (2008), Cell 134: 112–123). OXPHOS genes refer to the oxidative phosphorylation gene subset. The calculations are based on the March 2006 (hg18) assembly of all human genes. bp, base pairs.
(0.04 MB PDF)

Table S2Correlation between T2D gene association scores, computed from DIAGRAM+ meta-analysis, and six potential confounders. Pearson's correlation coefficient (r) was calculated between the unadjusted and adjusted best SNP per gene z-scores, 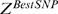 and 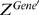, respectively, and six physical and linkage-related gene properties, using the DIAGRAM+ T2D GWA study meta-analysis. Aside for gene size, all gene properties were divided by the size of the gene plus its extended physical boundaries (150 kb was added to the most extreme transcript size for each gene, as the −110kb/+40kb extended gene boundary was used). 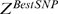 is a vector of the uncorrected gene z-scores for all genes in genome, and 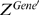 is a vector of corrected gene z-scores for all genes, using step-wise multivariate linear regression analysis. All correlations between 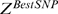 and the six variables were statistically significant (*p*<1e-28). ^†^These gene properties were significant at *p*<0.05 under a step-wise multivariate linear regression model that regresses 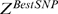 against all six gene properties (see Table S4 for regression model parameters and *p*-values).(0.20 MB PDF)

Table S3Using GWA permutations to identify significant confounders on gene scores under a multivariate regression model. For each of the 1,000 Diabetes Genetic Initiative (DGI) GWA study permutations (described in Materials and Methods) we applied step-wise multivariate linear regression analysis to the most significant SNP per gene *p*-value, 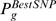 for all genes *g*, against the six gene properties listed in the table. We used the fraction of permuted GWA studies for which a given gene property was included in the regression model (at *p*<0.05) to assess the significance of each gene property as a confounder on 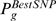. GWA study permutations are not expected to contain true associations, and hence any correlation between 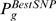 and a gene property in a permuted dataset should be due solely to artificial or confounding effects. All gene properties aside for gene size were divided by the size of the gene and its extended physical boundaries (the gene boundaries used in this analysis were ±50kb around the gene's most extreme transcript boundaries). For all gene set analyses performed in this paper, we chose to include the gene properties that were significant under the regression model in at least ∼50% of permutations for gene score adjustment, and therefore we used the first five properties listed in this table. We obtained very similar GSEA results for all gene sets and GWA studies tested in this paper, when only the first four properties listed in the table, that were significant in almost all permutations tested, were used (data not shown).(0.13 MB PDF)

Table S4Parameters of step-wise multivariate linear regression models of T2D gene scores against gene score confounders. The parameters of a step-wise multivariate linear regression model of the best SNP *p*-value, 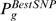 (the response variable) for all genes *g*, on five gene properties (potential gene score confounders; the predictor variables) are listed here for the Diabetes Genetics Initiative (DGI) GWA study and the DIAGRAM+ T2D GWA meta-analysis. The confounding variables imputed into the regression model were those variables that were significant under the regression model in more than about half of the 1,000 DGI GWA permutations tested (Table S3). Hence, only the first five out of six properties listed in Table 1 were considered here. At each step of the regression analysis, an additional variable (gene score confounder) is added for consideration under the regression model. Variables with *p*<0.05 were considered significant and included in the regression model, and variables with *p*>0.1 were removed from the model. Variables are listed in the table in the order they were added to the model. Similar ß coefficients and *p*-values were obtained within each study using either −110kb/+40kb gene boundaries or ±50kb boundaries. The main differences between the DGI GWA study and the DIAGRAM+ meta-analysis were in the ß coefficients of SNP density and of linkage disequilibrium unit density. The ß coefficient for SNP density is smaller in the DIAGRAM+ meta-analysis compared to the DGI study, possibly because the overall SNP density is much larger in the meta-analysis (∼6-fold higher), which may decrease the difference in SNP density between small and large genes. The linkage disequilibrium unit gene property was not considered significant for the DGI study. This may also be due to differences in SNP density, since a lower SNP density may decrease the fraction of SNPs in a given chromosomal region that are in strong linkage disequilibrium. ^†^All gene properties aside for gene size were divided by the size of the gene and its extended physical boundaries. *p-value is the probability for testing the null hypothesis that ß = 0 (i.e. probability that a variable should not be added to the regression model).(0.13 MB PDF)

Table S5GSEA results for lipid and lipoprotein-related pathways using LDL cholesterol GWA meta-analysis of 19,840 individuals. A total of 51 (partially overlapping) gene sets related to lipid, lipoprotein and fatty acid metabolism taken from the PANTHER and Gene Ontology databases were tested with MAGENTA for enrichment of genetic associations to LDL cholesterol blood levels, using a GWA meta-analysis of 19,840 individuals (Kathiresan S. *et al.*, 2009, Nature Genetics 41: 56–65). GSEA *p*-values that passed the Bonferroni significance threshold were marked with an asterisk (each database was corrected for multiple hypothesis testing separately due to considerable overlap between the gene sets from the different databases). The Bonferroni cutoffs for the different databases are: PANTHER (15 pathways): *p*<0.0033, Gene Ontology, biological process terms (29 gene sets): *p*<0.0017, and Gene Ontology, molecular function terms (7 gene sets): *p*<0.0071. In the third column, GSEA *p*-values in parentheses are following exclusion of 19 genes that lie near 11 validated SNPs associated with LDL cholesterol (taken from Table 2 in Kathiresan S. *et al.*, 2009). Interestingly, the association signals of some of the gene sets, including lipid and lipoprotein metabolism and lipid transport processes are still detectable when genes near validated SNPs are removed from the GSEA analysis. The 95^th^ percentile of the adjusted LDL gene association *p*-values (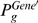) for all genes in the genome was used as the gene set enrichment cutoff.(0.05 MB PDF)

Table S6GSEA results for lipid and lipoprotein-related pathways using HDL cholesterol GWA meta-analysis of 19,840 individuals. A total of 51 (partially overlapping) gene sets related to lipid, lipoprotein and fatty acid metabolism taken from the PANTHER and Gene Ontology databases were tested with MAGENTA for enrichment of genetic associations to HDL cholesterol blood levels, using a GWA meta-analysis of 19,840 individuals (Kathiresan S. *et al.*, 2009, Nature Genetics 41: 56–65). GSEA *p*-values that passed the Bonferroni significance threshold were marked with an asterisk (each database was corrected for multiple hypothesis testing separately due to considerable overlap between the gene sets from the different databases). The Bonferroni cutoffs for the different databases are: PANTHER (15 pathways): *p*<0.0033, Gene Ontology, biological process terms (29 gene sets): *p*<0.0017, and Gene Ontology, molecular function terms (7 gene sets): *p*<0.0071. In the third column, GSEA *p*-values in parentheses are following exclusion of 20 genes that lie near 14 validated SNPs associated with HDL cholesterol (taken from Table 2 in Kathiresan S. *et al.*, 2009). Interestingly, the association signals of some of the gene sets, including lipid metabolism, binding and transport processes and triacylglycerol metabolism are still detectable when genes near validated HDL cholesterol SNPs are removed from the GSEA analysis. The 95^th^ percentile of the adjusted HDL gene association p-values (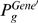) for all genes in the genome was used as the gene set enrichment cutoff.(0.05 MB PDF)

Table S7GSEA results for lipid and lipoprotein-related pathways using triglyceride GWA meta-analysis of 19,840 individuals. A total of 51 (partially overlapping) gene sets related to lipid, lipoprotein and fatty acid metabolism taken from the PANTHER and Gene Ontology databases were tested with MAGENTA for enrichment of genetic associations to triglyceride blood levels, using a GWA meta-analysis of 19,840 individuals (Kathiresan S. *et al.*, 2009, Nature Genetics 41: 56–65). GSEA *p*-values that passed the Bonferroni significance threshold were marked with an asterisk (each database was corrected for multiple hypothesis testing separately due to considerable overlap between the gene sets from the different databases). The Bonferroni cutoffs for the different databases are: PANTHER (15 pathways): *p*<0.0033, Gene Ontology, biological process terms (29 gene sets): *p*<0.0017, and Gene Ontology, molecular function terms (7 gene sets): *p*<0.0071. In the third column, GSEA *p*-values in parentheses are following exclusion from the analysis of 19 genes that lie near 11 validated SNPs associated with triglyceride levels (taken from Table 2 in Kathiresan S. *et al.*, 2009). Interestingly, the association signals of some of the gene sets, in particular phospholipid binding and metabolic processes are still detectable when genes near validated SNPs are removed from the GSEA analysis. The 95^th^ percentile of the adjusted triglyceride gene association p-values (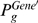) for all genes in the genome was used as the gene set enrichment cutoff.(0.05 MB PDF)

Table S8GSEA of LDL cholesterol GWA meta-analysis is robust to the gene score correction method used. GSEA results for lipid and lipoprotein-related gene sets using a GWA meta-analysis of LDL cholesterol blood levels (Kathiresan S. *et al.*, 2009, Nature Genetics 41: 56–65) are presented following two different gene score correction methods: a modified version of Sidak's correction, proposed by Saconne *et al.* (Saccone SF *et al.*, Human Molecular Genetics 16(1): 36–49, 2007) (column 3) and a step-wise multivariate regression analysis method (column 4). GSEA *p*-values that passed the Bonferroni significance threshold are marked with an asterisk (each database was corrected for multiple hypothesis testing separately, due to considerable overlap between the gene sets from the different databases). The GSEA results are quite robust to the correction method used. In the third and fourth columns, GSEA *p*-values in parentheses are following exclusion of 19 genes that lie near 11 validated SNPs associated with LDL cholesterol (taken from Table 2 in Kathiresan S. *et al.*, 2009). The number of genes analyzed by MAGENTA in column 2 was taken from the analysis that applied the modified Sidak's correction of gene *p*-values. This number was in most cases identical to that following regression-based correction (Table S5). The 95^th^ percentile of the adjusted LDL cholesterol gene association *p*-values (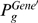) for all genes in the genome was used as the gene set enrichment cutoff.(0.06 MB PDF)

Table S9GSEA of HDL cholesterol GWA meta-analysis is robust to the gene score correction method used. GSEA results for lipid and lipoprotein-related gene sets using a GWA meta-analysis of HDL cholesterol blood levels (Kathiresan S. *et al.*, 2009, Nature Genetics 41: 56–65) are presented following two different gene score correction methods: a modified version of Sidak's correction, proposed by Saconne *et al.* (Saccone SF *et al.*, Human Molecular Genetics 16(1): 36–49, 2007) (column 3) and a step-wise multivariate regression analysis method (column 4). GSEA *p*-values that passed the Bonferroni significance threshold are marked with an asterisk (each database was corrected for multiple hypothesis testing separately, due to considerable overlap between the gene sets from the different databases). The GSEA results are quite robust to the correction method used. GSEA *p*-values in parentheses are following exclusion from the analysis of 20 genes that lie near 14 validated SNPs associated with HDL cholesterol (taken from Table 2 in Kathiresan S. *et al.*, 2009). The number of genes analyzed by MAGENTA in column 2 was taken from the analysis that used the modified Sidak's correction of gene *p*-values. This number was in most cases identical to that following regression-based correction (Table S5). The 95^th^ percentile of the adjusted HDL cholesterol gene association *p*-values (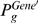) for all genes in the genome was used as the gene set enrichment cutoff.(0.05 MB PDF)

Table S10GSEA of triglyceride GWA meta-analysis is robust to the gene score correction method used. GSEA results for lipid and lipoprotein-related gene sets using a GWA meta-analysis of triglyceride blood levels (Kathiresan S. *et al.*, 2009, Nature Genetics 41: 56–65) are presented following two different gene score correction methods: a modified version of Sidak's correction, proposed by Saconne *et al.* (Saccone SF *et al.*, Human Molecular Genetics 16(1): 36–49, 2007) (column 3) and a step-wise multivariate regression analysis method (column 4). GSEA *p*-values that passed the Bonferroni significance threshold are marked with an asterisk (each database was corrected for multiple hypothesis testing separately, due to considerable overlap between the gene sets from the different databases). The GSEA results are quite robust to the correction method used. GSEA *p*-values in parentheses are following exclusion from the analysis of 19 genes that lie near 11 validated SNPs associated with triglyceride blood levels (list of known genes taken from Table 2 in Kathiresan S. *et al.*, 2009). The number of genes analyzed by MAGENTA in column 2 was taken from the analysis that used the modified Sidak's correction method. This number was in most cases identical to that following regression-based correction (Table S5). The 95^th^ percentile of the adjusted triglyceride gene association *p*-values (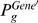) for all genes in the genome was used as the gene set enrichment cutoff.(0.05 MB PDF)

Table S11List of nuclear regulators of mitochondrial genes and their T2D association scores. T2D gene *p*-values adjusted for confounding effects with step-wise multivariate linear regression analysis (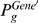) were computed for 16 known nuclear regulators of nuclear-encoded mitochondrial genes, using the DIAGRAM+ T2D GWA study meta-analysis (Voight BF *et al.*, Nature Genetics, in press, 2010).(0.02 MB XLS)

Table S12List of T2D gene association scores for all known autosomal mitochondrial genes taken from MitoCarta. T2D gene *p*-values adjusted for confounders with multivariate linear regression analysis (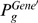) are listed for 966 nuclear-encoded (autosomal) mitochondrial genes, taken from the MitoCarta compendium (Pagliarini DJ, *et al.*, 2008, Cell 134: 112–123). The DIAGRAM+ T2D GWA study meta-analysis (Voight BF, *et al.*, Nature Genetics, in press, 2010) was used here. Genes in bold lie near validated T2D SNPs, as of the DIAGRAM+ meta-analysis. The last column marks genes that belong to the oxidative phosphorylation (OXPHOS) pathway with the number 1. ‘NaN’ refers to genes that had no SNPs in their extended gene boundaries (110kb upstream to the gene's most extreme transcript start site, and 40kb downstream to the gene's transcript most extreme end site).(0.23 MB XLS)

Table S13GSEA results of mitochondria-related gene sets are robust to GSEA statistical test used. We tested the robustness of the mitochondria-related gene set enrichment results with respect to T2D and seven diabetes-relevant glycemic traits by using an alternative GSEA statistical test to the enrichment cutoff approach. A one-tailed Mann-Whitney rank-sum test was applied (described in Materials and Methods) to GWA study meta-analyses of T2D and seven glucose and insulin-related traits. 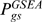 is the nominal gene set enrichment *p*-value for gene set *gs* computed for each phenotype separately. The enrichment cutoff used was the 95^th^ percentile of all gene *p*-values computed from the corresponding GWA meta-analysis. The GSEA results obtained with the rank-sum approach are very similar to those obtained using the enrichment cutoff approach (see Table 3 and Table 4). HOMA-IR is an index for insulin resistance, HOMA-B is an index for β-cell function, and HbA_1C_ represents glycated hemoglobin concentrations. OXPHOS stands for the oxidative phosphorylation process. The nuclear regulators are regulators of nuclear-encoded mitochondrial genes. ^‡^These gene sets are not significant after Bonferroni correction (most stringent cutoff *p*<0.002, given 3 gene sets and 8 traits tested; a less stringent cutoff *p*<0.0083, correcting for 3 gene sets and 2 traits due to considerable correlation between the glucose and insulin-related traits).(0.15 MB PDF)

Table S14GSEA results of mitochondria-related gene sets are robust to the gene score correction method used. We tested the effect of using a different gene score correction method other than the regression-based method on mitochondria-related gene set enrichment results with respect to type 2 diabetes and seven related glycemic traits. We applied a modification of the Sidak's correction (described in Materials and Methods; Saccone SF *et al.*, Human Molecular Genetics 16(1): 36–49, 2007) to correct for confounding effects on the most significant SNP *p*-value, 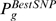 for each gene *g*. 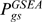 is the nominal gene set enrichment (GSEA) *p*-value for gene set *gs* computed for each phenotype separately. The enrichment cutoff used was the 95^th^ percentile of all gene scores computed from the corresponding GWA study meta-analysis. HOMA-IR is an index for insulin resistance, HOMA-B is an index for β-cell function, and HbA_1C_ represents glycated hemoglobin concentrations. OXPHOS stands for the oxidative phosphorylation process. The nuclear regulators are regulators of nuclear-encoded mitochondrial genes. ^‡^ This gene set is not significant after Bonferroni correction (most stringent cutoff *p*<0.002, given 3 gene sets and 8 traits tested; a less stringent cutoff *p*<0.0083, correcting for 3 gene sets and 2 traits due to considerable correlation between the glucose and insulin-related traits and type 2 diabetes). The GSEA results are comparable to those using step-wise multivariate linear regression analysis to correct for confounders on gene association *p*-values (Table 3 and Table 4).(0.16 MB PDF)

Text S1 Lists of consortia participants and affiliations.
(0.10 MB DOC)
