# Supplementary material for: Common Inherited Variation in Mitochondrial Genes Is Not Enriched for Associations with Type 2 Diabetes or Related Glycemic Traits
Source: PLoS Genet. 2010 Aug 12;6(8):e1001058. doi: 10.1371/journal.pgen.1001058 (PMC2920848; doi:10.1371/journal.pgen.1001058)
Supplement: Figure S1 — Cumulative distribution of mitochondrial and non-mitochondrial gene scores before and after adjustment for confounders. The cumulative p-value distributions are plotted for the most significant SNP T2D association p-value within each gene's extended boundaries (A) before and (B) after adjustment for gene score confounders. The distributions are plotted for 966 autosomal mitochondrial genes (red line), the oxidative phosphorylation (OXPHOS) subset (green line), and the rest of the genes in the genome that have at least one SNP in their region (non-mitochondrial genes; blue line) (see Materials and Methods for details). The correction presented in panel B is following a step-wise multivariate linear regression analysis of the most significant SNP p-value against the first five gene properties listed in Table 1. The x-axis is on a log10 scale in both panels. (0.25 MB PDF) [file pgen.1001058.s001.pdf]

Figure S1

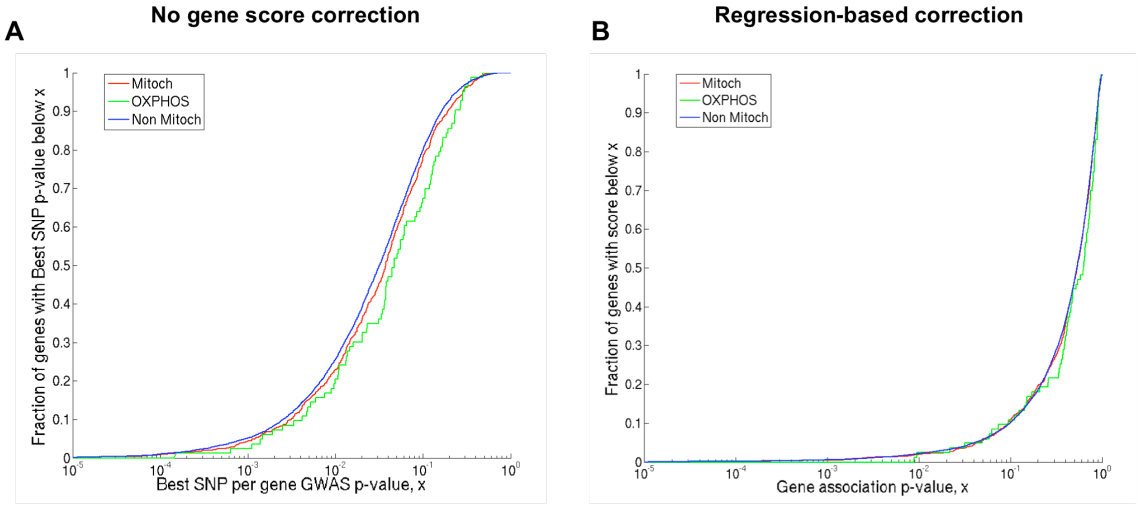

Figure S1. Cumulative distribution of mitochondrial and non-mitochondrial gene scores before and after adjustment for confounders. The cumulative *p*-value distributions are plotted for the most significant SNP T2D association *p*-value within each gene's extended boundaries (A) before and (B) after adjustment for gene score confounders. The distributions are plotted for 966 autosomal mitochondrial genes (red line), the oxidative phosphorylation (OXPHOS) subset (green line), and the rest of the genes in the genome that have at least one SNP in their region (non-mitochondrial genes; blue line) (see Materials and Methods for details). The correction presented in panel B is following a step-wise multivariate linear regression analysis of the most significant SNP *p*-value against the first five gene properties listed in Table 1. The x-axis is on a $\log_{10}$ scale in both panels.
