# Supplementary material for: Common Inherited Variation in Mitochondrial Genes Is Not Enriched for Associations with Type 2 Diabetes or Related Glycemic Traits
Source: PLoS Genet. 2010 Aug 12;6(8):e1001058. doi: 10.1371/journal.pgen.1001058 (PMC2920848; doi:10.1371/journal.pgen.1001058)
Supplement: Figure S2 — Distribution of T2D gene p-values for small, large and all genes before and after correction for confounders. (A) The distribution of the mean (best SNP association p-value per gene g) calculated across 1,000 phenotype permutations of the Diabetes Genetics Initiative (DGI) GWA study is shown for all genes in genome (blue line), only large genes (≥100 kilobase (kb); red line), and only small genes (≤10 kb; green line). Large genes tended to receive on average a more significant gene score (lower p-values) than all genes in the permuted datasets, and small genes tended to receive on average a less significant gene score (higher p-values) than all genes. (B–D) The distribution of gene association p-values is shown for the actual DGI study for all gene sizes (blue line), large genes (red line) and small genes (green line) (B) before correcting for confounders (), and after correcting for confounders on , such as gene size, using either (C) phenotype permutation analysis () or (D) step-wise multivariate linear regression analysis (). The regression-based correction transforms the gene p-values to a distribution that is close to uniform and removes the confounding effect of gene size, similar to the permutation-based correction, which corrects for all confounding effects without a priori knowledge of them. The regression correction seems to slightly over-correct the gene p-values of large genes (red line in D) in the high p-value end of the distribution (p>0.8). A bin of 0.01 was used for all four plots. (0.66 MB PDF) [file pgen.1001058.s002.pdf]

Figure S2

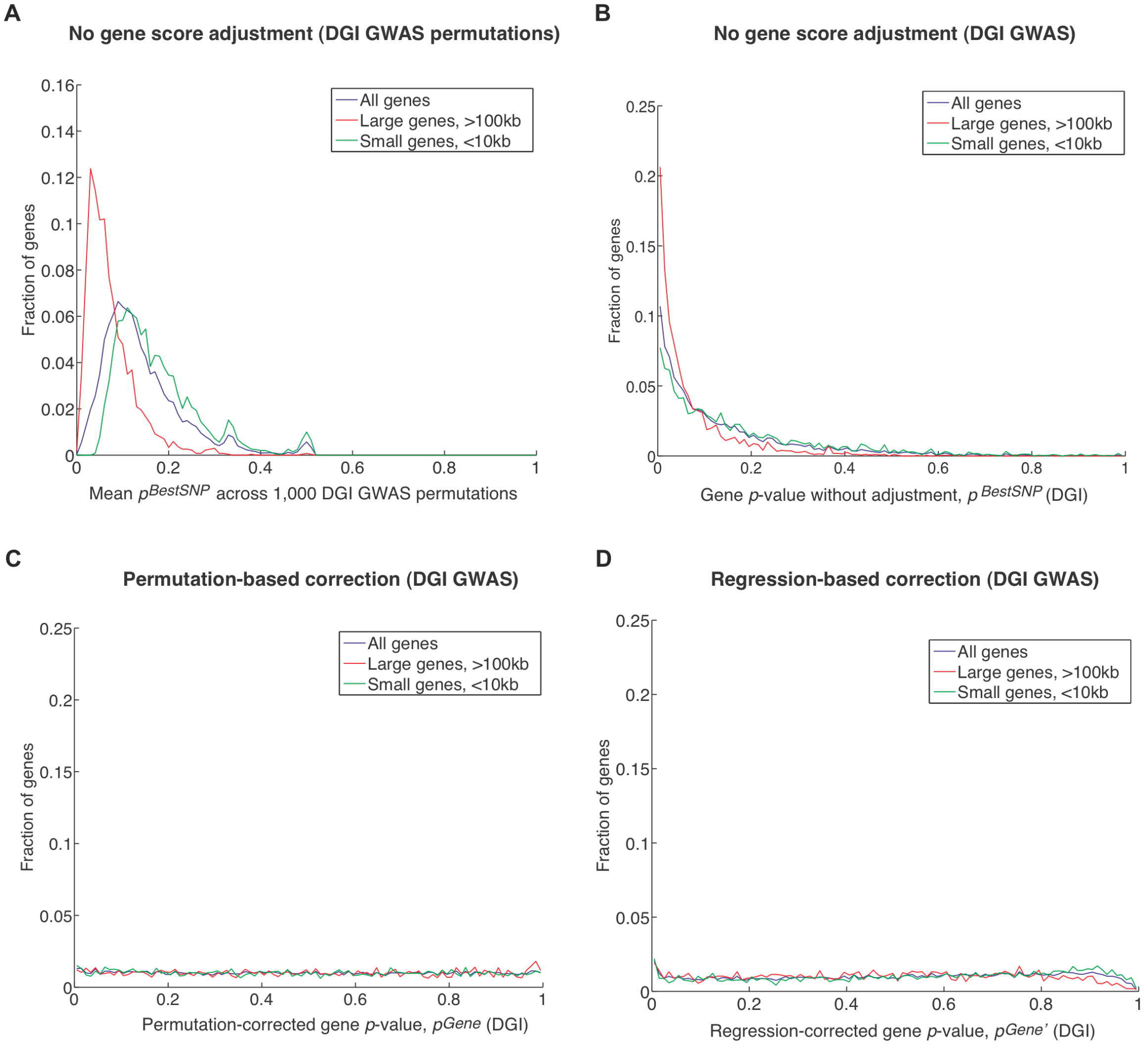

Figure S2. Distribution of T2D gene *p*-values for small, large and all genes before and after correction for confounders. (A) The distribution of the mean $P_g^{BestSNP}$ (best SNP association *p*-value per gene *g*) calculated across 1,000 phenotype permutations of the Diabetes Genetics Initiative (DGI) GWA study is shown for all genes in genome (blue line), only large genes (≥100 kilobase (kb); red line), and only small genes (≤10 kb; green line). Large genes tended to receive on average a more significant gene score (lower *p*-values) than all genes in the permuted datasets, and small genes tended to receive on average a less significant gene score (higher *p*-values) than all genes. (B-D)

The distribution of gene association *p*-values is shown for the actual DGI study for all gene sizes (blue line), large genes (red line) and small genes (green line) (B) before correcting for confounders ($P_g^{BestSNP}$), and after correcting for confounders on $P_g^{BestSNP}$, such as gene size, using either (C) phenotype permutation analysis (P_g^{Gene}) or (D) step-wise multivariate linear regression analysis ($P_g^{Gene'}$). The regression-based correction transforms the gene *p*-values to a distribution that is close to uniform and removes the confounding effect of gene size, similar to the permutation-based correction, which corrects for all confounding effects without *a priori* knowledge of them. The regression correction seems to slightly over-correct the gene *p*-values of large genes (red line in D) in the high *p*-value end of the distribution (p>0.8). A bin of 0.01 was used for all four plots.
