# Supplementary material for: Common Inherited Variation in Mitochondrial Genes Is Not Enriched for Associations with Type 2 Diabetes or Related Glycemic Traits
Source: PLoS Genet. 2010 Aug 12;6(8):e1001058. doi: 10.1371/journal.pgen.1001058 (PMC2920848; doi:10.1371/journal.pgen.1001058)
Supplement: Figure S3 — A comparison of the performance of several gene association score correction methods. T2D gene association p-values were plotted (A) before gene score adjustment () and after correction for potential SNP-to-gene score confounders (), as a function of gene p-values corrected with phenotype permutation analysis (). The correction methods tested: (B) step-wise multivariate linear regression analysis, (C) Sidak's correction (eq. 4 in Materials and Methods) and (D) a modified version of Sidak's correction (eq. 5 in Materials and Methods; Saccone SF et al., Human Molecular Genetics 16(1): 36–49, 2007). The Diabetes Genetics Initiative (DGI) study was used for the analysis, as we had access to genotype data in this study. The unadjusted gene p-value, is the association p-value of the best regional SNP for gene g (y-axis in A). Phenotype permutation analysis was used as the gold standard to test goodness of gene score correction as it corrects for all confounders without requiring a priori knowledge of the confounders (). The Pearson's correlation coefficient (calculated between p-value vectors before log transformation) increased significantly following each of the three correction methods (from r = 0.69 to r = 0.94–0.97), but the Sidak's correction (C) did not perform as well, as it tends to overcorrect (most of the dots fall below the diagonal, the red line). The spread around the diagonal also decreased for all three correction methods. While the modified Sidak's correction (D) performs a bit better than the regression-based correction (B) in the DGI study, Figure S4 shows that its performance varies between GWA studies of different SNP densities. The correction for linkage between SNPs in the modified Sidak's correction equation may need to be adjusted for different GWA studies or meta-analyses with different SNP densities (see Figure S4 for details). The minimum is 10−4 as the p-values were calculated based on 1,000 permutations for genes with and 10,000 permutations [file pgen.1001058.s003.pdf]

Figure S3

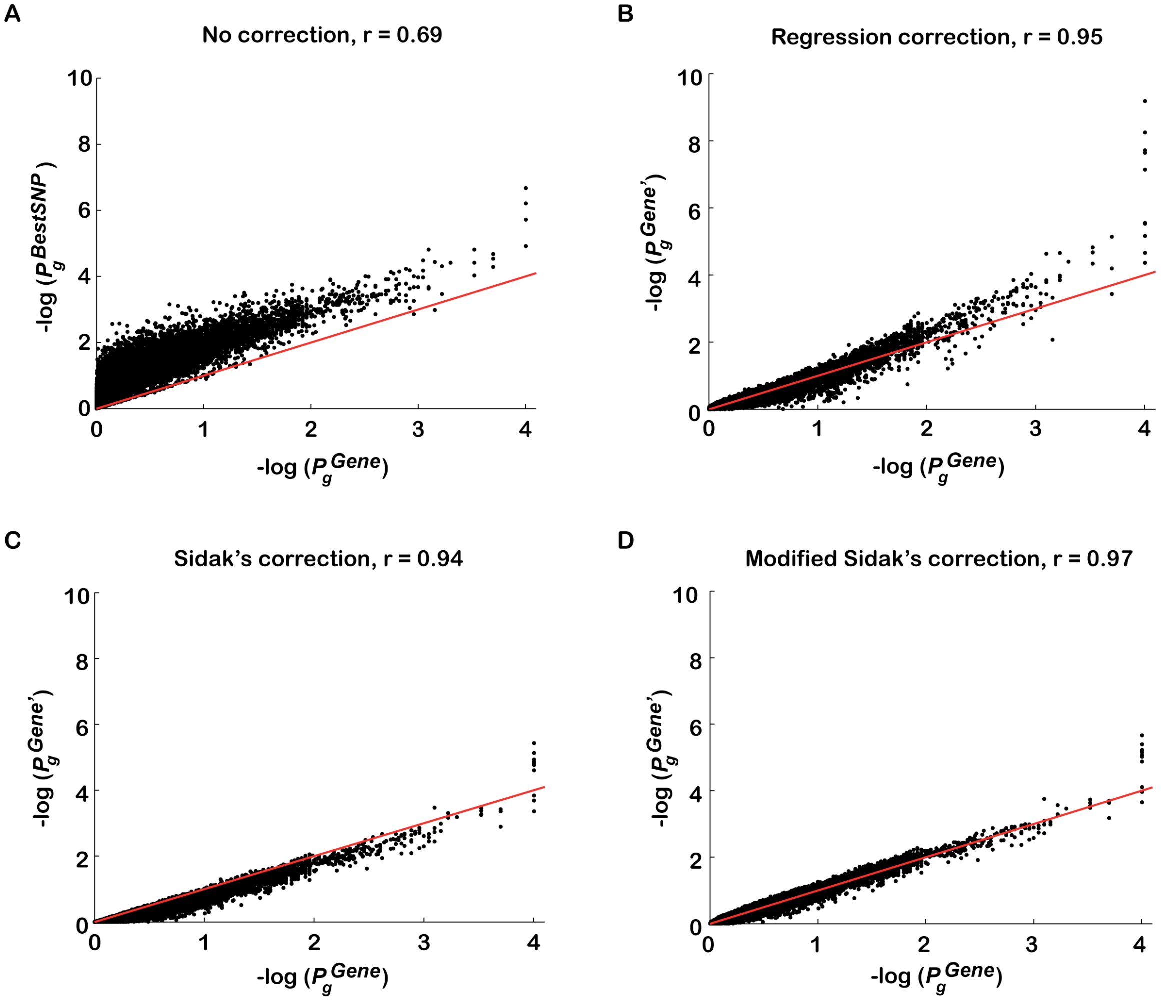

Figure S3. A comparison of the performance of several gene association score correction methods. T2D gene association *p*-values were plotted (A) before gene score adjustment ($P_g^{BestSNP}$) and after correction for potential SNP-to-gene score confounders ($P_g^{Gene'}$) for each gene *g* in the genome, as a function of gene *p*-values corrected with phenotype permutation analysis (P_g^{Gene}). The correction methods tested: (B) step-wise multivariate linear regression analysis, (C) Sidak's correction (eq. 4 in Materials and Methods) and (D) a modified version of Sidak's correction (eq. 5 in Materials and Methods; Saccone SF *et al.*, Human Molecular Genetics 16(1): 36-49, 2007). The Diabetes Genetics Initiative (DGI) study was used for the analysis, as we had access to

genotype data in this study. The unadjusted gene *p*-value, $P_g^{BestSNP}$ is the association *p*-value of the best regional SNP for gene *g* (y-axis in **A**). Phenotype permutation analysis was used as the gold standard to test goodness of gene score correction as it corrects for all confounders without requiring *a priori* knowledge of the confounders (P_g^{Gene}). The Pearson's correlation coefficient (calculated between *p*-value vectors before log transformation) increased significantly following each of the three correction methods (from r=0.69 to r=0.94-0.97), but the Sidak's correction (C) did not perform as well, as it tends to overcorrect (most of the dots fall below the diagonal, the red line). The spread around the diagonal also decreased for all three correction methods. While the modified Sidak's correction (D) performs a bit better than the regression-based correction (B) in the DGI study, Figure S4 shows that its performance varies between GWA studies of different SNP densities. The correction for linkage between SNPs in the modified Sidak's correction equation may need to be adjusted for different GWA studies or meta-analyses with different SNP densities (see Figure S4 for details). The minimum P_g^{Gene} is 10^{-4} as the *p*-values were calculated based on 1,000 permutations for genes with $P_g^{Gene} > 0.01$ and 10,000 permutations for genes with $P_g^{Gene} \leq 0.01$. Gene *p*-values are plotted on a $-\log_{10}(p\text{-value})$ scale.
