# Supplementary material for: Common Inherited Variation in Mitochondrial Genes Is Not Enriched for Associations with Type 2 Diabetes or Related Glycemic Traits
Source: PLoS Genet. 2010 Aug 12;6(8):e1001058. doi: 10.1371/journal.pgen.1001058 (PMC2920848; doi:10.1371/journal.pgen.1001058)
Supplement: Figure S4 — Distribution of gene association p-values for different T2D GWA studies and gene score correction methods. Presented here are the distributions of the best SNP per gene p-values for all genes after adjustment for confounders (), using two different correction methods: (A–B) a step-wise multivariate linear regression analysis that regresses out physical and linkage-related confounders from the most significant SNP association z-score, and (C–D) a modification of the Sidak's correction equation that uses an exponent of about half the number of SNPs per gene to adjust for linkage disequilibrium between SNPs in a given chromosomal region (eq. 5 in Materials and Methods). A bin of 0.01 was used in all four panels. The distribution of following regression analysis is similar for the DGI study (A) that contains ∼3.8e5 genotyped SNPs (on average 1 SNP/8kb) and the DIAGRAM+ T2D meta-analysis (B) that contains ∼2.3e6 genotyped or imputed SNPs (on average 1 SNP/1.3kb). The regression-corrected distributions in both studies are close to uniform, aside for an excess in the low p-value tail and a slight deviation from uniformity in the high p-value tail. Panels A and B show that the regression correction, which explicitly takes into account linkage disequilibrium properties between SNPs in a gene-specific manner, is adjustable to studies with different SNP densities and linkage properties. The distribution of following the modified Sidak's method is also close to uniform in the DGI study (C). However, in the DIAGRAM+ meta-analysis, which contains about 6-fold more SNPs than the DGI study, the modified Sidak's correction distribution is largely skewed towards high values of (D) (∼11.4% of genes with where only 0.1% is expected, and ∼19.4% of genes with where only 1% is expected). This difference in performance of the modified Sidak correction between the DGI and DIAGRAM+ studies may be due to differences in SNP density, which may affect the effective fraction of SNPs that are in t [file pgen.1001058.s004.pdf]

Figure S4

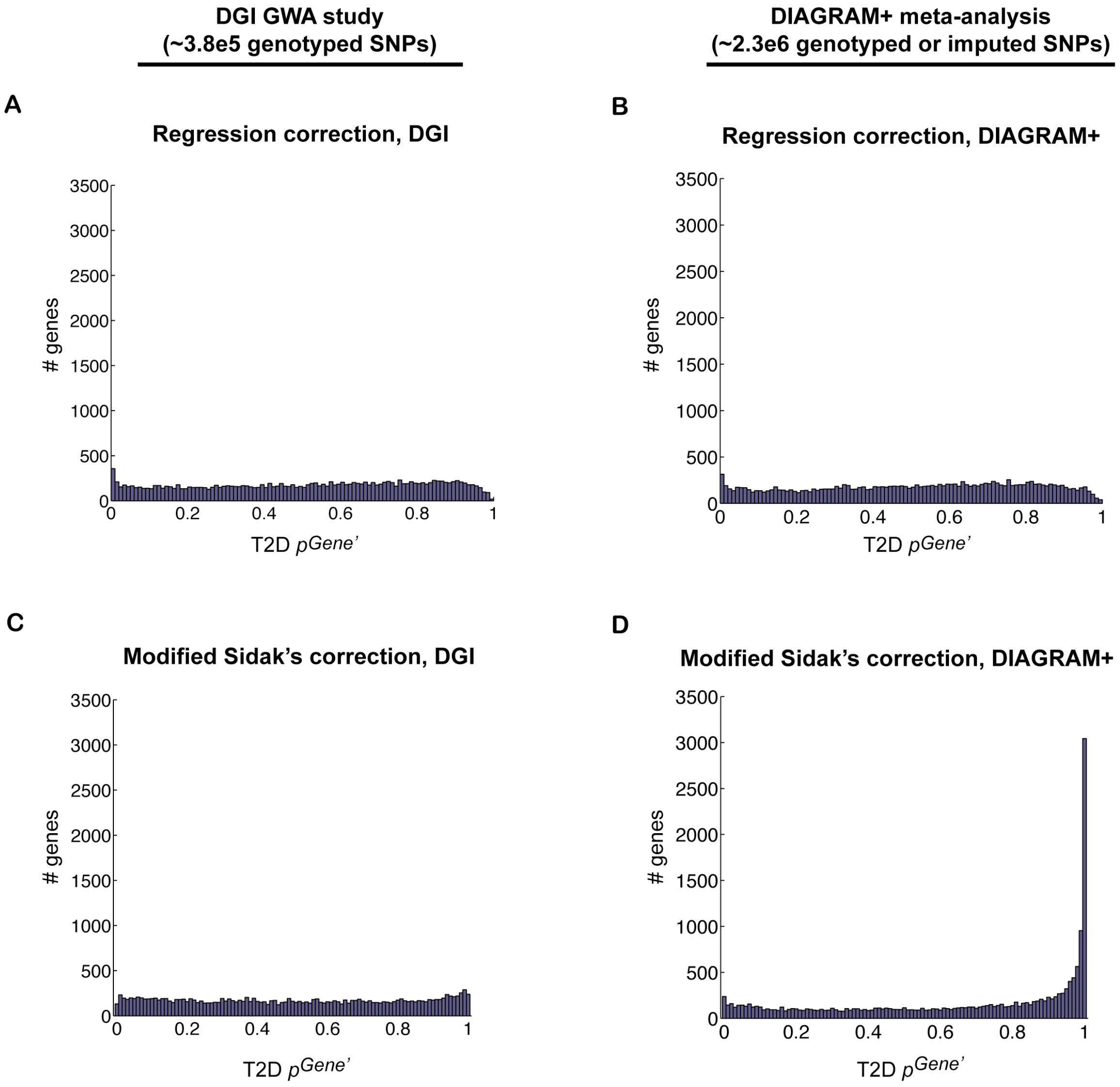

Figure S4. Distribution of gene association *p*-values for different T2D GWA studies and gene score correction methods. Presented here are the distributions of the best SNP per gene *p*-values for all genes after adjustment for confounders ($P_g^{Gene'}$), using two different correction methods: (A-B) a step-wise multivariate linear regression analysis that regresses out physical and linkage-related confounders from the most significant SNP association z-score, and (C-D) a modification of the Sidak's correction equation that uses an exponent of about half the number of SNPs per gene to adjust for linkage disequilibrium between SNPs in a given chromosomal region (eq. 5 in Materials and

Methods). A bin of 0.01 was used in all four panels. The distribution of $P_g^{Gene'}$ following regression analysis is similar for the DGI study (A) that contains ~3.8e5 genotyped SNPs (on average 1 SNP/8kb) and the DIAGRAM+ T2D meta-analysis (B) that contains ~2.3e6 genotyped or imputed SNPs (on average 1 SNP/1.3kb). The regression-corrected $P_g^{Gene'}$ distributions in both studies are close to uniform, aside for an excess in the low *p*-value tail and a slight deviation from uniformity in the high p-value tail. Panels A and B show that the regression correction, which explicitly takes into account linkage disequilibrium properties between SNPs in a gene-specific manner, is adjustable to studies with different SNP densities and linkage properties. The distribution of $P_g^{Gene'}$ following the modified Sidak's method is also close to uniform in the DGI study (C). However, in the DIAGRAM+ meta-analysis, which contains about 6-fold more SNPs than the DGI study, the modified Sidak's correction distribution is largely skewed towards high values of $P_g^{Gene'}$ (D) (~11.4% of genes with $P_g^{Gene'} > 0.999$ where only 0.1% is expected, and ~19.4% of genes with $P_g^{Gene'} > 0.99$ where only 1% is expected). This difference in performance of the modified Sidak correction between the DGI and DIAGRAM+ studies may be due to differences in SNP density, which may affect the effective fraction of SNPs that are in tight linkage disequilibrium in different regions along the genome. Hence, the exponent in Sidak's equation (eq. 5 in Materials and Methods) might need to be adjusted for different studies.
