# Supplementary material for: Common Inherited Variation in Mitochondrial Genes Is Not Enriched for Associations with Type 2 Diabetes or Related Glycemic Traits
Source: PLoS Genet. 2010 Aug 12;6(8):e1001058. doi: 10.1371/journal.pgen.1001058 (PMC2920848; doi:10.1371/journal.pgen.1001058)
Supplement: Figure S5 — Using simulations to find an optimal gene set enrichment cutoff. The power of detecting gene set enrichment of multiple modest (A) or weak (B) effects was estimated with simulations as a function of fractions of causal genes in a gene set of 100 genes, for five different enrichment cutoffs: 99th percentile (black line), 95th percentile (dark blue line), 90th percentile (green line), 75th percentile (red line), or 50th percentile (cyan line) of all corrected gene p-values (with regression analysis). The modest effect size in (A) represents 1% power of detecting an association at genome-wide significance (p-value<5e-8) using single SNP analysis, and the weak effect size in (B) represents 1% power of detecting an association at p-value<1e-4 using single SNP analysis. A total of 100 causal genes in the genome was assumed here. These plots show that power of MAGENTA to detect enrichment of multiple modest effects is fairly robust to the enrichment cutoff used. Overall, the 95th percentile cutoff performed the best. While the 99th and 95th percentile cutoffs performed similarly in detecting enrichment of multiple modest effects (A), the 95th percentile cutoff performed significantly better in detecting enrichment of many weak effects (B). Note the log10 scale of the x-axis in both panels. (0.41 MB PDF) [file pgen.1001058.s005.pdf]

**Figure S5**

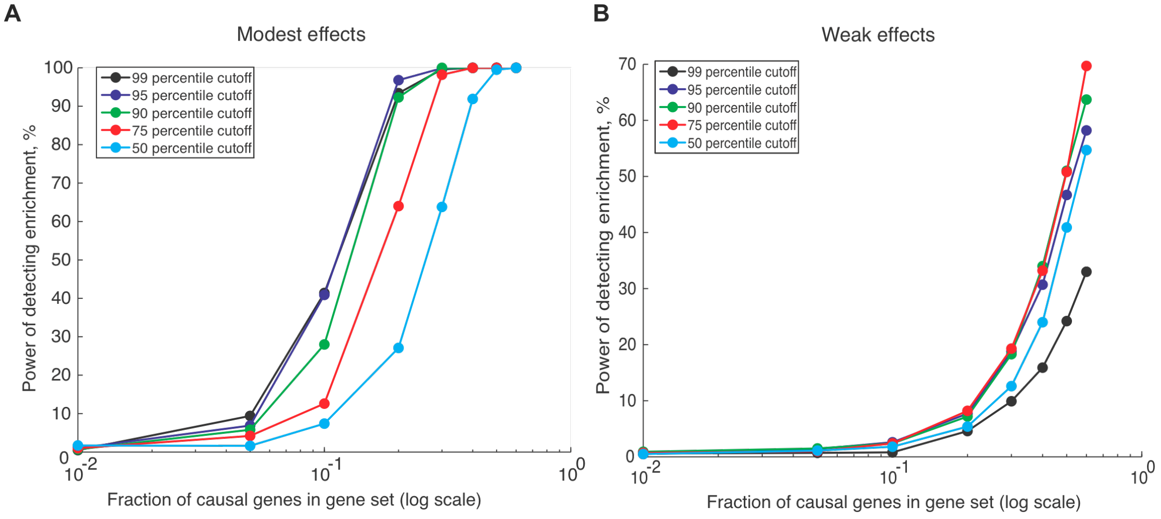


**Figure S5. Using simulations to find an optimal gene set enrichment cutoff.** The power of detecting gene set enrichment of multiple modest (A) or weak (B) effects was estimated with simulations as a function of fractions of causal genes in a gene set of 100 genes, for five different enrichment cutoffs: 99$^{th}$ percentile (black line), 95$^{th}$ percentile (dark blue line), 90$^{th}$ percentile (green line), 75$^{th}$ percentile (red line), or 50$^{th}$ percentile (cyan line) of all corrected gene *p*-values (with regression analysis). The modest effect size in **A** represents 1% power of detecting an association at genome-wide significance (*p*-value<5e-8) using single SNP analysis, and the weak effect size in **B** represents 1% power of detecting an association at *p*-value<1e-4 using single SNP analysis. A total of 100 causal genes in the genome was assumed here. These plots show that power of MAGENTA to detect enrichment of multiple modest effects is fairly robust to the enrichment cutoff used. Overall, the 95$^{th}$ percentile cutoff performed the best. While the 99$^{th}$ and 95$^{th}$ percentile cutoffs performed similarly in detecting enrichment of multiple modest effects (A), the 95$^{th}$ percentile cutoff performed significantly better in detecting enrichment of many weak effects (B). Note the $\log_{10}$ scale of the x-axis in both panels.
