# Supplementary material for: Common Inherited Variation in Mitochondrial Genes Is Not Enriched for Associations with Type 2 Diabetes or Related Glycemic Traits
Source: PLoS Genet. 2010 Aug 12;6(8):e1001058. doi: 10.1371/journal.pgen.1001058 (PMC2920848; doi:10.1371/journal.pgen.1001058)
Supplement: Figure S6 — Power of MAGENTA as a function of effect size and total number of causal genes in the genome. (A) Power of detecting gene set enrichment of multiple modest associations increases with effect size. Using computer simulations we assessed the power of MAGENTA to detect enrichment of multiple SNPs of modest effect spiked into various fractions of genes (causal genes) in a gene set size of 100 genes (one SNP per gene). Two different effect sizes were tested: (i) the modest effect (solid line) represents 1% power of detecting a SNP association at genome-wide significance (p-value<5e-8) using single SNP analysis, and the weak effect (dashed line) represents 1% power of detecting an association at p-value<1e-4 using single SNP analysis (details in Materials and Methods). A similar trend was obtained for a gene set size of 25 and 1,000 genes (data not shown). The false positive rate for the parameters used here was between 0.4–1.7%. (B) Power of detecting gene set enrichment of modest associations decreases as the total number of causal genes in the genome increases. Power was estimated assuming a total of 100 (solid line) or 500 (dashed line) causal genes in the genome. For both panels a gene set was considered significant at a GSEA p-value cutoff of <0.01. Note the logarithmic scale of the x-axis for both plots. (0.28 MB PDF) [file pgen.1001058.s006.pdf]

Figure S6

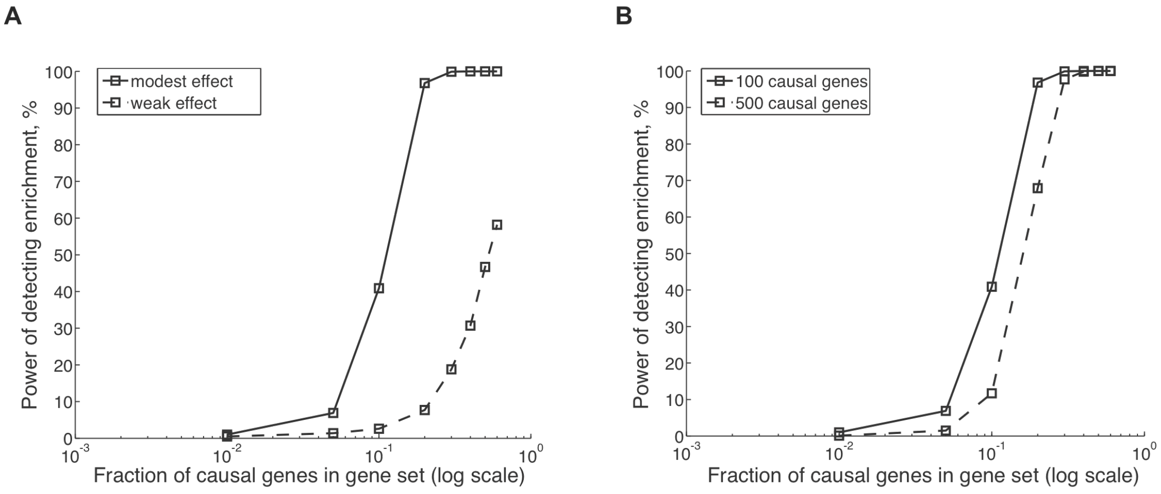

Figure S6. Power of MAGENTA as a function of effect size and total number of causal genes in the genome. (A) Power of detecting gene set enrichment of multiple modest associations increases with effect size. Using computer simulations we assessed the power of MAGENTA to detect enrichment of multiple SNPs of modest effect spiked into various fractions of genes (causal genes) in a gene set size of 100 genes (one SNP per gene). Two different effect sizes were tested: (i) the modest effect (solid line) represents 1% power of detecting a SNP association at genome-wide significance (*p*-value<5e-8) using single SNP analysis, and the weak effect (dashed line) represents 1% power of detecting an association at *p*-value<1e-4 using single SNP analysis (details in Materials and Methods). A similar trend was obtained for a gene set size of 25 and 1,000 genes (data not shown). The false positive rate for the parameters used here was between 0.4-1.7%. (B) Power of detecting gene set enrichment of modest associations decreases as the total number of causal genes in the genome increases. Power was estimated assuming a total of 100 (solid line) or 500 (dashed line) causal genes in the genome. For both panels a gene set was considered significant at a GSEA *p*-value cutoff of P_{gs}^{GSEA}<0.01. Note the logarithmic scale of the *x*-axis for both plots.
