# Supplementary material for: Common Inherited Variation in Mitochondrial Genes Is Not Enriched for Associations with Type 2 Diabetes or Related Glycemic Traits
Source: PLoS Genet. 2010 Aug 12;6(8):e1001058. doi: 10.1371/journal.pgen.1001058 (PMC2920848; doi:10.1371/journal.pgen.1001058)
Supplement: Figure S7 — Quantile-quantile plots of T2D gene association p-values for mitochondria-related gene sets. The T2D gene association p-values adjusted for confounding effects using step-wise multivariate linear regression analysis, (see Materials and Methods) were plotted for (A) 16 nuclear regulators of mitochondrial genes, (B) 91 oxidative phosphorylation genes, and (C) all known nuclear-encoded autosomal mitochondrial genes with at least one SNP in their region (955 genes), as a function of their corresponding null distributions of assuming a uniform distribution. Three mitochondrial genes that lie near validated T2D SNPs, as of the most recent DIAGRAM+ T2D meta-analysis are labeled in red (IDE, C8orf38, and ACADS). The red line marks the diagonal, and the dashed lines represent 5% and 95% confidence intervals estimated based on 1,000 randomly sampled gene sets from the genome of identical set size to the given gene set. All gene p-values lie within the non-parametric 95% confidence intervals. Similar results were obtained when the observed gene p-values were plotted against an expected distribution that was adjusted according to a non-parametric null distribution, generated based on 1,000 randomly sampled gene sets from the genome of identical size to that of the tested gene set (data not shown). is plotted on a −log10(p-value) scale. Note the x and y-axes of the three plots are not on the same scale. (0.31 MB PDF) [file pgen.1001058.s007.pdf]

Figure S7

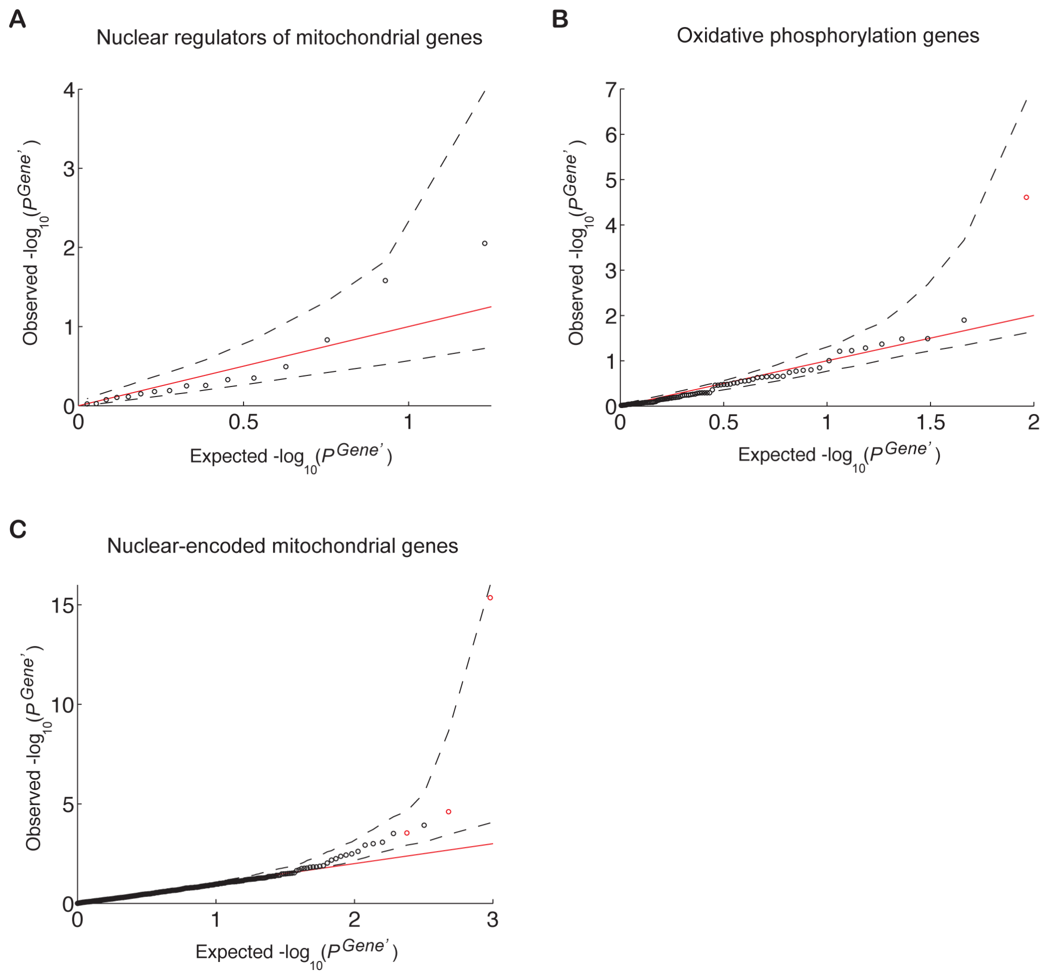

Figure S7. Quantile-quantile plots of T2D gene association *p*-values for mitochondria-related gene sets. The T2D gene association *p*-values adjusted for confounding effects using step-wise multivariate linear regression analysis, $P_g^{Gene'}$ (see Materials and Methods) were plotted for (A) 16 nuclear regulators of mitochondrial genes, (B) 91 oxidative phosphorylation genes, and (C) all known nuclear-encoded autosomal mitochondrial genes with at least one SNP in their region (955 genes), as a function of their corresponding null distributions of $P_g^{Gene'}$ assuming a uniform distribution. Three mitochondrial genes that lie near validated T2D SNPs, as of the most recent DIAGRAM+ T2D meta-analysis are labeled in red (*IDE*, *C8orf38*, and *ACADS*). The red line marks the diagonal, and the dashed lines represent 5% and 95% confidence

intervals estimated based on 1,000 randomly sampled gene sets from the genome of identical set size to the given gene set. All gene *p*-values lie within the non-parametric 95% confidence intervals. Similar results were obtained when the observed gene *p*-values were plotted against an expected distribution that was adjusted according to a non-parametric null distribution, generated based on 1,000 randomly sampled gene sets from the genome of identical size to that of the tested gene set (data not shown). $P_g^{Gene'}$ is plotted on a $-\log_{10}$(*p*-value) scale. Note the x and y-axes of the three plots are not on the same scale.
