# Supplementary material for: Common Inherited Variation in Mitochondrial Genes Is Not Enriched for Associations with Type 2 Diabetes or Related Glycemic Traits
Source: PLoS Genet. 2010 Aug 12;6(8):e1001058. doi: 10.1371/journal.pgen.1001058 (PMC2920848; doi:10.1371/journal.pgen.1001058)
Supplement: Figure S8 — Distribution of T2D gene association p-values following correction for confounders. (A) The distribution of the unadjusted best SNP association p-value, for all genes g in the genome is shown using the Diabetes Genetics Initiative (DGI) GWA study. Since the most significant SNP in a gene region was chosen for each gene the distribution is skewed towards low p-values. (B) The distribution of all DGI gene p-values following correction for confounders using phenotype permutation analysis () demonstrates how the correction transforms into a uniform distribution. An excess of significant genes is seen at <0.001. (C) The distribution of all DGI gene p-values following correction using step-wise multivariate linear regression analysis () on the first four confounders listed in Table 1 is close to uniform, similar to (in panel B). A slight deviation from uniformity is seen for at the less significant end of the p-values. An excess of significant genes is also observed at <0.001. (D) The distribution of all gene p-values computed for the DIAGRAM+ T2D GWA meta-analysis, following step-wise linear regression of against the first five confounders listed in Table 1 () transforms the skewed distribution to a reasonably uniform one, similar to the DGI study. An excess of significant genes is also observed at <0.001. A bin of 0.001 was used for all four plots. (0.53 MB PDF) [file pgen.1001058.s008.pdf]

Figure S8

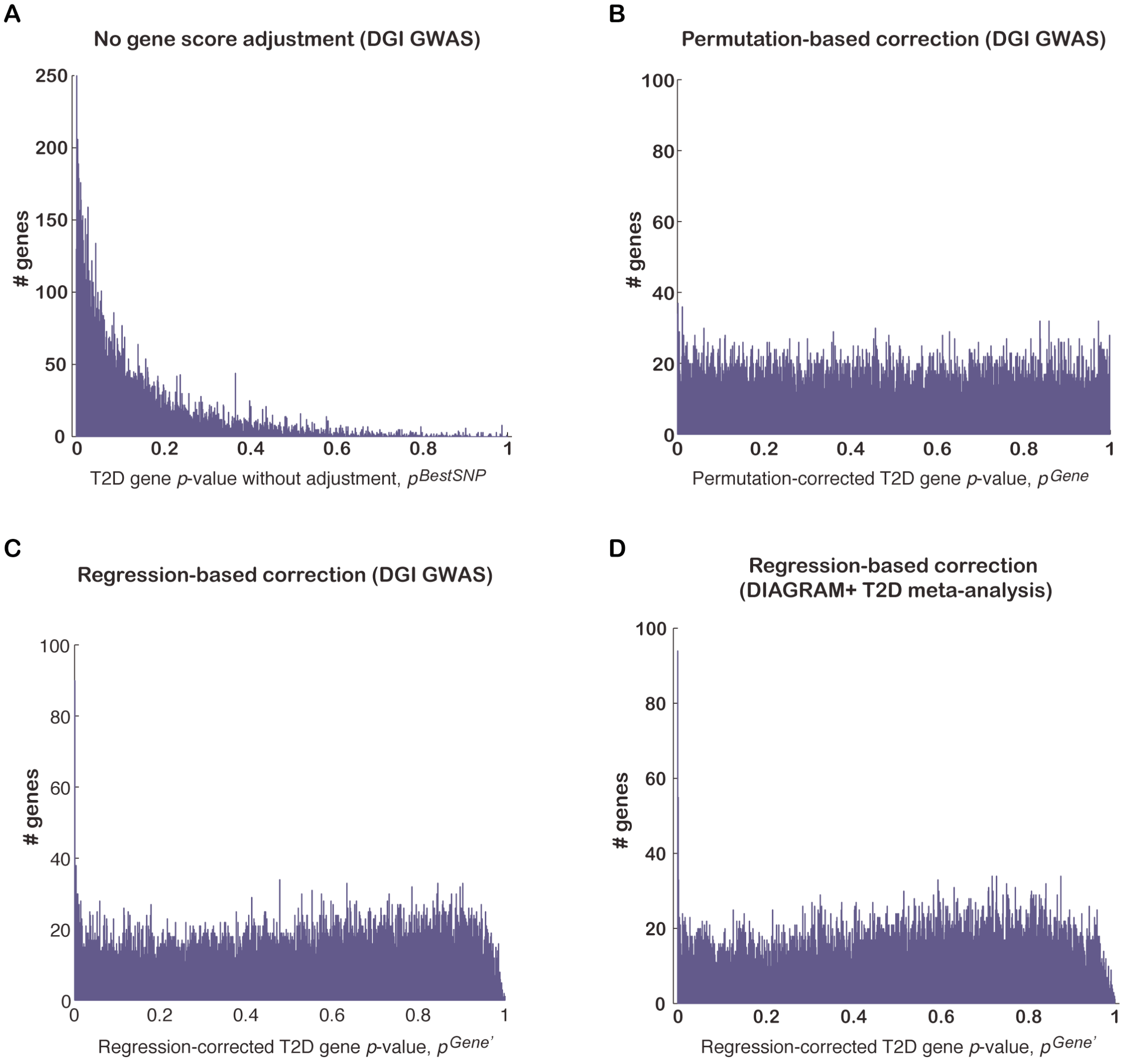

Figure S8. Distribution of T2D gene association *p*-values following correction for confounders. (A) The distribution of the unadjusted best SNP association *p*-value, $P_g^{BestSNP}$ for all genes g in the genome is shown using the Diabetes Genetics Initiative (DGI) GWA study. Since the most significant SNP in a gene region was chosen for each gene the distribution is skewed towards low *p*-values. (B) The distribution of all DGI gene *p*-values following correction for confounders using phenotype permutation analysis (P_g^{Gene}) demonstrates how the correction transforms $P_g^{BestSNP}$ into a uniform distribution.

An excess of significant genes is seen at P_g^{Gene}<0.001. (C) The distribution of all DGI gene *p*-values following correction using step-wise multivariate linear regression analysis ($P_g^{Gene'}$) on the first four confounders listed in Table 1 is close to uniform, similar to P_g^{Gene} (in panel B). A slight deviation from uniformity is seen for $P_g^{Gene'}$ at the less significant end of the *p*-values. An excess of significant genes is also observed at $P_g^{Gene'}$<0.001. (D) The distribution of all gene *p*-values computed for the DIAGRAM+ T2D GWA meta-analysis, following step-wise linear regression of $P_g^{BestSNP}$ against the first five confounders listed in Table 1 ($P_g^{Gene'}$) transforms the skewed $P_g^{BestSNP}$ distribution to a reasonably uniform one, similar to the DGI study. An excess of significant genes is also observed at $P_g^{Gene'}$<0.001. A bin of 0.001 was used for all four plots.
