# Supplementary material for: Common Inherited Variation in Mitochondrial Genes Is Not Enriched for Associations with Type 2 Diabetes or Related Glycemic Traits
Source: PLoS Genet. 2010 Aug 12;6(8):e1001058. doi: 10.1371/journal.pgen.1001058 (PMC2920848; doi:10.1371/journal.pgen.1001058)
Supplement: Figure S9 — Power of MAGENTA as a function of gene set enrichment significance threshold. We compared the effect of two p-value thresholds used to call a gene set significantly enriched in a given simulation run, on the power of MAGENTA to detect gene set enrichment. The two cutoffs tested were: <0.01 (solid line) and <0.001 (dashed line). is the nominal enrichment p-value for gene set gs. Power is plotted as a function of the fraction of causal genes that were randomly assigned a SNP with a modest effect size equivalent to 1% power of detecting an association at genome-wide significance (p-value<5e-8) using single SNP analysis. Two gene set sizes were examined: 100 genes (squares) and 1,000 genes (circles). Power appears to decrease only slightly with a more stringent GSEA p-value threshold. Note the x-axis is on a log10 scale. (0.24 MB PDF) [file pgen.1001058.s009.pdf]

Figure S9

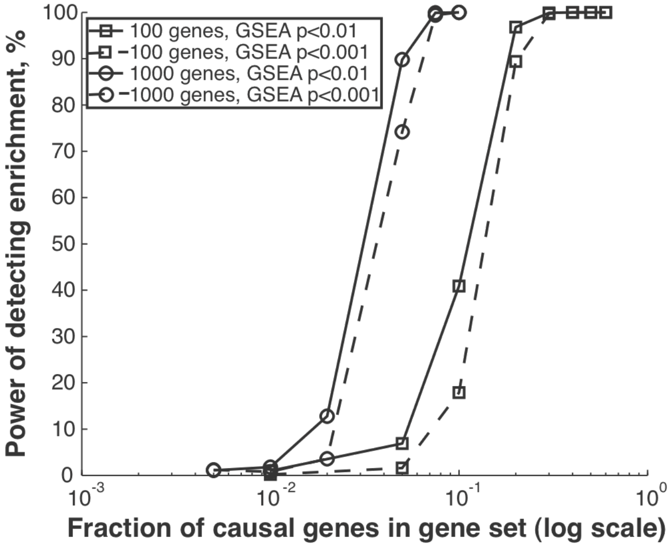

Figure S9. Power of MAGENTA as a function of gene set enrichment significance threshold. We compared the effect of two *p*-value thresholds used to call a gene set significantly enriched in a given simulation run, on the power of MAGENTA to detect gene set enrichment. The two cutoffs tested were: P_{gs}^{GSEA}<0.01 (solid line) and P_{gs}^{GSEA}<0.001 (dashed line). P_{gs}^{GSEA} is the nominal enrichment *p*-value for gene set *gs*. Power is plotted as a function of the fraction of causal genes that were randomly assigned a SNP with a modest effect size equivalent to 1% power of detecting an association at genome-wide significance (*p*-value<5e-8) using single SNP analysis. Two gene set sizes were examined: 100 genes (squares) and 1,000 genes (circles). Power appears to decrease only slightly with a more stringent GSEA *p*-value threshold. Note the x-axis is on a $\log_{10}$ scale.
