# Supplementary material for: Common Inherited Variation in Mitochondrial Genes Is Not Enriched for Associations with Type 2 Diabetes or Related Glycemic Traits
Source: PLoS Genet. 2010 Aug 12;6(8):e1001058. doi: 10.1371/journal.pgen.1001058 (PMC2920848; doi:10.1371/journal.pgen.1001058)
Supplement: Table S1 — Average gene size of nuclear-encoded mitochondrial genes compared to non-mitochondrial genes. Mitochondrial genes refer to nuclear-encoded mitochondrial genes on autosomal chromosomes taken from the MitoCarta compendium (Pagliarini DJ, et al. (2008), Cell 134: 112–123). OXPHOS genes refer to the oxidative phosphorylation gene subset. The calculations are based on the March 2006 (hg18) assembly of all human genes. bp, base pairs. (0.04 MB PDF) [file pgen.1001058.s010.pdf]

Table S1. Average gene size of nuclear-encoded mitochondrial genes compared to non-mitochondrial genes.

Gene set	# Genes	Mean gene size, bp	Median gene size, bp
Mitochondiral genes	966	38,255	18,068
OXPHOS genes	91	21,086	10,667
Non-miotchondrial genes	16,714	60,752	23,261
All genes	17,680	59,523	22,910
Mitochondrial genes / non-mitoch genes	-	0.63	0.78
OXPHOS / non-mitoch genes	-	0.35	0.46

Mitochondrial genes refer to nuclear-encoded mitochondrial genes on autosomal chromosomes taken from the MitoCarta compendium (Pagliarini DJ, et al. (2008), Cell 134: 112-123). OXPHOS genes refer to the oxidative phosphorylation gene subset. The calculations are based on the March 2006 (hg18) assembly of all human genes. bp, base pairs.
