# Supplementary material for: Common Inherited Variation in Mitochondrial Genes Is Not Enriched for Associations with Type 2 Diabetes or Related Glycemic Traits
Source: PLoS Genet. 2010 Aug 12;6(8):e1001058. doi: 10.1371/journal.pgen.1001058 (PMC2920848; doi:10.1371/journal.pgen.1001058)
Supplement: Table S2 — Correlation between T2D gene association scores, computed from DIAGRAM+ meta-analysis, and six potential confounders. Pearson's correlation coefficient (r) was calculated between the unadjusted and adjusted best SNP per gene z-scores, and , respectively, and six physical and linkage-related gene properties, using the DIAGRAM+ T2D GWA study meta-analysis. Aside for gene size, all gene properties were divided by the size of the gene plus its extended physical boundaries (150 kb was added to the most extreme transcript size for each gene, as the −110kb/+40kb extended gene boundary was used). is a vector of the uncorrected gene z-scores for all genes in genome, and is a vector of corrected gene z-scores for all genes, using step-wise multivariate linear regression analysis. All correlations between and the six variables were statistically significant (p<1e-28). †These gene properties were significant at p<0.05 under a step-wise multivariate linear regression model that regresses against all six gene properties (see Table S4 for regression model parameters and p-values). (0.20 MB PDF) [file pgen.1001058.s011.pdf]

Table S2. Correlation between T2D gene association scores, computed from DIAGRAM+ meta-analysis, and six potential confounders.

Gene property	Correlation with $Z^{BestSNP}$ (No correction)	Correlation with $Z^{Gene'}$ (Regression correction)
Gene size, kilobase (kb)†	0.20	0.007
# SNPs per kb†	0.30	-0.023
# independent SNPs per kb†	0.25	-0.001
# recombination hotspots per kb†	0.17	0.013
Linkage disequilibrium units per kb†	0.09	-0.024
Genetic distance, centi-Morgan per kb	0.08	0.028

Pearson's correlation coefficient (r) was calculated between the unadjusted and adjusted best SNP per gene z-scores, $Z^{BestSNP}$ and $Z^{Gene'}$, respectively, and six physical and linkage-related gene properties, using the DIAGRAM+ T2D GWA study meta-analysis. Aside for gene size, all gene properties were divided by the size of the gene plus its extended physical boundaries (150 kb was added to the most extreme transcript size for each gene, as the -110kb/+40kb extended gene boundary was used). $Z^{BestSNP}$ is a vector of the uncorrected gene z-scores for all genes in genome, and $Z^{Gene'}$ is a vector of corrected gene z-scores for all genes, using step-wise multivariate linear regression analysis. All correlations between $Z^{BestSNP}$ and the six variables were statistically significant (p<1e-28). †These gene properties were significant at p<0.05 under a step-wise multivariate linear regression model that regresses $Z^{BestSNP}$ against all six gene properties (see Table S4 for regression model parameters and p-values).
