# Supplementary material for: Common Inherited Variation in Mitochondrial Genes Is Not Enriched for Associations with Type 2 Diabetes or Related Glycemic Traits
Source: PLoS Genet. 2010 Aug 12;6(8):e1001058. doi: 10.1371/journal.pgen.1001058 (PMC2920848; doi:10.1371/journal.pgen.1001058)
Supplement: Table S3 — Using GWA permutations to identify significant confounders on gene scores under a multivariate regression model. For each of the 1,000 Diabetes Genetic Initiative (DGI) GWA study permutations (described in Materials and Methods) we applied step-wise multivariate linear regression analysis to the most significant SNP per gene p-value, for all genes g, against the six gene properties listed in the table. We used the fraction of permuted GWA studies for which a given gene property was included in the regression model (at p<0.05) to assess the significance of each gene property as a confounder on . GWA study permutations are not expected to contain true associations, and hence any correlation between and a gene property in a permuted dataset should be due solely to artificial or confounding effects. All gene properties aside for gene size were divided by the size of the gene and its extended physical boundaries (the gene boundaries used in this analysis were ±50kb around the gene's most extreme transcript boundaries). For all gene set analyses performed in this paper, we chose to include the gene properties that were significant under the regression model in at least ∼50% of permutations for gene score adjustment, and therefore we used the first five properties listed in this table. We obtained very similar GSEA results for all gene sets and GWA studies tested in this paper, when only the first four properties listed in the table, that were significant in almost all permutations tested, were used (data not shown). (0.13 MB PDF) [file pgen.1001058.s012.pdf]

Table S3. Using GWA permutations to identify significant confounders on gene scores under a multivariate regression model.

Gene property	Fraction of DGI GWA study permutations in which a gene property was significant under a step-wise multiple linear regression model
Gene size, kilobase (kb)	1000/1000
# SNPs/kb	1000/1000
# independent SNPs/kb	1000/1000
# recombination hotspots/kb	997/1000
Linkage disequilibrium units/kb	464/1000
Genetic distance, centi-Morgan/kb	274/1000

For each of the 1,000 Diabetes Genetic Initiative (DGI) GWA study permutations (described in Materials and Methods) we applied step-wise multivariate linear regression analysis to the most significant SNP per gene *p*-value, $P_g^{BestSNP}$ for all genes *g,* against the six gene properties listed in the table. We used the fraction of permuted GWA studies for which a given gene property was included in the regression model (at $p<0.05$) to assess the significance of each gene property as a confounder on $P_g^{BestSNP}$. GWA study permutations are not expected to contain true associations, and hence any correlation between $P_g^{BestSNP}$ and a gene property in a permuted dataset should be due solely to artificial or confounding effects. All gene properties aside for gene size were divided by the size of the gene and its extended physical boundaries (the gene boundaries used in this analysis were ±50kb around the gene's most extreme transcript boundaries). For all gene set analyses performed in this paper, we chose to include the gene properties that were significant under the regression model in at least ~50% of permutations for gene score adjustment, and therefore we used the first five properties listed in this table. We obtained very similar GSEA results for all gene sets and GWA studies tested in this paper, when only the first four properties listed in the table, that were significant in almost all permutations tested, were used (data not shown).
