# Supplementary material for: Common Inherited Variation in Mitochondrial Genes Is Not Enriched for Associations with Type 2 Diabetes or Related Glycemic Traits
Source: PLoS Genet. 2010 Aug 12;6(8):e1001058. doi: 10.1371/journal.pgen.1001058 (PMC2920848; doi:10.1371/journal.pgen.1001058)
Supplement: Table S4 — Parameters of step-wise multivariate linear regression models of T2D gene scores against gene score confounders. The parameters of a step-wise multivariate linear regression model of the best SNP p-value, (the response variable) for all genes g, on five gene properties (potential gene score confounders; the predictor variables) are listed here for the Diabetes Genetics Initiative (DGI) GWA study and the DIAGRAM+ T2D GWA meta-analysis. The confounding variables imputed into the regression model were those variables that were significant under the regression model in more than about half of the 1,000 DGI GWA permutations tested (Table S3). Hence, only the first five out of six properties listed in Table 1 were considered here. At each step of the regression analysis, an additional variable (gene score confounder) is added for consideration under the regression model. Variables with p<0.05 were considered significant and included in the regression model, and variables with p>0.1 were removed from the model. Variables are listed in the table in the order they were added to the model. Similar ß coefficients and p-values were obtained within each study using either −110kb/+40kb gene boundaries or ±50kb boundaries. The main differences between the DGI GWA study and the DIAGRAM+ meta-analysis were in the ß coefficients of SNP density and of linkage disequilibrium unit density. The ß coefficient for SNP density is smaller in the DIAGRAM+ meta-analysis compared to the DGI study, possibly because the overall SNP density is much larger in the meta-analysis (∼6-fold higher), which may decrease the difference in SNP density between small and large genes. The linkage disequilibrium unit gene property was not considered significant for the DGI study. This may also be due to differences in SNP density, since a lower SNP density may decrease the fraction of SNPs in a given chromosomal region that are in strong linkage disequilibrium. †All gene properties aside for gene size were divi [file pgen.1001058.s013.pdf]

Table S4. Parameters of step-wise multivariate linear regression models of T2D gene scores against gene score confounders.

**Diabetes Genetics Initiative (DGI) GWA study**						
**Gene boundaries:**	**-110kb 5', +40kb 3'**			**-50kb 5', +50kb 3'**		
**Gene score confounder† (Predictor variable)**	**ß coefficient**	**Standard error of ß coefficient**	***p*-value (model)***	**ß coefficient**	**Standard error of ß coefficient**	***p*-value (model)***
Gene size, kilobase (kb)	0.0009	3.7e-5	<1e-100	0.0011	3.8e-5	<1e-100
# SNPs/kb	2.74	0.08	<1e-100	2.68	0.08	<1e-100
# independent SNPs/kb	1.03	0.11	<1e-100	1.0	0.11	<1e-100
# recombination hotspots/kb	3.28	0.63	1.8e-7	2.26	0.58	9.7e-5
Linkage disequilibrium units/kb	-0.03	0.31	0.94	-0.21	0.27	0.43
**Intercept**	1.21			1.12		
**DIAGRAM+ T2D GWA study meta-analysis**						
**Gene boundaries:**	**-110kb 5', +40kb 3'**			**-50kb 5', +50kb 3'**		
**Gene score confounder† (Predictor variable)**	**ß coefficient**	**Standard error of ß coefficient**	***p*-value (model)***	**ß coefficient**	**Standard error of ß coefficient**	***p*-value (model)***
Gene size, kilobase (kb)	0.0009	4e-5	<1e-100	0.0011	4.2e-5	<1e-100
# SNPs/kb	0.35	0.02	<1e-100	0.32	0.02	<1e-100
# independent SNPs/kb	1.07	0.15	3.6e-13	1.44	0.16	<1e-100
# recombination hotspots/kb	5.80	0.79	1.7e-13	4.17	0.34	2.2e-9
Linkage disequilibrium units/kb	-1.06	0.35	0.0022	-1.78	0.70	2.3e-7
**Intercept**	1.66			1.60		

The parameters of a step-wise multivariate linear regression model of the best SNP *p*-value, $P_g^{BestSNP}$ (the response variable) for all genes *g*, on five gene properties (potential

gene score confounders; the predictor variables) are listed here for the Diabetes Genetics Initiative (DGI) GWA study and the DIAGRAM+ T2D GWA meta-analysis. The confounding variables imputed into the regression model were those variables that were significant under the regression model in more than about half of the 1,000 DGI GWA permutations tested (Table S3). Hence, only the first five out of six properties listed in Table 1 were considered here. At each step of the regression analysis, an additional variable (gene score confounder) is added for consideration under the regression model. Variables with $p<0.05$ were considered significant and included in the regression model, and variables with $p>0.1$ were removed from the model. Variables are listed in the table in the order they were added to the model. Similar ß coefficients and *p*-values were obtained within each study using either -110kb/+40kb gene boundaries or ±50kb boundaries. The main differences between the DGI GWA study and the DIAGRAM+ meta-analysis were in the ß coefficients of SNP density and of linkage disequilibrium unit density. The ß coefficient for SNP density is smaller in the DIAGRAM+ meta-analysis compared to the DGI study, possibly because the overall SNP density is much larger in the meta-analysis (~6-fold higher), which may decrease the difference in SNP density between small and large genes. The linkage disequilibrium unit gene property was not considered significant for the DGI study. This may also be due to differences in SNP density, since a lower SNP density may decrease the fraction of SNPs in a given chromosomal region that are in strong linkage disequilibrium. [†]All gene properties aside for gene size were divided by the size of the gene and its extended physical boundaries. *p-value is the probability for testing the null hypothesis that ß=0 (i.e. probability that a variable should not be added to the regression model).
