# Supplementary material for: Common Inherited Variation in Mitochondrial Genes Is Not Enriched for Associations with Type 2 Diabetes or Related Glycemic Traits
Source: PLoS Genet. 2010 Aug 12;6(8):e1001058. doi: 10.1371/journal.pgen.1001058 (PMC2920848; doi:10.1371/journal.pgen.1001058)
Supplement: Table S5 — GSEA results for lipid and lipoprotein-related pathways using LDL cholesterol GWA meta-analysis of 19,840 individuals. A total of 51 (partially overlapping) gene sets related to lipid, lipoprotein and fatty acid metabolism taken from the PANTHER and Gene Ontology databases were tested with MAGENTA for enrichment of genetic associations to LDL cholesterol blood levels, using a GWA meta-analysis of 19,840 individuals (Kathiresan S. et al., 2009, Nature Genetics 41: 56–65). GSEA p-values that passed the Bonferroni significance threshold were marked with an asterisk (each database was corrected for multiple hypothesis testing separately due to considerable overlap between the gene sets from the different databases). The Bonferroni cutoffs for the different databases are: PANTHER (15 pathways): p<0.0033, Gene Ontology, biological process terms (29 gene sets): p<0.0017, and Gene Ontology, molecular function terms (7 gene sets): p<0.0071. In the third column, GSEA p-values in parentheses are following exclusion of 19 genes that lie near 11 validated SNPs associated with LDL cholesterol (taken from Table 2 in Kathiresan S. et al., 2009). Interestingly, the association signals of some of the gene sets, including lipid and lipoprotein metabolism and lipid transport processes are still detectable when genes near validated SNPs are removed from the GSEA analysis. The 95th percentile of the adjusted LDL gene association p-values () for all genes in the genome was used as the gene set enrichment cutoff. (0.05 MB PDF) [file pgen.1001058.s014.pdf]

Table S5. GSEA results for lipid and lipoprotein-related pathways using an LDL cholesterol GWA meta-analysis of 19,840 individuals.

Gene set	# genes analyzed by GSEA	Nominal GSEA p-value	Observed # genes above 95th percentile cutoff	Expected # genes above 95th percentile cutoff	Genes near validated LDL cholesterol SNPs
**PANTHER , Biological Process**					
Fatty acid metabolism	88	0.0120 (0.0112)	10	4	-
Lipid and fatty acid transport	97	0.0218 (0.0999)	10	5	APOB, APOC1, APOC2, APOE
Acyl-CoA metabolism	17	0.0500 (0.0494)	3	1	-
Other steroid metabolism	10	0.0922	2	1	-
Steroid metabolism	52	0.2605	4	3	-
Lipid metabolism	129	0.3101	8	6	APOC2
Cholesterol metabolism	60	0.3581	4	3	HMGCR
Regulation of lipid, fatty acid and steroid metabolism	26	0.3852	2	1	-
Steroid hormone metabolism	27	0.3955	2	1	-
Phospholipid metabolism	116	0.5226	6	6	-
Lipid, fatty acid and steroid metabolism	37	0.5592	2	2	-
Lipid and fatty acid binding	16	0.5621	1	1	-
Other lipid, fatty acid and steroid metabolism	25	0.7179	1	1	-
Fatty acid beta-oxidation	23	1	0	1	-
Fatty acid biosynthesis	13	1	0	1	-
**Gene Ontology, Molecular Function**					
LIPID TRANSPORTER ACTIVITY	27	0.0090 (0.0352)	5	1	APOC4
LIPOPROTEIN BINDING	18	0.0106 (0.0466)	4	1	LDLR
LOW DENSITY LIPOPROTEIN BINDING	12	0.0215 (0.1035)	3	1	LDLR
PHOSPHOLIPID BINDING	44	0.0642	5	2	APOE
PHOSPHOLIPID TRANSPORTER ACTIVITY	12	0.1163	2	1	-
LIPID BINDING	80	0.2129	6	4	APOC2, APOE
STEROL BINDING	9	0.3714	1	0-1	-
**Gene Ontology, Biological Process**					
LIPID TRANSPORT	27	0.0001* (0.0352)	7	1	APOE, LDLR
LIPID HOMEOSTASIS	14	0.0005* (0.0204)	5	1	APOE, PCSK9
LIPOPROTEIN METABOLIC PROCESS	31	0.0010* (0.0038)	7	2	LDLR
LIPID METABOLIC PROCESS	291	0.0013* (0.0046)	27	15	APOC1, APOC2, APOC4, LDLR
FATTY ACID METABOLIC PROCESS	58	0.0019* (0.0024)	9	3	-
LIPID CATABOLIC PROCESS	36	0.0079 (0.0078)	6	2	-
REGULATION OF LIPID METABOLIC PROCESS	11	0.0140 (0.0143)	3	1	-
CELLULAR LIPID METABOLIC PROCESS	231	0.0201 (0.0355)	19	12	APOC2
FATTY ACID BIOSYNTHETIC PROCESS	12	0.0206 (0.0181)	3	1	-
FATTY ACID OXIDATION	18	0.0554 (0.0572)	3	1	-
TRIACYLGLYCEROL METABOLIC PROCESS	9	0.0749	2	0	APOC2
FATTY ACID BETA OXIDATION	11	0.1004	2	1	-
PHOSPHOLIPID METABOLIC PROCESS	70	0.1298	6	4	APOC2
LIPID BIOSYNTHETIC PROCESS	87	0.1489	7	4	-
GLYCEROPHOSPHOLIPID METABOLIC PROCESS	42	0.1549	4	2	-
STEROID METABOLIC PROCESS	65	0.2221	5	3	-
CELLULAR LIPID CATABOLIC PROCESS	33	0.2230	3	2	-
STEROID BIOSYNTHETIC PROCESS	20	0.2639	2	1	-
PHOSPHOLIPID BIOSYNTHETIC PROCESS	36	0.2739	3	2	-
MEMBRANE LIPID METABOLIC PROCESS	94	0.3292	6	5	APOC2
LIPOPROTEIN BIOSYNTHETIC PROCESS	24	0.3417	2	1	-
GLYCEROPHOSPHOLIPID BIOSYNTHETIC PROCESS	27	0.3931	2	1	-
MEMBRANE LIPID BIOSYNTHETIC PROCESS	45	0.3943	3	2	-
SPHINGOLIPID BIOSYNTHETIC PROCESS	10	0.3973	1	1	-
GLYCOSPHINGOLIPID METABOLIC PROCESS	11	0.4272	1	1	-
GLYCOLIPID METABOLIC PROCESS	14	0.5274	1	1	-
SPHINGOLIPID METABOLIC PROCESS	28	0.7668	1	1	-
BILE ACID METABOLIC PROCESS	11	1	0	1	-
SPHINGOID METABOLIC PROCESS	12	1	0	1	-

A total of 51 (partially overlapping) gene sets related to lipid, lipoprotein and fatty acid metabolism taken from the PANTHER and Gene Ontology databases were tested with MAGENTA for enrichment of genetic associations to LDL cholesterol blood levels, using a GWA meta-analysis of 19,840 individuals (Kathiresan S. et al., 2009, Nature Genetics 41: 56-65). GSEA p-values that passed the Bonferroni significance threshold were marked with an asterisk (each database was corrected for multiple hypothesis testing separately due to considerable overlap between the gene sets from the different databases). The Bonferroni cutoffs for the different databases are: PANTHER (15 pathways): $p<0.0033$, Gene Ontology, biological process terms (29 gene sets): $p<0.0017$, and Gene Ontology, molecular function terms (7 gene sets): $p<0.0071$. In the third column, GSEA p-values in parentheses are following exclusion of 19 genes that lie near 11 validated SNPs associated with LDL cholesterol (taken from Table 2 in Kathiresan S. et al., 2009). Interestingly, the association signals of some of the gene sets, including lipid and lipoprotein metabolism and lipid transport processes are still detectable when genes near validated SNPs are removed from the GSEA analysis. The 95th percentile of the adjusted LDL gene association p-values (pGene') for all genes in the genome was used as the gene set enrichment cutoff.
