# Supplementary material for: Common Inherited Variation in Mitochondrial Genes Is Not Enriched for Associations with Type 2 Diabetes or Related Glycemic Traits
Source: PLoS Genet. 2010 Aug 12;6(8):e1001058. doi: 10.1371/journal.pgen.1001058 (PMC2920848; doi:10.1371/journal.pgen.1001058)
Supplement: Table S6 — GSEA results for lipid and lipoprotein-related pathways using HDL cholesterol GWA meta-analysis of 19,840 individuals. A total of 51 (partially overlapping) gene sets related to lipid, lipoprotein and fatty acid metabolism taken from the PANTHER and Gene Ontology databases were tested with MAGENTA for enrichment of genetic associations to HDL cholesterol blood levels, using a GWA meta-analysis of 19,840 individuals (Kathiresan S. et al., 2009, Nature Genetics 41: 56–65). GSEA p-values that passed the Bonferroni significance threshold were marked with an asterisk (each database was corrected for multiple hypothesis testing separately due to considerable overlap between the gene sets from the different databases). The Bonferroni cutoffs for the different databases are: PANTHER (15 pathways): p<0.0033, Gene Ontology, biological process terms (29 gene sets): p<0.0017, and Gene Ontology, molecular function terms (7 gene sets): p<0.0071. In the third column, GSEA p-values in parentheses are following exclusion of 20 genes that lie near 14 validated SNPs associated with HDL cholesterol (taken from Table 2 in Kathiresan S. et al., 2009). Interestingly, the association signals of some of the gene sets, including lipid metabolism, binding and transport processes and triacylglycerol metabolism are still detectable when genes near validated HDL cholesterol SNPs are removed from the GSEA analysis. The 95th percentile of the adjusted HDL gene association p-values () for all genes in the genome was used as the gene set enrichment cutoff. (0.05 MB PDF) [file pgen.1001058.s015.pdf]

Table S6. GSEA results for lipid and lipoprotein-related pathways using an HDL cholesterol GWA meta-analysis of 19,840 individuals.

Gene set	# genes analyzed by GSEA	Nominal GSEA p-value	Observed # genes above 95th percentile cutoff	Expected # genes above 95th percentile cutoff	Genes near validated HDL cholesterol SNPs
**PANTHER , Biological Process**					
Lipid and fatty acid transport	99	4e-5* (0.0162)	16	5	ABCA1, APOA1, APOA4, APOC3, CETP, PLTP, APOA5
Regulation of lipid, fatty acid and steroid metabolism	26	0.1377	3	1	APOC3
Cholesterol metabolism	60	0.1821	5	3	ABCA1, CETP, LCAT, MVK
Lipid and fatty acid binding	17	0.2091	2	1	-
Acyl-CoA metabolism	18	0.2191	2	1	-
Fatty acid beta-oxidation	23	0.3207	2	1	-
Steroid hormone metabolism	24	0.3361	2	1	-
Lipid metabolism	129	0.4640	7	6	LIPC, LIPG
Phospholipid metabolism	117	0.5323	6	6	ABCA1
Fatty acid metabolism	85	0.6237	4	4	-
Other lipid, fatty acid and steroid metabolism	25	0.7224	1	1	-
Lipid, fatty acid and steroid metabolism	37	0.8492	1	2	-
Steroid metabolism	52	1	0	3	-
Fatty acid biosynthesis	13	1	0	1	-
Other steroid metabolism	10	1	0	1	-
**Gene Ontology, Molecular Function**					
LIPID BINDING	79	1.8e-5* (0.0036*)	14	4	APOA1, APOA4, CETP, APOA5
PHOSPHOLIPID BINDING	43	2.8e-5* (0.012)	10	2	APOA1, APOA4, CETP, APOA5
STEROL BINDING	9	0.0004* (0.0435)	4	0	APOA1, CETP
LIPID TRANSPORTER ACTIVITY	27	0.0426 (0.6953)	4	1	ABCA1, APOA1, APOA4, CETP
LOW DENSITY LIPOPROTEIN BINDING	12	0.119	2	1	APOA4
LIPOPROTEIN BINDING	18	0.2283	2	1	APOA4
PHOSPHOLIPID TRANSPORTER ACTIVITY	12	0.4637	1	1	ABCA1, CETP
**Gene Ontology, Biological Process**					
TRIACYLGLYCEROL METABOLIC PROCESS	9	1e-6* (8.3e-5*)	7	0	APOC3, CETP, LPL, APOA5
LIPID TRANSPORT	27	1e-6* (0.0023)	10	1	ABCA1, APOA1, APOA4, APOC3, CETP. LCAT
LIPID HOMEOSTASIS	14	1e-5* (0.0012*)	8	1	ABCA1, APOA1, APOA4, CETP, LCAT
LIPID METABOLIC PROCESS	287	6e-5* (0.0179)	31	14	APOA1, APOA4, APOA5, APOC3, CETP, HNF4A, LCAT, FADS1, FADS2, LPL, MVK, PLTP
CELLULAR LIPID METABOLIC PROCESS	229	0.0003* (0.0548)	24	11	APOA1, APOC3, CETP, LCAT, FADS1, LPL
LIPID CATABOLIC PROCESS	36	0.0006* (0.0068)	8	2	APOA4, APOA5
CELLULAR LIPID CATABOLIC PROCESS	33	0.0050 (0.0206)	6	2	APOA5
LIPID BIOSYNTHETIC PROCESS	87	0.0110 (0.2327)	10	4	APOA1, LCAT, FADS1, FADS2, MVK
LIPOPROTEIN METABOLIC PROCESS	31	0.0170 (0.4202)	5	2	APOA1, APOA4, CETP
PHOSPHOLIPID METABOLIC PROCESS	70	0.0234 (0.6511)	8	4	APOA1, CETP, LCAT, FADS1, LPL
MEMBRANE LIPID METABOLIC PROCESS	93	0.0412 (0.6551)	9	5	APOA1, CETP, LCAT, FADS1, LPL
STEROID METABOLIC PROCESS	64	0.0993	6	3	-
REGULATION OF LIPID METABOLIC PROCESS	11	0.1008	2	1	APOA5
PHOSPHOLIPID BIOSYNTHETIC PROCESS	36	0.1057	4	2	APOA1, LCAT, FADS1
GLYCEROPHOSPHOLIPID BIOSYNTHETIC PROCESS	27	0.1474	3	1	APOA1, LCAT
GLYCEROPHOSPHOLIPID METABOLIC PROCESS	42	0.1548	4	2	APOA1, CETP, LCAT
FATTY ACID METABOLIC PROCESS	57	0.1566	5	3	FADS1, FADS2
MEMBRANE LIPID BIOSYNTHETIC PROCESS	45	0.1845	4	2	APOA1, LCAT, FADS1
STEROID BIOSYNTHETIC PROCESS	20	0.2638	2	1	-
SPHINGOLIPID METABOLIC PROCESS	27	0.3875	2	1	-
FATTY ACID BETA OXIDATION	11	0.4288	1	1	-
GLYCOSPHINGOLIPID METABOLIC PROCESS	11	0.4368	1	1	-
FATTY ACID BIOSYNTHETIC PROCESS	12	0.4589	1	1	FADS1, FADS2
GLYCOLIPID METABOLIC PROCESS	14	0.5149	1	1	-
FATTY ACID OXIDATION	18	0.5978	1	1	-
LIPOPROTEIN BIOSYNTHETIC PROCESS	24	0.7094	1	1	APOA1
SPHINGOID METABOLIC PROCESS	12	1	0	1	-
SPHINGOLIPID BIOSYNTHETIC PROCESS	10	1	0	1	-
BILE ACID METABOLIC PROCESS	11	1	0	1	-

A total of 51 (partially overlapping) gene sets related to lipid, lipoprotein and fatty acid metabolism taken from the PANTHER and Gene Ontology databases were tested with MAGENTA for enrichment of genetic associations to HDL cholesterol blood levels, using a GWA meta-analysis of 19,840 individuals (Kathiresan S. et al., 2009, Nature Genetics 41: 56-65). GSEA p-values that passed the Bonferroni significance threshold were marked with an asterisk (each database was corrected for multiple hypothesis testing separately due to considerable overlap between the gene sets from the different databases). The Bonferroni cutoffs for the different databases are: PANTHER (15 pathways): $p<0.0033$, Gene Ontology, biological process terms (29 gene sets): $p<0.0017$, and Gene Ontology, molecular function terms (7 gene sets): $p<0.0071$. In the third column, GSEA p-values in parentheses are following exclusion of 20 genes that lie near 14 validated SNPs associated with HDL cholesterol (taken from Table 2 in Kathiresan S. et al., 2009). Interestingly, the association signals of some of the gene sets, including lipid metabolism, binding and transport processes and triacylglycerol metabolism are still detectable when genes near validated HDL cholesterol SNPs are removed from the GSEA analysis. The 95th percentile of the adjusted HDL gene association p-values (pGene') for all genes in the genome was used as the gene set enrichment cutoff.
