# Supplementary material for: Common Inherited Variation in Mitochondrial Genes Is Not Enriched for Associations with Type 2 Diabetes or Related Glycemic Traits
Source: PLoS Genet. 2010 Aug 12;6(8):e1001058. doi: 10.1371/journal.pgen.1001058 (PMC2920848; doi:10.1371/journal.pgen.1001058)
Supplement: Table S7 — GSEA results for lipid and lipoprotein-related pathways using triglyceride GWA meta-analysis of 19,840 individuals. A total of 51 (partially overlapping) gene sets related to lipid, lipoprotein and fatty acid metabolism taken from the PANTHER and Gene Ontology databases were tested with MAGENTA for enrichment of genetic associations to triglyceride blood levels, using a GWA meta-analysis of 19,840 individuals (Kathiresan S. et al., 2009, Nature Genetics 41: 56–65). GSEA p-values that passed the Bonferroni significance threshold were marked with an asterisk (each database was corrected for multiple hypothesis testing separately due to considerable overlap between the gene sets from the different databases). The Bonferroni cutoffs for the different databases are: PANTHER (15 pathways): p<0.0033, Gene Ontology, biological process terms (29 gene sets): p<0.0017, and Gene Ontology, molecular function terms (7 gene sets): p<0.0071. In the third column, GSEA p-values in parentheses are following exclusion from the analysis of 19 genes that lie near 11 validated SNPs associated with triglyceride levels (taken from Table 2 in Kathiresan S. et al., 2009). Interestingly, the association signals of some of the gene sets, in particular phospholipid binding and metabolic processes are still detectable when genes near validated SNPs are removed from the GSEA analysis. The 95th percentile of the adjusted triglyceride gene association p-values () for all genes in the genome was used as the gene set enrichment cutoff. (0.05 MB PDF) [file pgen.1001058.s016.pdf]

Table S7. GSEA results for lipid and lipoprotein-related pathways using triglyceride GWA meta-analysis of 19,840 individuals.

Gene set	# genes analyzed in GSEA	Nominal GSEA p-value	Observed # genes above 95th percentile cutoff	Expected # genes above 95th percentile cutoff	Genes near validated triglyceride SNPs
**PANTHER , Biological Process**					
Lipid and fatty acid transport	97	0.0490 (0.8446)	9	5	APOA1, APOA4, APOB, APOC3, LPL, PLTP, APOA5
Lipid metabolism	129	0.0529 (0.0525)	11	6	-
Other steroid metabolism	10	0.0875	2	1	-
Fatty acid metabolism	85	0.2502	6	4	-
Cholesterol metabolism	58	0.3315	4	3	-
Regulation of lipid fatty acid & steroid metabolism	26	0.3847	2	1	APOC3
Fatty acid biosynthesis	13	0.5015	1	1	-
Acyl-CoA metabolism	18	0.6045	1	1	-
Phospholipid metabolism	116	0.6891	5	6	-
Fatty acid beta-oxidation	23	0.6968	1	1	-
Other lipid, fatty acid and steroid metabolism	25	0.7212	1	1	-
Lipid, fatty acid and steroid metabolism	37	0.8456	1	2	-
Steroid metabolism	50	0.9261	1	3	-
Lipid and fatty acid binding	16	1	0	1	-
Steroid hormone metabolism	25	1	0	1	-
**Gene Ontology, Molecular Function**					
LIPID TRANSPORTER ACTIVITY	25	0.0012* (0.3238)	6	1	APOA1, APOA4
PHOSPHOLIPID BINDING	44	0.0202 (0.0548)	6	2	APOA1, APOA4, APOA5
STEROL BINDING	9	0.0752	2	0-1	APOA1
LIPID BINDING	80	0.1039	7	4	APOA1, APOA4, APOA5
PHOSPHOLIPID TRANSPORTER ACTIVITY	12	0.1143	2	1	-
LOW DENSITY LIPOPROTEIN BINDING	12	0.4555	1	1	APOA4
LIPOPROTEIN BINDING	18	0.6065	1	1	APOA4
**Gene Ontology, Biological Process**					
LIPID HOMEOSTASIS	14	0.0001* (0.0974)	5	1	APOA1, APOA4, ANGPTL3
TRIACYLGLYCEROL METABOLIC PROCESS	9	0.0008* (0.307)	4	0-1	APOC3, LPL, APOA5
LIPID TRANSPORT	26	0.0023 (0.3154)	6	1	APOA1, APOC3, ANGPTL3, APOA4
LIPOPROTEIN METABOLIC PROCESS	31	0.0044 (0.4123)	6	2	APOA1, APOA4, ANGPTL3
PHOSPHOLIPID METABOLIC PROCESS	69	0.0081 (0.0061)	9	3	APOA1, FADS1, LPL
LIPID CATABOLIC PROCESS	36	0.0083 (0.0811)	6	2	APOA4, APOA5, ANGPTL3
GLYCEROPHOSPHOLIPID METABOLIC PROCESS	42	0.0149 (0.0036)	6	2	APOA1
MEMBRANE LIPID METABOLIC PROCESS	93	0.0185 (0.0056)	10	5	APOA1, FADS1, LPL
PHOSPHOLIPID BIOSYNTHETIC PROCESS	36	0.0315 (0.007)	5	2	APOA1, FADS1
GLYCEROPHOSPHOLIPID BIOSYNTHETIC PROCESS	27	0.0423 (0.0085)	4	1	APOA1
MEMBRANE LIPID BIOSYNTHETIC PROCESS	45	0.0703	5	2	APOA1, FADS1
CELLULAR LIPID CATABOLIC PROCESS	33	0.0803	4	2	APOA5,
REGULATION OF LIPID METABOLIC PROCESS	11	0.1009	2	1	APOA5, ANGPTL3
CELLULAR LIPID METABOLIC PROCESS	230	0.1018	16	12	APOA1, APOC3, ANGPTL3, APOA5, LPL, FADS1, FADS2
LIPOPROTEIN BIOSYNTHETIC PROCESS	24	0.1129	3	1	APOA1
FATTY ACID BIOSYNTHETIC PROCESS	12	0.1255	2	1	FADS1, FADS2
LIPID BIOSYNTHETIC PROCESS	87	0.2671	6	4	APOA1, FADS1, FADS2
FATTY ACID BETA OXIDATION	11	0.4232	1	1	-
FATTY ACID METABOLIC PROCESS	57	0.5517	3	3	-
FATTY ACID OXIDATION	18	0.5994	1	1	-
STEROID METABOLIC PROCESS	63	0.8365	2	3	APOA1
BILE ACID METABOLIC PROCESS	11	1	0	1	-
GLYCOLIPID METABOLIC PROCESS	14	1	0	1	-
GLYCOSPHINGOLIPID METABOLIC PROCESS	11	1	0	1	ANGPTL3, APOA5
LIPID METABOLIC PROCESS	14	1	0	1	-
SPHINGOID METABOLIC PROCESS	12	1	0	1	-
SPHINGOLIPID BIOSYNTHETIC PROCESS	10	1	0	1	-
SPHINGOLIPID METABOLIC PROCESS	28	1	0	1	-
STEROID BIOSYNTHETIC PROCESS	20	1	0	1	-

A total of 51 (partially overlapping) gene sets related to lipid, lipoprotein and fatty acid metabolism taken from the PANTHER and Gene Ontology databases were tested with MAGENTA for enrichment of genetic associations to triglyceride blood levels, using a GWA meta-analysis of 19,840 individuals (Kathiresan S. et al., 2009, Nature Genetics 41: 56-65). GSEA p-values that passed the Bonferroni significance threshold were marked with an asterisk (each database was corrected for multiple hypothesis testing separately due to considerable overlap between the gene sets from the different databases). The Bonferroni cutoffs for the different databases are: PANTHER (15 pathways): $p<0.0033$, Gene Ontology, biological process terms (29 gene sets): $p<0.0017$, and Gene Ontology, molecular function terms (7 gene sets): $p<0.0071$. In the third column, GSEA p-values in parentheses are following exclusion from the analysis of 19 genes that lie near 11 validated SNPs associated with triglyceride levels (taken from Table 2 in Kathiresan S. et al., 2009). Interestingly, the association signals of some of the gene sets, in particular phospholipid binding and metabolic processes are still detectable when genes near validated SNPs are removed from the GSEA analysis. The 95th percentile of the adjusted triglyceride gene association p-values (pGene') for all genes in the genome was used as the gene set enrichment cutoff.
