# Supplementary material for: Common Inherited Variation in Mitochondrial Genes Is Not Enriched for Associations with Type 2 Diabetes or Related Glycemic Traits
Source: PLoS Genet. 2010 Aug 12;6(8):e1001058. doi: 10.1371/journal.pgen.1001058 (PMC2920848; doi:10.1371/journal.pgen.1001058)
Supplement: Table S8 — GSEA of LDL cholesterol GWA meta-analysis is robust to the gene score correction method used. GSEA results for lipid and lipoprotein-related gene sets using a GWA meta-analysis of LDL cholesterol blood levels (Kathiresan S. et al., 2009, Nature Genetics 41: 56–65) are presented following two different gene score correction methods: a modified version of Sidak's correction, proposed by Saconne et al. (Saccone SF et al., Human Molecular Genetics 16(1): 36–49, 2007) (column 3) and a step-wise multivariate regression analysis method (column 4). GSEA p-values that passed the Bonferroni significance threshold are marked with an asterisk (each database was corrected for multiple hypothesis testing separately, due to considerable overlap between the gene sets from the different databases). The GSEA results are quite robust to the correction method used. In the third and fourth columns, GSEA p-values in parentheses are following exclusion of 19 genes that lie near 11 validated SNPs associated with LDL cholesterol (taken from Table 2 in Kathiresan S. et al., 2009). The number of genes analyzed by MAGENTA in column 2 was taken from the analysis that applied the modified Sidak's correction of gene p-values. This number was in most cases identical to that following regression-based correction (Table S5). The 95th percentile of the adjusted LDL cholesterol gene association p-values () for all genes in the genome was used as the gene set enrichment cutoff. (0.06 MB PDF) [file pgen.1001058.s017.pdf]

Table S8. GSEA of LDL cholesterol GWA meta-analysis is robust to the gene score correction method used.

Gene set	# genes analyzed by GSEA	Nominal GSEA p-value (Modified Sidak's correction)	Nominal GSEA p-value (Regression correction)	Observed # genes above 95th percentile cutoff	Expected # genes above 95th percentile cutoff	Genes near validated LDL cholesterol SNPs
**PANTHER , Biological Process**						
Fatty acid metabolism	88	0.0123 (0.0115)	0.0120 (0.0112)	10	4	-
Lipid and fatty acid transport	97	0.0488 (0.1008)	0.0218 (0.0999)	9	5	APOB, APOC1, APOC2, APOE
Lipid metabolism	129	0.1841	0.3101	9	6	APOC2
Acyl-CoA metabolism	17	0.2056	0.0500 (0.0494)	2	1	-
Lipid, fatty acid and steroid metabolism	37	0.2758	0.5592	3	2	-
Phospholipid metabolism	116	0.3517	0.5226	7	6	-
Regulation of lipid, fatty acid and steroid metabolism	26	0.3794	0.3852	2	1	-
Other steroid metabolism	10	0.4103	0.0922	1	1	-
Steroid metabolism	52	0.4889	0.2605	3	3	-
Lipid and fatty acid binding	16	0.5591	0.5621	1	1	-
Cholesterol metabolism	60	0.5821	0.3581	3	3	HMGCR
Other lipid, fatty acid and steroid metabolism	25	0.718	0.7179	1	1	-
Steroid hormone metabolism	27	0.752	0.3955	1	1	-
Fatty acid beta-oxidation	23	1	1	0	1	-
Fatty acid biosynthesis	13	1	1	0	1	-
**Gene Ontology, Molecular Function**						
LIPID TRANSPORTER ACTIVITY	27	0.0079 (0.0394)	0.0090 (0.0352)	5	1	APOC4
LIPOPROTEIN BINDING	18	0.0127 (0.0512)	0.0106 (0.0466)	4	1	LDLR
PHOSPHOLIPID BINDING	44	0.0188 (0.0617)	0.0642	5	2	APOE
LOW DENSITY LIPOPROTEIN BINDING	12	0.0196 (0.0976)	0.0215 (0.1035)	3	1	LDLR
PHOSPHOLIPID TRANSPORTER ACTIVITY	12	0.1145	0.1163	2	1	-
LIPID BINDING	80	0.1045	0.2129	6	4	APOC2, APOE
STEROL BINDING	9	0.3627	0.3714	1	0-1	-
**Gene Ontology, Biological Process**						
LIPOPROTEIN METABOLIC PROCESS	31	0.0004* (0.0042)	0.0010* (0.0038)	7	2	LDLR
LIPID HOMEOSTASIS	14	0.0005* (0.0168)	0.0005* (0.0204)	5	1	APOE, PCSK9
LIPID TRANSPORT	27	0.0019* (0.0330)	0.0001* (0.0352)	7	1	APOE, LDLR
LIPID METABOLIC PROCESS	291	0.0086 (0.0157)	0.0013* (0.0046)	27	15	APOC1, APOC2, APOC4, LDLR
REGULATION OF LIPID METABOLIC PROCESS	11	0.0147 (0.0153)	0.0140 (0.0143)	3	1	-
FATTY ACID METABOLIC PROCESS	58	0.0258 (0.0256)	0.0019* (0.0024)	9	3	-
LIPID CATABOLIC PROCESS	36	0.0304 (0.0337)	0.0079 (0.0078)	6	2	-
PHOSPHOLIPID METABOLIC PROCESS	70	0.0597	0.1298	6	4	APOC2
GLYCEROPHOSPHOLIPID METABOLIC PROCESS	42	0.06	0.1549	4	2	-
CELLULAR LIPID METABOLIC PROCESS	231	0.064	0.0201 (0.0355)	19	12	APOC2
TRIACYLGLYCEROL METABOLIC PROCESS	9	0.07	0.0749	2	0	APOC2
PHOSPHOLIPID BIOSYNTHETIC PROCESS	36	0.1051	0.2739	3	2	-
LIPOPROTEIN BIOSYNTHETIC PROCESS	24	0.1124	0.3417	2	1	-
FATTY ACID BIOSYNTHETIC PROCESS	12	0.1153	0.0206 (0.0181)	3	1	-
GLYCEROPHOSPHOLIPID BIOSYNTHETIC PROCESS	27	0.149	0.3931	2	1	-
MEMBRANE LIPID BIOSYNTHETIC PROCESS	45	0.1901	0.3943	3	2	-
MEMBRANE LIPID METABOLIC PROCESS	94	0.1903	0.3292	6	5	APOC2
FATTY ACID OXIDATION	18	0.2242	0.0554 (0.0572)	3	1	-
LIPID BIOSYNTHETIC PROCESS	87	0.2674	0.1489	7	4	-
SPHINGOLIPID BIOSYNTHETIC PROCESS	10	0.391	0.3973	1	1	-
STEROID METABOLIC PROCESS	65	0.4066	0.2221	5	3	-
GLYCOSPHINGOLIPID METABOLIC PROCESS	11	0.4307	0.4272	1	1	-
FATTY ACID BETA OXIDATION	11	0.4328	0.1004	2	1	-
CELLULAR LIPID CATABOLIC PROCESS	33	0.502	0.2230	3	2	-
GLYCOLIPID METABOLIC PROCESS	14	0.5176	0.5274	1	1	-
STEROID BIOSYNTHETIC PROCESS	20	0.6437	0.2639	2	1	-
SPHINGOLIPID METABOLIC PROCESS	28	0.7653	0.7668	1	1	-
BILE ACID METABOLIC PROCESS	11	1	1	0	1	-
SPHINGOID METABOLIC PROCESS	12	1	1	0	1	-

GSEA results for lipid and lipoprotein-related gene sets using a GWA meta-analysis of LDL cholesterol blood levels (Kathiresan S. et al., 2009, Nature Genetics 41: 56-65) are presented following two different gene score correction methods: a modified version of Sidak's correction, proposed by Saconne et al. (Saccone SF et al., Human Molecular Genetics 16(1): 36-49, 2007) (column 3) and a step-wise multivariate regression analysis method (column 4). GSEA p-values that passed the Bonferroni significance threshold are marked with an asterisk (each database was corrected for multiple hypothesis testing separately, due to considerable overlap between the gene sets from the different databases). The GSEA results are quite robust to the correction method used. In the third and fourth columns, GSEA p-values in parentheses are following exclusion of 19 genes that lie near 11 validated SNPs associated with LDL cholesterol (taken from Table 2 in Kathiresan S. et al., 2009). The number of genes analyzed by MAGENTA in column 2 was taken from the analysis that applied the modified Sidak's correction of gene p-values. This number was in most cases identical to that following regression-based correction (Table S5). The 95th percentile of the adjusted LDL cholesterol gene association p-values (pGene') for all genes in the genome was used as the gene set enrichment cutoff.
