# Supplementary material for: Common Inherited Variation in Mitochondrial Genes Is Not Enriched for Associations with Type 2 Diabetes or Related Glycemic Traits
Source: PLoS Genet. 2010 Aug 12;6(8):e1001058. doi: 10.1371/journal.pgen.1001058 (PMC2920848; doi:10.1371/journal.pgen.1001058)
Supplement: Table S9 — GSEA of HDL cholesterol GWA meta-analysis is robust to the gene score correction method used. GSEA results for lipid and lipoprotein-related gene sets using a GWA meta-analysis of HDL cholesterol blood levels (Kathiresan S. et al., 2009, Nature Genetics 41: 56–65) are presented following two different gene score correction methods: a modified version of Sidak's correction, proposed by Saconne et al. (Saccone SF et al., Human Molecular Genetics 16(1): 36–49, 2007) (column 3) and a step-wise multivariate regression analysis method (column 4). GSEA p-values that passed the Bonferroni significance threshold are marked with an asterisk (each database was corrected for multiple hypothesis testing separately, due to considerable overlap between the gene sets from the different databases). The GSEA results are quite robust to the correction method used. GSEA p-values in parentheses are following exclusion from the analysis of 20 genes that lie near 14 validated SNPs associated with HDL cholesterol (taken from Table 2 in Kathiresan S. et al., 2009). The number of genes analyzed by MAGENTA in column 2 was taken from the analysis that used the modified Sidak's correction of gene p-values. This number was in most cases identical to that following regression-based correction (Table S5). The 95th percentile of the adjusted HDL cholesterol gene association p-values () for all genes in the genome was used as the gene set enrichment cutoff. (0.05 MB PDF) [file pgen.1001058.s018.pdf]

Table S9. GSEA of HDL cholesterol GWA meta-analysis is robust to the gene score correction method used.

Gene set	# genes analyzed by GSEA	Nominal GSEA p-value (Modified Sidak's correction)	Nominal GSEA p-value (Regression correction)	Observed # genes above 95th percentile cutoff	Expected # genes above 95th percentile cutoff	Genes near validated HDL cholesterol SNPs
**PANTHER , Biological Process**						
Lipid and fatty acid transport	99	0.0001* (0.0381)	4e-5* (0.0162)	16	5	ABCA1, APOA1, APOA4, APOC3, CETP, PLTP, APOA5
Regulation of lipid, fatty acid and steroid metabolism	26	0.1361	0.1377	3	1	APOC3
Fatty acid beta-oxidation	23	0.3184	0.3207	2	1	-
Steroid hormone metabolism	24	0.3456	0.3361	2	1	-
Cholesterol metabolism	60	0.358	0.1821	5	3	ABCA1, CETP, LCAT, MVK
Phospholipid metabolism	117	0.5397	0.5323	6	6	ABCA1
Lipid and fatty acid binding	17	0.5812	0.2091	2	1	-
Acyl-CoA metabolism	18	0.6141	0.2191	2	1	-
Fatty acid metabolism	85	0.6269	0.6237	4	4	-
Lipid metabolism	129	0.6375	0.4640	7	6	LIPC, LIPG
Other lipid, fatty acid and steroid metabolism	25	0.7143	0.7224	1	1	-
Lipid, fatty acid and steroid metabolism	37	0.8453	0.8492	1	2	-
Steroid metabolism	52	0.932	1	0	3	-
Fatty acid biosynthesis	13	1	1	0	1	-
Other steroid metabolism	10	1	1	0	1	-
**Gene Ontology, Molecular Function**						
PHOSPHOLIPID BINDING	44	0.0002* (0.0486)	2.8e-5* (0.0120)	10	2	APOA1, APOA4, CETP, APOA5
LIPID BINDING	80	0.0003* (0.0391)	1.8e-5* (0.0036*)	14	4	APOA1, APOA4, CETP, APOA5
STEROL BINDING	9	0.0006* (0.0419)	0.0004* (0.0435)	4	0	APOA1, CETP
LIPID TRANSPORTER ACTIVITY	27	0.0442 (0.6969)	0.0426 (0.6953)	4	1	ABCA1, APOA1, APOA4, CETP
LOW DENSITY LIPOPROTEIN BINDING	12	0.1203	0.1190	2	1	APOA4
LIPOPROTEIN BINDING	18	0.2241	0.2283	2	1	APOA4
PHOSPHOLIPID_TRANSPORTER_ACTIVITY	12	0.4644	0.4637	1	1	ABCA1, CETP
**Gene Ontology, Biological Process**						
LIPID TRANSPORT	27	1e-6* (0.0022)	1e-6* (0.0023)	10	1	ABCA1, APOA1, APOA4, APOC3, CETP, LCAT
LIPID HOMEOSTASIS	14	2e-6* (0.0076)	1e-5* (0.0012*)	8	1	ABCA1, APOA1, APOA4, CETP, LCAT
TRIACYLGLYCEROL METABOLIC PROCESS	9	2.6e-5* (0.0027)	1e-6* (8.3e-5*)	7	0	APOC3, CETP, LPL, APOA5
LIPID METABOLIC PROCESS	287	2.6e-4* (0.0345)	6e-5* (0.0179)	31	14	APOA1, APOA4, APOA5, APOC3, CETP, HNF4A, LCAT, FADS1, FADS2, LPL, MVK, PLTP
LIPID CATABOLIC PROCESS	36	0.0002* (0.0063)	0.0006* (0.0068)	8	2	APOA4, APOA5
CELLULAR LIPID METABOLIC PROCESS	229	0.0015* (0.0848)	0.0003* (0.0548)	24	11	APOA1, APOC3, CETP, LCAT, FADS1, LPL
CELLULAR LIPID CATABOLIC PROCESS	33	0.0048 (0.0193)	0.0050 (0.0206)	6	2	APOA5
LIPOPROTEIN METABOLIC PROCESS	31	0.0161 (0.4209)	0.0170 (0.4202)	5	2	APOA1, APOA4, CETP
PHOSPHOLIPID METABOLIC PROCESS	70	0.0217 (0.6304)	0.0234 (0.6511)	8	4	APOA1, CETP, LCAT, FADS1, LPL
MEMBRANE LIPID METABOLIC PROCESS	93	0.0424 (0.6554)	0.0412 (0.6551)	9	5	APOA1, CETP, LCAT, FADS1, LPL
LIPID BIOSYNTHETIC PROCESS	87	0.0695	0.0110 (0.2327)	10	4	APOA1, LCAT, FADS1, FADS2, MVK
STEROID METABOLIC PROCESS	64	0.0986	0.0993	6	3	-
REGULATION OF LIPID METABOLIC PROCESS	11	0.1015	0.1008	2	1	APOA5
PHOSPHOLIPID BIOSYNTHETIC PROCESS	36	0.1047	0.1057	4	2	APOA1, LCAT, FADS1
GLYCEROPHOSPHOLIPID BIOSYNTHETIC PROCESS	27	0.1517	0.1474	3	1	APOA1, LCAT
FATTY ACID METABOLIC PROCESS	57	0.1525	0.1566	5	3	FADS1, FADS2
GLYCEROPHOSPHOLIPID METABOLIC PROCESS	42	0.1558	0.1548	4	2	APOA1, CETP, LCAT
MEMBRANE LIPID BIOSYNTHETIC PROCESS	45	0.1898	0.1845	4	2	APOA1, LCAT, FADS1
STEROID BIOSYNTHETIC PROCESS	20	0.2617	0.2638	2	1	-
SPHINGOLIPID METABOLIC PROCESS	27	0.3931	0.3875	2	1	-
GLYCOSPHINGOLIPID METABOLIC PROCESS	11	0.4382	0.4368	1	1	-
FATTY ACID BETA OXIDATION	11	0.4385	0.4288	1	1	-
FATTY ACID BIOSYNTHETIC PROCESS	12	0.4598	0.4589	1	1	FADS1, FADS2
GLYCOLIPID METABOLIC PROCESS	14	0.5132	0.5149	1	1	-
FATTY ACID OXIDATION	18	0.603	0.5978	1	1	-
LIPOPROTEIN BIOSYNTHETIC PROCESS	24	0.7091	0.7094	1	1	APOA1
SPHINGOID METABOLIC PROCESS	12	1	1	0	1	-
SPHINGOLIPID BIOSYNTHETIC PROCESS	10	1	1	0	1	-
BILE ACID METABOLIC PROCESS	11	1	1	0	1	-

GSEA results for lipid and lipoprotein-related gene sets using a GWA meta-analysis of HDL cholesterol blood levels (Kathiresan S. et al., 2009, Nature Genetics 41: 56-65) are presented following two different gene score correction methods: a modified version of Sidak's correction, proposed by Saconne et al. (Saccone SF et al., Human Molecular Genetics 16(1): 36-49, 2007) (column 3) and a step-wise multivariate regression analysis method (column 4). GSEA p-values that passed the Bonferroni significance threshold are marked with an asterisk (each database was corrected for multiple hypothesis testing separately, due to considerable overlap between the gene sets from the different databases). The GSEA results are quite robust to the correction method used. GSEA p-values in parentheses are following exclusion from the analysis of 20 genes that lie near 14 validated SNPs associated with HDL cholesterol (taken from Table 2 in Kathiresan S. et al., 2009). The number of genes analyzed by MAGENTA in column 2 was taken from the analysis that used the modified Sidak's correction of gene p-values. This number was in most cases identical to that following regression-based correction (Table S5). The 95th percentile of the adjusted HDL cholesterol gene association p-values (pGene') for all genes in the genome was used as the gene set enrichment cutoff.
