# Supplementary material for: Common Inherited Variation in Mitochondrial Genes Is Not Enriched for Associations with Type 2 Diabetes or Related Glycemic Traits
Source: PLoS Genet. 2010 Aug 12;6(8):e1001058. doi: 10.1371/journal.pgen.1001058 (PMC2920848; doi:10.1371/journal.pgen.1001058)
Supplement: Table S10 — GSEA of triglyceride GWA meta-analysis is robust to the gene score correction method used. GSEA results for lipid and lipoprotein-related gene sets using a GWA meta-analysis of triglyceride blood levels (Kathiresan S. et al., 2009, Nature Genetics 41: 56–65) are presented following two different gene score correction methods: a modified version of Sidak's correction, proposed by Saconne et al. (Saccone SF et al., Human Molecular Genetics 16(1): 36–49, 2007) (column 3) and a step-wise multivariate regression analysis method (column 4). GSEA p-values that passed the Bonferroni significance threshold are marked with an asterisk (each database was corrected for multiple hypothesis testing separately, due to considerable overlap between the gene sets from the different databases). The GSEA results are quite robust to the correction method used. GSEA p-values in parentheses are following exclusion from the analysis of 19 genes that lie near 11 validated SNPs associated with triglyceride blood levels (list of known genes taken from Table 2 in Kathiresan S. et al., 2009). The number of genes analyzed by MAGENTA in column 2 was taken from the analysis that used the modified Sidak's correction method. This number was in most cases identical to that following regression-based correction (Table S5). The 95th percentile of the adjusted triglyceride gene association p-values () for all genes in the genome was used as the gene set enrichment cutoff. (0.05 MB PDF) [file pgen.1001058.s019.pdf]

Table S10. GSEA of triglyceride GWA meta-analysis is robust to the gene score correction method used.

Gene set	# genes analyzed by GSEA	Nominal GSEA p-value (Modified Sidak's correction)	Nominal GSEA p-value (Regression correction)	Observed # genes above 95th percentile cutoff	Expected # genes above 95th percentile cutoff	Genes near validated triglyceride SNPs
**PANTHER , Biological Process**						
Lipid and fatty acid transport	97	0.053 (0.8411)	0.0490 (0.8446)	9	5	APOA1, APOA4, APOB, APOC3, LPL, PLTP, APOA5
Lipid metabolism	129	0.1019	0.0529 (0.0525)	11	6	-
Other steroid metabolism	10	0.0830	0.0875	2	1	-
Fatty acid metabolism	85	0.4213	0.2502	6	4	-
Cholesterol metabolism	58	0.3252	0.3315	4	3	-
Regulation of lipid fatty acid & steroid metabolism	26	0.3836	0.3847	2	1	APOC3
Fatty acid biosynthesis	13	0.4987	0.5015	1	1	-
Acyl-CoA metabolism	18	0.6083	0.6045	1	1	-
Phospholipid metabolism	116	0.6934	0.6891	5	6	-
Fatty acid beta-oxidation	23	0.6911	0.6968	1	1	-
Other lipid, fatty acid and steroid metabolism	25	0.7186	0.7212	1	1	-
Lipid, fatty acid and steroid metabolism	37	0.8492	0.8456	1	2	-
Steroid metabolism	50	0.9225	0.9261	1	3	-
Lipid and fatty acid binding	16	1	1	0	1	-
Steroid hormone metabolism	25	1	1	0	1	-
**Gene Ontology, Molecular Function**						
LIPID_TRANSPORTER_ACTIVITY	26	0.0010* (0.0295)	0.0012* (0.3238)	6	1	APOA1, APOA4
PHOSPHOLIPID_BINDING	44	0.0202 (0.3368)	0.0202 (0.0548)	6	2	APOA1, APOA4, APOA5
STEROL_BINDING	9	0.0759	0.0752	2	0-1	APOA1
LIPID_BINDING	80	0.0986	0.1039	7	4	APOA1, APOA4, APOA5
PHOSPHOLIPID_TRANSPORTER_ACTIVITY	12	0.1205	0.1143	2	1	-
LOW_DENSITY_LIPOPROTEIN_BINDING	12	0.4614	0.4555	1	1	APOA4
LIPOPROTEIN_BINDING	18	0.5971	0.6065	1	1	APOA4
**Gene_Ontology,_Biological_Process**						
LIPID_HOMEOSTASIS	14	0.0005* (0.0946)	0.0001* (0.0974)	5	1	APOA1, APOA4, ANGPTL3
TRIACYLGLYCEROL_METABOLIC_PROCESS	9	0.0007* (0.0418)	0.0008* (0.307)	4	0-1	APOC3, LPL, APOA5
LIPID_TRANSPORT	27	0.0020 (0.325)	0.0023 (0.3154)	6	1	APOA1, APOC3, ANGPTL3, APOA4
LIPOPROTEIN_METABOLIC_PROCESS	31	0.0036 (0.1686)	0.0044 (0.4123)	6	2	APOA1, APOA4, ANGPTL3
PHOSPHOLIPID_METABOLIC_PROCESS	69	0.0063 (0.1108)	0.0081 (0.0061)	9	3	APOA1, FADS1, LPL
LIPID_CATABOLIC_PROCESS	36	0.0076 (0.2302)	0.0083 (0.0811)	6	2	APOA4, APOA5, ANGPTL3
GLYCEROPHOSPHOLIPID_METABOLIC_PROCESS	42	0.0146 (0.0544)	0.0149 (0.0036)	6	2	APOA1
MEMBRANE_LIPID_METABOLIC_PROCESS	93	0.0165 (0.1588)	0.0185 (0.0056)	10	5	APOA1, FADS1, LPL
PHOSPHOLIPID_BIOSYNTHETIC_PROCESS	36	0.035 (0.2456)	0.0315 (0.007)	5	2	APOA1, FADS1
GLYCEROPHOSPHOLIPID_BIOSYNTHETIC_PROCESS	27	0.0407 (0.1429	0.0423 (0.0085)	4	1	APOA1
CELLULAR_LIPID_METABOLIC_PROCESS	230	0.0589	0.1018	16	12	APOA1, APOC3, ANGPTL3, APOA5, LPL, FADS1, FADS2
MEMBRANE_LIPID_BIOSYNTHETIC_PROCESS	45	0.0726	0.0703	5	2	APOA1, FADS1
CELLULAR_LIPID_CATABOLIC_PROCESS	33	0.0742	0.0803	4	2	APOA5,
REGULATION_OF_LIPID_METABOLIC_PROCESS	11	0.1008	0.1009	2	1	APOA5, ANGPTL3
LIPOPROTEIN_BIOSYNTHETIC_PROCESS	24	0.1094	0.1129	3	1	APOA1
FATTY_ACID_BIOSYNTHETIC_PROCESS	12	0.1204	0.1255	2	1	FADS1, FADS2
LIPID_BIOSYNTHETIC_PROCESS	87	0.2671	0.2671	6	4	APOA1, FADS1, FADS2
FATTY_ACID_BETA_OXIDATION	11	0.4205	0.4232	1	1	-
FATTY_ACID_METABOLIC_PROCESS	57	0.5513	0.5517	3	3	-
FATTY_ACID_OXIDATION	18	0.5969	0.5994	1	1	-
STEROID_METABOLIC_PROCESS	65	0.6353	0.8365	2	3	APOA1
BILE_ACID_METABOLIC_PROCESS	11	1	1	0	1	-
GLYCOLIPID_METABOLIC_PROCESS	14	1	1	0	1	-
GLYCOSPHINGOLIPID_METABOLIC_PROCESS	11	1	1	0	1	ANGPTL3, APOA5
LIPID_METABOLIC_PROCESS	14	1	1	0	1	-
SPHINGOID_METABOLIC_PROCESS	12	1	1	0	1	-
SPHINGOLIPID_BIOSYNTHETIC_PROCESS	10	1	1	0	1	-
SPHINGOLIPID_METABOLIC_PROCESS	28	1	1	0	1	-
STEROID_BIOSYNTHETIC_PROCESS	20	1	1	0	1	-

GSEA results for lipid and lipoprotein-related gene sets using a GWA meta-analysis of triglyceride blood levels (Kathiresan S. et al., 2009, Nature Genetics 41: 56-65) are presented following two different gene score correction methods: a modified version of Sidak's correction, proposed by Saconne et al. (Saccone SF et al., Human Molecular Genetics 16(1): 36-49, 2007) (column 3) and a step-wise multivariate regression analysis method (column 4). GSEA p-values that passed the Bonferroni significance threshold are marked with an asterisk (each database was corrected for multiple hypothesis testing separately, due to considerable overlap between the gene sets from the different databases). The GSEA results are quite robust to the correction method used. GSEA p-values in parentheses are following exclusion from the analysis of 19 genes that lie near 11 validated SNPs associated with triglyceride blood levels (list of known genes taken from Table 2 in Kathiresan S. et al., 2009). The number of genes analyzed by MAGENTA in column 2 was taken from the analysis that used the modified Sidak's correction method. This number was in most cases identical to that following regression-based correction (Table S5). The 95th percentile of the adjusted triglyceride gene association p-values (pGene') for all genes in the genome was used as the gene set enrichment cutoff.
