# Supplementary material for: Common Inherited Variation in Mitochondrial Genes Is Not Enriched for Associations with Type 2 Diabetes or Related Glycemic Traits
Source: PLoS Genet. 2010 Aug 12;6(8):e1001058. doi: 10.1371/journal.pgen.1001058 (PMC2920848; doi:10.1371/journal.pgen.1001058)
Supplement: Table S13 — GSEA results of mitochondria-related gene sets are robust to GSEA statistical test used. We tested the robustness of the mitochondria-related gene set enrichment results with respect to T2D and seven diabetes-relevant glycemic traits by using an alternative GSEA statistical test to the enrichment cutoff approach. A one-tailed Mann-Whitney rank-sum test was applied (described in Materials and Methods) to GWA study meta-analyses of T2D and seven glucose and insulin-related traits. is the nominal gene set enrichment p-value for gene set gs computed for each phenotype separately. The enrichment cutoff used was the 95th percentile of all gene p-values computed from the corresponding GWA meta-analysis. The GSEA results obtained with the rank-sum approach are very similar to those obtained using the enrichment cutoff approach (see Table 3 and Table 4). HOMA-IR is an index for insulin resistance, HOMA-B is an index for β-cell function, and HbA1C represents glycated hemoglobin concentrations. OXPHOS stands for the oxidative phosphorylation process. The nuclear regulators are regulators of nuclear-encoded mitochondrial genes. ‡These gene sets are not significant after Bonferroni correction (most stringent cutoff p<0.002, given 3 gene sets and 8 traits tested; a less stringent cutoff p<0.0083, correcting for 3 gene sets and 2 traits due to considerable correlation between the glucose and insulin-related traits). (0.15 MB PDF) [file pgen.1001058.s022.pdf]

Table S13. GSEA results of mitochondria-related gene sets are robust to GSEA statistical test used.

Phenotype	Nuclear-encoded mitochondrial genes P_{gs}^{GSEA}	OXPHOS genes P_{gs}^{GSEA}	Nuclear regulators of mitochondrial genes P_{gs}^{GSEA}
Type 2 diabetes	0.4225	0.9392	0.7149
Fasting glucose	0.8600	0.9332	0.1748
Fasting insulin	0.7548	0.9987	0.0270‡
2 hour glucose	0.9154	0.9378	0.0919
2 hour insulin	0.9375	0.9232	0.1764
HOMA-IR	0.6724	0.9981	0.0335‡
HOMA-B	0.8508	0.9957	0.0285‡
HbA_{1c}	0.7989	0.5561	0.8963

We tested the robustness of the mitochondria-related gene set enrichment results with respect to T2D and seven diabetes-relevant glycemic traits by using an alternative GSEA statistical test to the enrichment cutoff approach. A one-tailed Mann-Whitney rank-sum test was applied (described in Materials and Methods) to GWA study meta-analyses of T2D and seven glucose and insulin-related traits. P_{gs}^{GSEA} is the nominal gene set enrichment *p*-value for gene set *gs* computed for each phenotype separately. The enrichment cutoff used was the 95th percentile of all gene *p*-values computed from the corresponding GWA meta-analysis. The GSEA results obtained with the rank-sum approach are very similar to those obtained using the enrichment cutoff approach (see Tables 3 and 4). HOMA-IR is an index for insulin resistance, HOMA-B is an index for β-cell function, and HbA_{1C} represents glycated hemoglobin concentrations. OXPHOS

stands for the oxidative phosphorylation process. The nuclear regulators are regulators of nuclear-encoded mitochondrial genes. [‡]These gene sets are not significant after Bonferroni correction (most stringent cutoff $p<0.002$, given 3 gene sets and 8 traits tested; a less stringent cutoff $p<0.0083$, correcting for 3 gene sets and 2 traits due to considerable correlation between the glucose and insulin-related traits).
