# Supplementary material for: Common Inherited Variation in Mitochondrial Genes Is Not Enriched for Associations with Type 2 Diabetes or Related Glycemic Traits
Source: PLoS Genet. 2010 Aug 12;6(8):e1001058. doi: 10.1371/journal.pgen.1001058 (PMC2920848; doi:10.1371/journal.pgen.1001058)
Supplement: Table S14 — GSEA results of mitochondria-related gene sets are robust to the gene score correction method used. We tested the effect of using a different gene score correction method other than the regression-based method on mitochondria-related gene set enrichment results with respect to type 2 diabetes and seven related glycemic traits. We applied a modification of the Sidak's correction (described in Materials and Methods; Saccone SF et al., Human Molecular Genetics 16(1): 36–49, 2007) to correct for confounding effects on the most significant SNP p-value, for each gene g. is the nominal gene set enrichment (GSEA) p-value for gene set gs computed for each phenotype separately. The enrichment cutoff used was the 95th percentile of all gene scores computed from the corresponding GWA study meta-analysis. HOMA-IR is an index for insulin resistance, HOMA-B is an index for β-cell function, and HbA1C represents glycated hemoglobin concentrations. OXPHOS stands for the oxidative phosphorylation process. The nuclear regulators are regulators of nuclear-encoded mitochondrial genes. ‡ This gene set is not significant after Bonferroni correction (most stringent cutoff p<0.002, given 3 gene sets and 8 traits tested; a less stringent cutoff p<0.0083, correcting for 3 gene sets and 2 traits due to considerable correlation between the glucose and insulin-related traits and type 2 diabetes). The GSEA results are comparable to those using step-wise multivariate linear regression analysis to correct for confounders on gene association p-values (Table 3 and Table 4). (0.16 MB PDF) [file pgen.1001058.s023.pdf]

Table S14. GSEA results of mitochondria-related gene sets are robust to the gene score correction method used.

Phenotype	Nuclear-encoded mitochondrial genes P_{gs}^{GSEA}	OXPHOS genes P_{gs}^{GSEA}	Nuclear regulators of mitochondrial genes P_{gs}^{GSEA}
Type 2 diabetes	0.9676	0.6649	0.1925
Fasting glucose	0.2973	0.8381	1.0000
Fasting insulin	0.4225	0.9413	0.5553
2 hour glucose	0.4128	0.4589	1.0000
2 hour insulin	0.7182	0.9462	1.0000
HOMA-IR	0.3782	0.9447	0.1897
HOMA-B	0.8108	0.8378	0.5656
HbA_{1c}	0.0556[‡]	0.8246	1.0000

We tested the effect of using a different gene score correction method other than the regression-based method on mitochondria-related gene set enrichment results with respect to type 2 diabetes and seven related glycemic traits. We applied a modification of the Sidak's correction (described in Materials and Methods; Saccone SF *et al.*, Human Molecular Genetics 16(1): 36-49, 2007) to correct for confounding effects on the most significant SNP *p*-value, $P_{g}^{BestSNP}$ for each gene *g*. P_{gs}^{GSEA} is the nominal gene set enrichment (GSEA) *p*-value for gene set *gs* computed for each phenotype separately. The enrichment cutoff used was the 95th percentile of all gene scores computed from the corresponding GWA study meta-analysis. HOMA-IR is an index for insulin resistance, HOMA-B is an index for β-cell function, and HbA_{1C} represents glycated hemoglobin concentrations. OXPHOS stands for the oxidative phosphorylation process. The nuclear regulators are regulators of nuclear-encoded mitochondrial genes. ‡ This gene set is not

significant after Bonferroni correction (most stringent cutoff $p<0.002$, given 3 gene sets and 8 traits tested; a less stringent cutoff $p<0.0083$, correcting for 3 gene sets and 2 traits due to considerable correlation between the glucose and insulin-related traits and type 2 diabetes). The GSEA results are comparable to those using step-wise multivariate linear regression analysis to correct for confounders on gene association *p*-values (Tables 3 and 4).
