# Supplementary material for: Common Inherited Variation in Mitochondrial Genes Is Not Enriched for Associations with Type 2 Diabetes or Related Glycemic Traits
Source: PLoS Genet. 2010 Aug 12;6(8):e1001058. doi: 10.1371/journal.pgen.1001058 (PMC2920848; doi:10.1371/journal.pgen.1001058)
Supplement: Text S1 — Lists of consortia participants and affiliations. (0.10 MB DOC) [file pgen.1001058.s024.doc]

**Consortia members and affiliations**

**DIAGRAM Consortium Author list**

Benjamin F Voight^1,2,3^, Laura J Scott^4^, Valgerdur Steinthorsdottir^5^, Andrew P Morris^6^, Christian Dina^7,8^, Ryan P Welch^9^, Eleftheria Zeggini^6,10^, Cornelia Huth^11,12^, Yurii S Aulchenko^13^, Gudmar Thorleifsson^5^, Laura J McCulloch^14^, Teresa Ferreira^6^, Harald Grallert^11,12^, Najaf Amin^13^, Guanming Wu^15^, Cristen J Willer^4^, Soumya Raychaudhuri^1,2,16^, Steve A McCarroll^1,17^, Claudia Langenberg^18^, Oliver M Hofmann^19^, Josée Dupuis^20^, Lu Qi^21,22^, Ayellet V Segrè^1,17^, Mandy van Hoek^23^, Pau Navarro^24^, Kristin Ardlie^1^, Beverley Balkau^25,26^, Rafn Benediktsson^27,28^, Amanda J Bennett^14^, Roza Blagieva^29^, Eric Boerwinkle^30^, Lori L Bonnycastle^31^, Kristina Bengtsson Boström^32^, Bert Bravenboer^33^, Suzannah Bumpstead^10^, Noël P Burtt^1^, Guillaume Charpentier^34^, Peter S Chines^31^, Marilyn Cornelis^22^, David J Couper^35^, Gabe Crawford^1^, Alex SF Doney^36,37^, Katherine S Elliott^6^, Amanda L Elliott^1,17,38^, Michael R Erdos^31^, Caroline S Fox^39,40^, Christopher S Franklin^41^, Martha Ganser^4^, Christian Gieger^11^, Niels Grarup^42^, Todd Green^1,2^, Simon Griffin^18^, Christopher J Groves^14^, Candace Guiducci^1^, Samy Hadjadj^43^, Neelam Hassanali^14^, Christian Herder^44^, Bo Isomaa^45,46^, Anne U Jackson^4^, Paul RV Johnson^47^, Torben Jørgensen^48,49^, Wen HL Kao^50,51^, Norman Klopp^11^, Augustine Kong^5^, Peter Kraft^21^, Johanna Kuusisto^52^, Torsten Lauritzen^53^, Man Li^50^, Aloysius Lieverse^54^, Cecilia M Lindgren^6^, Valeriya Lyssenko^55^, Michel Marre^56,57^, Thomas Meitinger^58,59^, Kristian Midthjell^60^, Mario A Morken^31^, Narisu Narisu^31^, Peter Nilsson^55^, Katharine R Owen^14^, Felicity Payne^10^, John RB Perry^61,62^, Ann-Kristin Petersen^11^, Carl Platou^60^, Christine Proença^7^, Inga Prokopenko^6,14^, Wolfgang Rathmann^63^, N William Rayner^6,14^, Neil R Robertson^6,14^, Ghislain Rocheleau^64-66^, Michael Roden^44,67^, Michael J Sampson^68^, Richa Saxena^1,2,38^, Beverley M Shields^61,62^, Peter Shrader^3,69^, Gunnar Sigurdsson^27,28^, Thomas Sparsø^42^, Klaus Strassburger^63^, Heather M Stringham^4^, Qi Sun^21^, Amy J Swift^31^, Barbara Thorand^11^, Jean Tichet^70^, Tiinamaija Tuomi^45,71^, Rob M van Dam^22^, Timon W van Haeften^72^, Thijs van Herpt^23,54^, Jana V van Vliet-Ostaptchouk^73^, G Bragi Walters^5^, Michael N Weedon^61,62^, Cisca Wijmenga^74^, Jacqueline Witteman^13^, The MAGIC investigators^75^, The GIANT consortium^76^, Richard N Bergman^77^, Stephane Cauchi^7^, Francis S Collins^78^, Anna L Gloyn^14^, Ulf Gyllensten^79^, Torben Hansen^42,80^, Winston A Hide^19^, Graham A Hitman^81^, Albert Hofman^13^, David Hunter^21^, Kristian Hveem^60,82^, Markku Laakso^52^, Karen L Mohlke^83^, Andrew D Morris^36,37^, Colin NA Palmer^36,37^, Peter P Pramstaller^84^, Igor Rudan^41,85,86^, Eric Sijbrands^23^, Lincoln D Stein^15^, Jaakko Tuomilehto^87^, Andre Uitterlinden^23^, Mark Walker^88^, Nicholas J Wareham^18^, Richard M Watanabe^77,89^, Goncalo R Abecasis^4^, Bernhard O Boehm^29^, Harry Campbell^41^, Mark J Daly^1,2^, Andrew T Hattersley^61,62^, Frank B Hu^21,22^, James B Meigs^3,69^, James S Pankow^90^, Oluf Pedersen^42,91,92^, H.-Erich Wichmann^11,12,93^, Inês Barroso^10^, Jose C Florez^1,2,3,94^, Timothy M Frayling^61,62^, Leif Groop^55,71^, Rob Sladek^64-66^, Unnur Thorsteinsdottir^5,95^, James F Wilson^41^, Thomas Illig^11^, Philippe Froguel^7,96^, Cornelia M van Duijn^13^, Kari Stefansson^5,95^, David Altshuler^1,2,3,17,38,94^, Michael Boehnke^4^, Mark I McCarthy^6,14,97^.

**DIAGRAM Consortium Affiliations**

1. Broad Institute of Harvard and Massachusetts Institute of Technology (MIT), Cambridge, Massachusetts 02142, USA
2. Center for Human Genetic Research, Massachusetts General Hospital, 185 Cambridge Street, Boston, Massachusetts 02114, USA
3. Department of Medicine, Harvard Medical School, Boston, Massachusetts 02115, USA
4. Department of Biostatistics, University of Michigan, Ann Arbor, Michigan 48109-2029, USA
5. deCODE Genetics, 101 Reykjavik, Iceland
6. Wellcome Trust Centre for Human Genetics, University of Oxford, Oxford, OX3 7BN, UK
7. CNRS-UMR-8090, Institute of Biology and Lille 2 University, Pasteur Institute, F-59019 Lille, France
8. INSERM UMR915 CNRS ERL3147 F-44007 Nantes, France
9. Bioinformatics Program, University of Michigan, Ann Arbor MI USA 48109
10. Wellcome Trust Sanger Institute, Hinxton, CB10 1HH, UK
11. Institute of Epidemiology, Helmholtz Zentrum Muenchen, 85764 Neuherberg, Germany
12. Institute of Medical Informatics, Biometry and Epidemiology, Ludwig-Maximilians-Universität, 81377 Munich, Germany
13. Department of Epidemiology, Erasmus University Medical Center, P.O. Box 2040, 3000 CA Rotterdam, The Netherlands.
14. Oxford Centre for Diabetes, Endocrinology and Metabolism, University of Oxford, OX3 7LJ, UK
15. Ontario Institute for Cancer Research, 101 College Street, Suite 800, Toronto, Ontario M5G 0A3, Canada
16. Division of Rheumatology, Immunology and Allergy, Brigham and Women's Hospital, Harvard Medical School, Boston, Massachusetts 02115, USA
17. Department of Molecular Biology, Harvard Medical School, Boston, Massachusetts 02115, USA
18. MRC Epidemiology Unit, Institute of Metabolic Science, Addenbrooke's Hospital, Cambridge CB2 0QQ, UK
19. Department of Biostatistics, Harvard School of Public Health, Boston, Massachusetts 02115, USA
20. Department of Biostatistics, Boston University School of Public Health, Boston, Massachusetts 02118, USA
21. Departments Of Nutrition and Epidemiology, Harvard School of Public Health, 665 Huntington Ave, Boston, MA 02115, USA
22. Channing Laboratory, Dept. of Medicine, Brigham and Women's Hospital and Harvard Medical School, 181 Longwood Ave, Boston, MA 02115, USA
23. Department of Internal Medicine, Erasmus University Medical Centre, PO-Box 2040, 3000 CA Rotterdam, The Netherlands
24. MRC Human Genetics Unit, Institute of Genetics and Molecular Medicine, Western General Hospital, Edinburgh, EH4 2XU, UK
25. INSERM U780, F-94807 Villejuif. France
26. University Paris-Sud, F-91405 Orsay, France
27. Landspitali University Hospital, 101 Reykjavik, Iceland
28. Icelandic Heart Association, 201 Kopavogur, Iceland
29. Division of Endocrinology, Diabetes and Metabolism, Ulm University, 89081 Ulm, Germany
30. The Human Genetics Center and Institute of Molecular Medicine, University of Texas Health Science Center, Houston, Texas 77030, USA
31. National Human Genome Research Institute, National Institute of Health, Bethesda, Maryland 20892, USA
32. R&D Centre, Skaraborg Institute, 541 30 Skövde, Sweden
33. Department of Internal Medicine, Catharina Hospital, PO-Box 1350, 5602 ZA Eindhoven, The Netherlands
34. Endocrinology-Diabetology Unit, Corbeil-Essonnes Hospital, F-91100 Corbeil-Essonnes, France
35. Department of Biostatistics and Collaborative Studies Coordinating Center, University of North Carolina at Chapel Hill, Chapel Hill, North Carolina, 27599, USA
36. Diabetes Research Centre, Biomedical Research Institute, University of Dundee, Ninewells Hospital, Dundee DD1 9SY, UK
37. Pharmacogenomics Centre, Biomedical Research Institute, University of Dundee, Ninewells Hospital, Dundee DD1 9SY, UK
38. Department of Genetics, Harvard Medical School, Boston, Massachusetts 02115, USA
39. National Heart, Lung, and Blood Institute’s Framingham Heart Study, Framingham, Massachusetts 01702, USA
40. Division of Endocrinology, Diabetes, and Hypertension, Brigham and Women’s Hospital, Harvard Medical School, Boston, Massachusetts 02115, USA
41. Centre for Population Health Sciences, University of Edinburgh, Teviot Place, Edinburgh, EH8 9AG, UK
42. Hagedorn Research Institute, **DK-2820** Gentofte, Denmark
43. CHU de Poitiers, Endocrinologie Diabetologie, CIC INSERM 0801, INSERM U927, Université de Poitiers, UFR, Médecine Pharmacie, 86021 Poitiers Cedex, France
44. Institute for Clinical Diabetology, German Diabetes Center, Leibniz Center for Diabetes Research at Heinrich Heine University Düsseldorf, 40225 Düsseldorf, Germany
45. Folkhälsan Research Center, FIN-00014 Helsinki, Finland
46. Malmska Municipal Health Center and Hospital, 68601 Jakobstad, Finland
47. DRWF Human Islet Isolation Facility and Oxford Islet Transplant Programme, University of Oxford, Old Road, Headington, Oxford, OX3 7LJ, UK
48. Research Centre for Prevention and Health, Glostrup University Hospital, DK-2600 Glostrup, Denmark
49. Faculty of Health Science, University of Copenhagen, 2200 Copenhagen, Denmark
50. Department of Epidemiology, Johns Hopkins University, Baltimore, Maryland 21287, USA
51. Department of Medicine, and Welch Center for Prevention, Epidemiology, and Clinical Research, Johns Hopkins University, Baltimore, Maryland 21287, USA
52. Department of Medicine, University of Kuopio and Kuopio University Hospital, FIN-70211 Kuopio, Finland
53. Department of General Medical Practice, University of Aarhus, DK-8000 Aarhus, Denmark
54. Department of Internal Medicine, Maxima MC, PO-Box 90052, 5600 PD Eindhoven, The Netherlands
55. Department of Clinical Sciences, Diabetes and Endocrinology Research Unit, University Hospital Malmö, Lund University, 205 02 Malmö, Sweden
56. Department of Endocrinology, Diabetology and Nutrition, Bichat-Claude Bernard University Hospital, Assistance Publique des Hôpitaux de Paris, 75870 Paris Cedex 18, France
57. INSERM U695, Université Paris 7, 75018 Paris , France
58. Institute of Human Genetics, Helmholtz Zentrum Muenchen, 85764 Neuherberg, Germany
59. Institute of Human Genetics, Klinikum rechts der Isar, Technische Universität München, 81675 Muenchen, Germany
60. HUNT Research Center, Department of Community Medicine and General Practice, Norwegian University of Science and Technology, NO-7491 Trondheim, Norway
61. Genetics of Complex Traits, Institute of Biomedical and Clinical Science, Peninsula Medical School, University of Exeter, Magdalen Road, Exeter EX1 2LU, UK
62. Diabetes Genetics, Institute of Biomedical and Clinical Science, Peninsula Medical School, University of Exeter, Barrack Road, Exeter EX2 5DW, UK
63. Institute of Biometrics and Epidemiology, German Diabetes Center, Leibniz Center for Diabetes Research at Heinrich Heine University Düsseldorf, 40225 Düsseldorf, Germany
64. Department of Human Genetics, McGill University, Montreal H3H 1P3, Canada
65. Department of Medicine, Faculty of Medicine, McGill University, Montreal, H3A 1A4, Canada
66. McGill University and Genome Quebec Innovation Centre, Montreal, H3A 1A4. Canada
67. Department of Medicine/Metabolic Diseases, Heinrich Heine University Düsseldorf, 40225 Düsseldorf, Germany
68. Department of Endocrinology and Diabetes, Norfolk and Norwich University Hospital NHS Trust , Norwich, NR1 7UY, UK.
69. General Medicine Division, Massachusetts General Hospital, Boston, Massachusetts, USA
70. Institut interrégional pour la Santé (IRSA), F-37521 La Riche, France
71. Department of Medicine, Helsinki University Hospital, University of Helsinki, FIN-00290 Helsinki, Finland
72. Department of Internal Medicine, University Medical Center Utrecht, 3584 CG Utrecht,The Netherlands
73. Molecular Genetics, Medical Biology Section, Department of Pathology and Medical Biology, University Medical Center Groningen and University of Groningen, 9700 RB Groningen, The Netherlands
74. Department of Genetics, University Medical Center Groningen and University of Groningen, 9713 EX Groningen, The Netherlands
75. The Meta-Analysis of Glucose and Insulin-related traits Consortium: for listing of members see Dupuis et al, Nature Genetics, in press
76. Genetic Investigation of Anthropometric Traits Consortium: for listing of members see Willer et al, Nature Genetics 2009
77. Department of Physiology and Biophysics, University of Southern California School of Medicine, Los Angeles, California 90033, USA
78. National Institute of Health, Bethesda, Maryland 20892, USA
79. Department of Genetics and Pathology, Rudbeck Laboratory, Uppsala University, S-751 85 Uppsala, Sweden.
80. University of Southern Denmark, DK-5230 Odense, Denmark
81. Centre for Diabetes, Barts and The London School of Medicine and Dentistry, Queen Mary University of London, London E1 2AT, UK
82. Department of Medicine, The Hospital of Levanger, N-7600 Levanger, Norway
83. Department of Genetics, University of North Carolina, Chapel Hill, North Carolina 27599, USA
84. Institute of Genetic Medicine, European Academy Bozen/Bolzano (EURAC), Viale Druso 1, 39100 Bolzano, Italy
85. Croatian Centre for Global Health, Faculty of Medicine, University of Split, Soltanska 2, 21000 Split, Croatia
86. Institute for Clinical Medical Research, University Hospital "Sestre Milosrdnice", Vinogradska 29, 10000 Zagreb, Croatia
87. Department of Chronic Disease Prevention, National Institute for Health and Welfare, Helsinki FIN-00300, Finland,
88. Diabetes Research Group, School of Clinical Medical Sciences, Newcastle University, Framlington Place, Newcastle upon Tyne NE2 4HH, UK
89. Department of Preventitive Medicine, Keck Medical School, University of Southern California, Los Angeles, CA, 90089-9001, USA
90. Division of Epidemiology and Community Health, University of Minnesota, Minneapolis, Minnesota 55454, USA
91. Department of Biomedical Science, Panum, Faculty of Health Science, University of Copenhagen, 2200 Copenhagen, Denmark
92. Faculty of Health Science, University of Aarhus, DK–8000 Aarhus, Denmark
93. Klinikum Grosshadern, 81377 Munich, Germany
94. Diabetes Unit, Massachusetts General Hospital, Boston, Massachusetts 02144, USA
95. Faculty of Medicine, University of Iceland, 101 Reykjavík, Iceland
96. Genomic Medicine, Imperial College London, Hammersmith Hospital, W12 0NN, London, UK
97. Oxford National Institute for Health Research Biomedical Research Centre, Churchill Hospital, Old Road Headington, Oxford, OX3 7LJ, UK

**MAGIC investigators**

Josée Dupuis^1,2,177^, Claudia Langenberg^3,177^, Inga Prokopenko^4,5,177^, Richa Saxena^6,7,177^, Nicole Soranzo^8,9,177^, Anne U Jackson^10^, Eleanor Wheeler^11^, Nicole LGlazer^12^, Nabila Bouatia-Naji^13^, Anna LGloyn^4^, Cecilia MLindgren^4,5^, Reedik Mägi^4,5^, Andrew P Morris^5^, Joshua Randall^5^, Toby Johnson^14–16^, Paul Elliott^17,176^, Denis Rybin^18^, Gudmar Thorleifsson^19^, Valgerdur Steinthorsdottir^19^, Peter Henneman^20^, Harald Grallert^21^, Abbas Dehghan^22^, Jouke Jan Hottenga^23^, Christopher SFranklin^24^, Pau Navarro^25^, Kijoung Song^26^, Anuj Goel^5,27^, John R B Perry^28^, Josephine MEgan^29^, Taina Lajunen^30^, Niels Grarup^31^, Thomas Sparsø^31^, Alex Doney^32^, Benjamin F Voight^6,7^, Heather MStringham^10^, Man Li^33^, Stavroula Kanoni^34^, Peter Shrader^35^, Christine Cavalcanti-Proença^13^, Meena Kumari^36^, Lu Qi^37^, Nicholas J Timpson^38^, Christian Gieger^21^, Carina Zabena^39^, Ghislain Rocheleau^40,41^, Erik Ingelsson^42,43^, Ping An^44^, Jeffrey O’Connell^45^, Jian’an Luan^3^, Amanda Elliott^6,7^, Steven A McCarroll^6,7^, Felicity Payne^11^, Rosa Maria Roccasecca^11^, François Pattou^46^, Praveen Sethupathy^47^, Kristin Ardlie^48^, Yavuz Ariyurek^49^, Beverley Balkau^50^, Philip Barter^51^, John P Beilby^52,53^, Yoav Ben-Shlomo^54^, Rafn Benediktsson^55,56^, Amanda J Bennett^4^, Sven Bergmann^14,16^, Murielle Bochud^15^, Eric Boerwinkle^57^, Amélie Bonnefond^13^, Lori LBonnycastle^47^, Knut Borch-Johnsen^58,59^, Yvonne Böttcher^60^, Eric Brunner^36^, Suzannah J Bumpstead^8^, Guillaume Charpentier^61^, Yii-Der Ida Chen^62^, Peter Chines^47^, Robert Clarke^63^, Lachlan J MCoin^17^, Matthew NCooper^64^, Marilyn Cornelis^37^, Gabe Crawford^6^, Laura Crisponi^65^, Ian NMDay^38^, Eco J Cde Geus^23^, Jerome Delplanque^13^, Christian Dina^13^, Michael R Erdos^47^, Annette CFedson^64,66^, Antje Fischer-Rosinsky^67,68^, Nita GForouhi^3^, Caroline SFox^2,69^, Rune Frants^70^, Maria Grazia Franzosi^71^, Pilar Galan^72^, Mark OGoodarzi^62^, Jürgen Graessler^73^, Christopher J Groves^4^, Scott Grundy^74^, Rhian Gwilliam^8^, Ulf Gyllensten^75^, Samy Hadjadj^76^, Göran Hallmans^77^, Naomi Hammond^8^, Xijing Han^10^, Anna-Liisa Hartikainen^78^, Neelam Hassanali^4^, Caroline Hayward^25^, Simon CHeath^79^, Serge Hercberg^80^, Christian Herder^81^, Andrew A Hicks^82^, David R Hillman^66,83^, Aroon DHingorani^36^, Albert Hofman^22^, Jennie Hui^52,84^, Joe Hung^85,86^, Bo Isomaa^87,88^, Paul R V Johnson^4,89^, Torben Jørgensen^90,91^, Antti Jula^92^, Marika Kaakinen^93^, Jaakko Kaprio^94–96^, Y Antero Kesaniemi^97^, Mika Kivimaki^36^, Beatrice Knight^98^, Seppo Koskinen^99^, Peter Kovacs^100^, Kirsten Ohm Kyvik^101^, GMark Lathrop^79^, Debbie A Lawlor^38^, Olivier Le Bacquer^13^, Cécile Lecoeur^13^, Yun Li^10^, Valeriya Lyssenko^102^, Robert Mahley^103^, Massimo Mangino^9^, Alisa KManning^1^, María Teresa Martínez-Larrad^39^, Jarred B McAteer^6,104,105^, Laura J McCulloch^4^, Ruth McPherson^106^, Christa Meisinger^21^, David Melzer^28^, David Meyre^13^, Braxton DMitchell^45^, Mario A Morken^47^, Sutapa Mukherjee^66,83^, Silvia Naitza^65^, Narisu Narisu^47^, Matthew J Neville^4,107^, Ben A Oostra^108^, Marco Orrù^65^, Ruth Pakyz^45^, Colin NA Palmer^109^, Giuseppe Paolisso^110^, Cristian Pattaro^82^, Daniel Pearson^47^, John F Peden^5,27^, Nancy LPedersen^42^, Markus Perola^96,111,112^, Andreas F H Pfeiffer^67,68^, Irene Pichler^82^, Ozren Polasek^113^, Danielle Posthuma^23,114^, Simon CPotter^8^, Anneli Pouta^115^, Michael A Province^44^, Bruce MPsaty^116,117^, Wolfgang Rathmann^118^, Nigel WRayner^4,5^, Kenneth Rice^119^, Samuli Ripatti^96,111^, Fernando Rivadeneira^22,120^, Michael Roden^81,121^, Olov Rolandsson^122^, Annelli Sandbaek^123^, Manjinder Sandhu^3,124^, Serena Sanna^65^, Avan Aihie Sayer^125^, Paul Scheet^126^, Laura J Scott^10^, Udo Seedorf^127^, Stephen J Sharp^3^, Beverley Shields^98^, Gunnar Sigurðsson^55,56^, Eric J GSijbrands^22,120^, Angela Silveira^128^, Laila Simpson^64,66^, Andrew Singleton^129^, Nicholas LSmith^130,131^, Ulla Sovio^17^, Amy Swift^47^, Holly Syddall^125^, Ann-Christine Syvänen^132^, Toshiko Tanaka^133,134^, Barbara Thorand^21^, Jean Tichet^135^, Anke Tönjes^60,136^, Tiinamaija Tuomi^87,137^, André GUitterlinden^22,120^, Ko Willems van Dijk^70,138^, Mandy van Hoek^120^, Dhiraj Varma^8^, Sophie Visvikis-Siest^139^, Veronique Vitart^25^, Nicole Vogelzangs^140^, Gérard Waeber^141^, Peter J Wagner^96,111^, Andrew Walley^142^, GBragi Walters^19^, Kim LWard^64,66^, Hugh Watkins^5,27^, Michael NWeedon^28^, Sarah H Wild^24^, Gonneke Willemsen^23^, Jaqueline CMWitteman^22^, John WGYarnell^143^, Eleftheria Zeggini^5,8^, Diana Zelenika^79^, Björn Zethelius^43,144^, Guangju Zhai^9^, Jing Hua Zhao^3^, MCarola Zillikens^120^, DIAGRAMConsortium^145^, GIANTConsortium^145^, Global BPgen Consortium^145^, Ingrid B Borecki^44^, Ruth J F Loos^3^, Pierre Meneton^80^, Patrik KEMagnusson^42^, David MNathan^104,105^, Gordon H Williams^69,105^, Andrew THattersley^98^, Kaisa Silander^96,111^, Veikko Salomaa^146^, George Davey Smith^38^, Stefan R Bornstein^73^, Peter Schwarz^73^, Joachim Spranger^67,68^, Fredrik Karpe^4,107^, Alan R Shuldiner^45^, Cyrus Cooper^125^, George V Dedoussis^34^, Manuel Serrano-Ríos^39^, Andrew DMorris^109^, Lars Lind^132^, Lyle J Palmer^64,66,84^, Frank B Hu1^47,148^, Paul WFranks^149^, Shah Ebrahim^150^, Michael Marmot^36^, WH Linda Kao^33,151,152^, James SPankow^153^, Michael J Sampson^154^, Johanna Kuusisto^155^, Markku Laakso^155^, Torben Hansen^31,156^, Oluf Pedersen^31,59,157^, Peter Paul Pramstaller^82,158,159^, H Erich Wichmann^21,160,161^, Thomas Illig^21^, Igor Rudan^24,162,163^, Alan F Wright^25^, Michael Stumvoll^60^, Harry Campbell^24^, James F Wilson^24^, Anders Hamsten on behalf of Procardis Consortium^128^, Richard NBergman^164^, Thomas A Buchanan^164,165^, Francis SCollins^47^, Karen LMohlke^166^, Jaakko Tuomilehto^94,167, 168^, Timo TValle^167^, David Altshuler^6,7,104,105^, Jerome I Rotter^62^, David SSiscovick^169^, Brenda WJ H Penninx^140^, Dorret I Boomsma^23^, Panos Deloukas^8^, Timothy DSpector^8,9^, Timothy MFrayling^28^, Luigi Ferrucci^170^, Augustine Kong^19^, Unnur Thorsteinsdottir^19,171^, Kari Stefansson^19,171^, Cornelia Mvan Duijn^22^, Yurii SAulchenko^22^, Antonio Cao^65^, Angelo Scuteri^172,177^, David Schlessinger^47^, Manuela Uda^65^, Aimo Ruokonen^173^, Marjo-Riitta Jarvelin^17,93, 174^, Dawn MWaterworth^26^, Peter Vollenweider^141^, Leena Peltonen^8,48,96,111,112^, Vincent Mooser^26^, Goncalo R Abecasis^10^, Nicholas J Wareham^3^, Robert Sladek^40,41^, Philippe Froguel^13,142^, Richard MWatanabe^164,175^, James B Meigs^35,105^, Leif Groop^102^, Michael Boehnke^10^, Mark I McCarthy^4,5,107^, Jose CFlorez^6,7,104,105^ & Inês Barroso^11^ for the MAGIC investigators

**MAGIC Affiliations**

^1^Department of Biostatistics, Boston University School of Public Health, Boston, Massachusetts, USA. ^2^National Heart, Lung, and Blood Institute’s Framingham Heart Study, Framingham, Massachusetts, USA. ^3^Medical Research Council (MRC), Epidemiology Unit, Institute of Metabolic Science, Addenbrooke’s Hospital, Cambridge, UK. ^4^Oxford Centre for Diabetes, Endocrinology and Metabolism, University of Oxford, Oxford, UK. ^5^Wellcome Trust Centre for Human Genetics, University of Oxford, Oxford, UK. ^6^Program in Medical and Population Genetics, Broad Institute, Cambridge, Massachusetts, USA. ^7^Center for Human Genetic Research, Massachusetts General Hospital, Boston, Massachusetts, USA. ^8^Wellcome Trust Sanger Institute, Hinxton, Cambridge, UK. ^9^Twin Research and Genetic Epidemiology Department, King’s College London, St Thomas’ Hospital Campus, London, UK. ^10^Center for Statistical Genetics, Department of Biostatistics, University of Michigan School of Public Health, Ann Arbor, Michigan, USA. ^11^Metabolic Disease Group, Wellcome Trust Sanger Institute, Hinxton, Cambridge, UK. ^12^Cardiovascular Health Research Unit and Department of Medicine, University of Washington, Seattle, Washington, USA. ^13^Centre National de la Recherche Scientifique–Unité Mixte de Recherche 8090, Pasteur Institute, Lille 2–Droit et Santé University, Lille, France. ^14^Department of Medical Genetics, University of Lausanne, Lausanne, Switzerland. ^15^University Institute of Social and Preventative Medicine, Centre Hospitalier Universitaire Vaudois (CHUV) and University of Lausanne, Lausanne, Switzerland. ^16^Swiss Institute of Bioinformatics, Lausanne, Switzerland. ^17^Department of Epidemiology and Public Health, Imperial College London, Faculty of Medicine, Norfolk Place, London, UK. ^18^Boston University Data Coordinating Center, Boston, Massachusetts, USA. ^19^deCODE Genetics, Reykjavik, Iceland. ^20^Department of Human Genetics, Leiden University Medical Centre, Leiden, The Netherlands. ^21^Institute of Epidemiology, Helmholtz Zentrum Muenchen, German Research Center for Environmental Health, Neuherberg, Germany. ^22^Department of Epidemiology, Erasmus Medical College, Rotterdam, The Netherlands. ^23^Department of Biological Psychology, VU University Amsterdam, Amsterdam, The Netherlands. ^24^Centre for Population Health Sciences, University of Edinburgh, Edinburgh, UK. ^25^MRC Human Genetics Unit, Institute of Genetics and Molecular Medicine, Edinburgh, UK. ^26^Division of Genetics, Research and Development, GlaxoSmithKline, King of Prussia, Pennsylvania, USA. ^27^Department of Cardiovascular Medicine, University of Oxford, Oxford, UK. ^28^Genetics of Complex Traits, Institute of Biomedical and Clinical Sciences, Peninsula College of Medicine and Dentistry, University of Exeter, Exeter, UK. ^29^National Institute of Aging, Baltimore, Maryland, USA. ^30^Unit for Child and Adolescent Health and Welfare, National Institute for Health and Welfare, Biocenter Oulu, University of Oulu, Oulu, Finland. ^31^Hagedorn Research Institute, Gentofte, Denmark. ^32^Department of Medicine and Therapeutics, Level 7, Ninewells Hospital and Medical School, Dundee, UK. ^33^Department of Epidemiology, Bloomberg School of Public Health, Johns Hopkins University, Baltimore, Maryland, USA. ^34^Department of Nutrition–Dietetics, Harokopio University, Athens, Greece. ^35^General Medicine Division, Massachusetts General Hospital, Boston, Massachusetts, USA. ^36^Department of Epidemiology and Public Health, University College London, London, UK. ^37^Departments of Nutrition and Epidemiology, Harvard School of Public Health, Boston, Massachusetts, USA. ^38^MRC Centre for Causal Analyses in Translational Epidemiology, University of Bristol, Bristol, UK. ^39^Fundación para la Investigación Biomédica del Hospital Clínico San Carlos, Madrid, Spain. ^40^Departments of Medicine and Human Genetics, McGill University, Montreal, Canada. ^41^Genome Quebec Innovation Centre, Montreal, Canada. ^42^Department of Medical Epidemiology and Biostatistics, Karolinska Institutet, Stockholm, Sweden. ^43^Department of Public Health and Caring Sciences, Uppsala University, Uppsala, Sweden. ^44^Division of Statistical Genomics, Department of Genetics, Washington University School of Medicine, St. Louis, Missouri, USA. ^45^Division of Endocrinology, Diabetes and Nutrition, University of Maryland School of Medicine, Baltimore, Maryland, USA. ^46^INSERM U859, Universite de Lille-Nord de France, Lille, France. ^47^Genome Technology Branch, National Human Genome Research Institute, Bethesda, Maryland, USA. ^48^The Broad Institute, Cambridge, Massachusetts, USA. ^49^Leiden Genome Technology Center, Leiden University Medical Center, Leiden, The Netherlands. ^50^INSERM U780, Paris Sud University, Villejuif, France. ^51^The Heart Research Institute, Sydney, New South Wales, Australia. ^52^PathWest Laboratory of Western Australia, Department of Molecular Genetics, J Block, QEII Medical Centre, Nedlands West Australia, Australia. ^53^School of Surgery and Pathology, University of Western Australia, Nedlands West Australia, Australia. ^54^Department of Social Medicine, University of Bristol, Bristol, UK. ^55^Landspitali University Hospital, Reykjavik, Iceland. ^56^Icelandic Heart Association, Kopavogur, Iceland. ^57^The Human Genetics Center and Institute of Molecular Medicine, University of Texas Health Science Center, Houston, Texas, USA. ^58^Steno Diabetes Center, Gentofte, Denmark. ^59^Faculty of Health Science, University of Aarhus, Aarhus, Denmark. ^60^Department of Medicine, University of Leipzig, Leipzig, Germany. ^61^Endocrinology–Diabetology Unit, Corbeil-Essonnes Hospital, Essonnes, France. ^62^Medical Genetics Institute, Cedars-Sinai Medical Center, Los Angeles, California, USA. ^63^Clinical Trial Service Unit and Epidemiological Studies Unit, University of Oxford, Oxford, UK. ^64^Centre for Genetic Epidemiology and Biostatistics, University of Western Australia, Perth, Australia. ^65^Istituto di Neurogenetica e Neurofarmacologia (INN), Consiglio Nazionale delle Ricerche, c/o Cittadella Universitaria di Monserrato, Monserrato, Cagliari, Italy. ^66^Western Australian Sleep Disorders Research Institute, Queen Elizabeth Medical Centre II, Perth, Australia. ^67^Department of Endocrinology, Diabetes and Nutrition, Charite-Universitaetsmedizin Berlin, Berlin, Germany. ^68^Department of Clinical Nutrition, German Institute of Human Nutrition Potsdam-Rehbruecke, Nuthetal, Germany. ^69^Division of Endocrinology, Diabetes, and Hypertension, Brigham and Women’s Hospital, Harvard Medical School, Boston, Massachusetts, USA. ^70^Department of Human Genetics, Leiden University Medical Centre, Leiden, The Netherlands. ^71^Department of Cardiovascular Research, Istituto di Ricerche Farmacologiche ‘Mario Negri’, Milan, Italy. ^72^Institut National de la Santé et de la Recherche Médicale, Institut National de la Recherche Agronomique, Université Paris 13, Bobigny Cedex, France. ^73^Department of Medicine III, Division Prevention and Care of Diabetes, University of Dresden, Dresden, Germany. ^74^Center for Human Nutrition, University of Texas Southwestern Medical Center, Dallas, Texas, USA. ^75^Department of Genetics and Pathology, Rudbeck Laboratory, Uppsala University, Uppsala, Sweden. ^76^Centre Hospitalier Universitaire, de Poitiers, Endocrinologie Diabetologie, CIC INSERM 0802, INSERM U927, Université de Poitiers, Unité de Formation et de Recherche, Médecine Pharmacie, Poitiers, France. ^77^Department of Public Health and Clinical Medicine, Section for Nutritional Research, Umeå University, Umeå, Sweden. ^78^Department of Clinical Sciences, Obstetrics and Gynecology, University of Oulu, University of Oulu, Finland. ^79^Centre National de Génotypage/Institut de génomique/Commissariat à l’énergie atomique, Evry Cedex, France. ^80^INSERM U872, Faculté de Médecine Paris Descartes, Paris Cedex, France. ^81^Institute for Clinical Diabetology, German Diabetes Center, Leibniz Center for Diabetes Research at Heinrich Heine University Düsseldorf, Düsseldorf, Germany. ^82^Institute of Genetic Medicine, European Academy Bozen/Bolzano (EURAC), Viale Druso, Bolzano, Italy, Affiliated Institute of the University Lübeck, Lübeck, Germany. ^83^Department of Pulmonary Physiology, Sir Charles Gairdner Hospital, Perth, Australia. ^84^Busselton Population Medical Research Foundation, Sir Charles Gairdner Hospital, Perth, Australia. ^85^Heart Institute of Western Australia, Sir Charles Gairdner Hospital, Nedlands West Australia, Australia. ^86^School of Medicine and Pharmacology, University of Western Australia, Nedlands West Australia, Australia. ^87^Folkhalsan Research Centre, Helsinki, Finland. ^88^Malmska Municipal Health Care Center and Hospital, Jakobstad, Finland. ^89^Nuffield Department of Surgery, University of Oxford, Oxford, UK. ^90^Research Centre for Prevention and Health, Glostrup University Hospital, Glostrup, Denmark. ^91^Faculty of Health Science, University of Copenhagen, Copenhagen, Denmark. ^92^National Institute for Health and Welfare, Unit of Population Studies, Turku, Finland. ^93^Institute of Health Sciences and Biocenter Oulu, University of Oulu, Oulu, Finland. ^94^Department of Public Health, Faculty of Medicine, University of Helsinki, Helsinki, Finland. ^95^National Institute for Health and Welfare, Unit for Child and Adolescent Mental Health, Helsinki, Finland. ^96^Institute for Molecular Medicine Finland (FIMM), University of Helsinki, Helsinki, Finland. ^97^Department of Internal Medicine and Biocenter Oulu, Oulu, Finland. ^98^Diabetes Genetics, Institute of Biomedical and Clinical Science, Peninsula College of Medicine and Dentistry, University of Exeter, Exeter, UK. ^99^National Institute for Health and Welfare, Unit of Living Conditions, Health and Wellbeing, Helsinki, Finland. ^100^Interdisciplinary Centre for Clinical Research, University of Leipzig, Leipzig, Germany. ^101^The Danish Twin Registry, Epidemiology, Institute of Public Health, University of Southern Denmark, Odense, Denmark. ^102^Department of Clinical Sciences, Diabetes and Endocrinology, Lund University, University Hospital Malmö, Malmö, Sweden. ^103^Gladstone Institute of Cardiovascular Disease, University of California, San Francisco, California, USA. ^104^Diabetes Research Center, Diabetes Unit, Massachusetts General Hospital, Boston, Massachusetts, USA. ^105^Department of Medicine, Harvard Medical School, Boston, Massachusetts, USA. ^106^Division of Cardiology, University of Ottawa Heart Institute, Ottawa, Ontario, Canada. ^107^Oxford National Institute for Health Research, Biomedical Research Centre, Churchill Hospital, Oxford, UK. ^108^Department of Clinical Genetics, Erasmus Medical College, Rotterdam, The Netherlands. ^109^Biomedical Research Institute, University of Dundee, Ninewells Hospital and Medical School, Dundee, UK. ^110^Department of Geriatric Medicine and Metabolic Disease, Second University of Naples, Naples, Italy. ^111^National Institute for Health and Welfare, Unit of Public Health Genomics, Helsinki, Finland. ^112^Department of Medical Genetics, University of Helsinki, Helsinki, Finland. ^113^Department of Medical Statistics, Epidemiology and Medical Informatics, Andrija Stampar School of Public Health, Medical School, University of Zagreb, Rockefellerova, Zagreb, Croatia. ^114^Department of Clinical Genetics, VU University and Medical Center, Amsterdam, The Netherlands. ^115^Department of Obstetrics and Gynaecology, Oulu University Hospital, Oulu, Finland. ^116^Departments of Medicine, Epidemiology and Health Services, University of Washington, Seattle, Washington, USA. ^117^Group Health Research Institute, Group Health Cooperative, Seattle, Washington, USA. ^118^Institute of Biometrics and Epidemiology, German Diabetes Centre, Leibniz Centre at Heinrich Heine University Düsseldorf, Düsseldorf, Germany. ^119^Department of Biostatistics, University of Washington, Seattle, Washington, USA. ^120^Department of Internal Medicine, Erasmus Medical College, Rotterdam, The Netherlands. ^121^Department of Metabolic Diseases, Heinrich Heine University Düsseldorf, Düsseldorf, Germany. ^122^Department of Public Health and Clinical Medicine, Section for Family Medicine, Umeå University, Umeå, Sweden. ^123^School of Public Health, Department of General Practice, University of Aarhus, Aarhus, Denmark. ^124^Department of Public Health and Primary Care, Strangeways Research Laboratory, University of Cambridge, Cambridge, UK. ^125^MRC Epidemiology Resource Centre, University of Southampton, Southampton General Hospital, Southampton, UK. ^126^Department of Epidemiology, University of Texas, M.D. Anderson Cancer Center, Houston, Texas, USA. ^127^Leibniz-Institut für Arterioskleroseforschung an der Universität Münster, Münster, Germany. ^128^Atherosclerosis Research Unit, Department of Medicine, Karolinska Institutet, Stockholm, Sweden. ^129^Laboratory of Neurogenetics, National Institute on Aging, Bethesda, Maryland, USA. ^130^Department of Epidemiology, University of Washington, Seattle, Washington, USA. ^131^Seattle Epidemiologic Research and Information Center, Department of Veterans Affairs Office of Research and Development, Seattle, Washington, USA. ^132^Department of Medical Sciences, Uppsala University, Uppsala, Sweden. ^133^Medstar Research Institute, Baltimore, Maryland, USA. ^134^Clinical Research Branch, National Institute on Aging, Baltimore, Maryland, USA. ^135^Institut interrégional pour la santé (IRSA), La Riche, France. ^136^Coordination Centre for Clinical Trials, University of Leipzig, Leipzig, Germany. ^137^Department of Medicine, Helsinki University Hospital, University of Helsinki, Helsinki, Finland. ^138^Department of Internal Medicine, Leiden University Medical Centre, Leiden, The Netherlands. ^139^Research Unit, Cardiovascular Genetics, Nancy University Henri Poincaré, Nancy, France. ^140^EMGO Institute for Health and Care Research, Department of Psychiatry, VU University Medical Center, Amsterdam, The Netherlands. ^141^Department of Internal Medicine, Centre Hospitalier Universitaire Vaudois, Lausanne, Switzerland. ^142^Genomic Medicine, Imperial College London, Hammersmith Hospital, London, UK. ^143^Epidemiology and Public Health, Queen’s University Belfast, Belfast, UK. ^144^Medical Products Agency, Uppsala, Sweden. ^145^See Supplementary Note for a full list of authors. ^146^National Institute for Health and Welfare, Unit of Chronic Disease Epidemiology and Prevention, Helsinki, Finland. ^147^Departments of Nutrition and Epidemiology, Harvard School of Public Health, Boston, Massachusetts, USA. ^148^Channing Laboratory, Brigham and Women’s Hospital and Harvard Medical School, Boston, Massachusetts, USA. ^149^Genetic Epidemiology and Clinical Research Group, Department of Public Health and Clinical Medicine, Section for Medicine, Umeå University Hospital, Umeå, Sweden. ^150^London School of Hygiene and Tropical Medicine, London, UK. ^151^Department of Medicine, School of Medicine, Johns Hopkins University, Baltimore, Maryland, USA. ^152^The Welch Center for Prevention, Epidemiology, and Clinical Research, School of Medicine and Bloomberg School of Public Health, Johns Hopkins University, Baltimore, Maryland, USA. ^153^Division of Epidemiology and Community Health, School of Public Health, University of Minnesota, Minneapolis, Minnesota, USA. ^154^Department of Endocrinology and Diabetes, Norfolk and Norwich University Hospital National Health Service Trust, Norwich, UK. ^155^Department of Medicine, University of Kuopio and Kuopio University Hospital, Kuopio, Finland. ^156^Faculty of Health Science, University of Southern Denmark, Odense, Denmark. ^157^Institute of Biomedical Science, Faculty of Health Science, University of Copenhagen, Copenhagen, Denmark. ^158^Department of Neurology, General Central Hospital, Bolzano, Italy. ^159^Department of Neurology, University of Lübeck, Lübeck, Germany. ^160^Institute of Medical Informatics, Biometry and Epidemiology, Ludwig-Maximilians-Universität, Munich, Germany. ^161^Klinikum Grosshadern, Munich, Germany. ^162^School of Medicine, University of Split, Split, Croatia. ^163^Gen-Info Ltd., Zagreb, Croatia. ^164^Department of Physiology and Biophysics, Keck School of Medicine, University of Southern California, Los Angeles, California, USA. ^165^Department of Medicine, Division of Endocrinology, Keck School of Medicine, University of Southern California, Los Angeles, California, USA. ^166^Department of Genetics, University of North Carolina, Chapel Hill, North Carolina, USA. ^167^National Institute for Health and Welfare, Unit of Diabetes Prevention, Helsinki, Finland. ^168^South Ostrobothnia Central Hospital, Seinajoki, Finland. ^169^Departments of Medicine and Epidemiology, University of Washington, Seattle, Washington, USA. ^170^Longitudinal Studies Section, Clinical Research Branch, National Institute on Aging, NIH, Baltimore, Maryland, USA. ^171^Faculty of Medicine, University of Iceland, Reykjavík, Iceland. ^172^Lab of Cardiovascular Sciences, National Institute on Aging, National Institutes of Health, Baltimore, Maryland, USA. ^173^Department of Clinical Sciences/Clinical Chemistry, University of Oulu, University of Oulu, Oulu, Finland. ^174^National Institute of Health and Welfare, Oulu, Finland. ^175^Department of Preventive Medicine, Keck School of Medicine, University of Southern California, Los Angeles, California, USA. ^176^MRC–Health Protection Agency Centre for Environment and Health, Imperial College London, London, UK. ^177^UOC Geriatria, Istituto Nazionale Ricovero e cura per Anziani (INRCA) IRCCS, Rome, Italy.
